# Bio-Based and Biodegradable Polymeric Materials for a Circular Economy

**DOI:** 10.3390/polym16213015

**Published:** 2024-10-28

**Authors:** Víctor Oliver-Cuenca, Valentina Salaris, Pedro Francisco Muñoz-Gimena, Ángel Agüero, Mercedes A. Peltzer, Victoria Alcázar Montero, Marina P. Arrieta, Jaume Sempere-Torregrosa, Cristina Pavon, Maria Dolores Samper, Gema Rodríguez Crespo, Jose M. Kenny, Daniel López, Laura Peponi

**Affiliations:** 1 Instituto de Ciencia y Tecnología de Polímeros, ICTP-CSIC, Calle Juan de la Cierva 3, 28006 Madrid, Spain; victor.oc@ictp.csic.es (V.O.-C.); v.salaris@ictp.csic.es (V.S.); pfmunoz@ictp.csic.es (P.F.M.-G.); gema@ictp.csic.es (G.R.C.); 2 Instituto Universitario de Tecnología de Materiales (IUTM), Universitat Politècnica de València (UPV), Plaza Ferrándiz y Carbonell 1, 03801 Alcoy, Spain; anagrod@epsa.upv.es; 3 Departamento de Ingeniería Química Industrial y del Medio Ambiente, Escuela Técnica Superior de Ingenieros Industriales, Universidad Politécnica de Madrid (ETSII-UPM), Calle José Gutiérrez Abascal 2, 28006 Madrid, Spain; mariavictoria.alcazar@upm.es (V.A.M.); m.arrieta@upm.es (M.P.A.); 4 Laboratory of Obtention, Modification, Characterization, and Evaluation of Materials (LOMCEM), Department of Science and Technology, University of Quilmes, Bernal B1876BXD, Argentina; mercedes.peltzer@unq.edu.ar; 5 National Scientific and Technical Research Council (CONICET), Buenos Aires C1425FQB, Argentina; 6 Grupo de Investigación en Polímeros, Caracterización y Aplicaciones (POLCA), 28006 Madrid, Spain; 7 Instituto de Tecnología de Materiales (ITM), Universitat Politècnica de València (UPV), Plaza Ferrándiz y Carbonell 1, 03801 Alcoy, Spain; jausemto@epsa.upv.es (J.S.-T.); cripava1@epsa.upv.es (C.P.); masammad@upv.es (M.D.S.); 8 STM Group, University of Perugia, Strada Pentima 4, 05100 Terni, Italy; jose.kenny@unipg.it

**Keywords:** sustainable polymers, bio-based polymers, biodegradable polymers, revalorization, circular economy, nanocomposites, plasticizers, natural polymers, nanoparticles, degradation, processing

## Abstract

Nowadays, plastic contamination worldwide is a concerning reality that can be addressed with appropriate society education as well as looking for innovative polymeric alternatives based on the reuse of waste and recycling with a circular economy point of view, thus taking into consideration that a future world without plastic is quite impossible to conceive. In this regard, in this review, we focus on sustainable polymeric materials, biodegradable and bio-based polymers, additives, and micro/nanoparticles to be used to obtain new environmentally friendly polymeric-based materials. Although biodegradable polymers possess poorer overall properties than traditional ones, they have gained a huge interest in many industrial sectors due to their inherent biodegradability in natural environments. Therefore, several strategies have been proposed to improve their properties and extend their industrial applications. Blending strategies, as well as the development of composites and nanocomposites, have shown promising perspectives for improving their performances, emphasizing biopolymeric blend formulations and bio-based micro and nanoparticles to produce fully sustainable polymeric-based materials. The Review also summarizes recent developments in polymeric blends, composites, and nanocomposite plasticization, with a particular focus on naturally derived plasticizers and their chemical modifications to increase their compatibility with the polymeric matrices. The current state of the art of the most important bio-based and biodegradable polymers is also reviewed, mainly focusing on their synthesis and processing methods scalable to the industrial sector, such as melt and solution blending approaches like melt-extrusion, injection molding, film forming as well as solution electrospinning, among others, without neglecting their degradation processes.

## 1. Introduction

**Figure 1:**
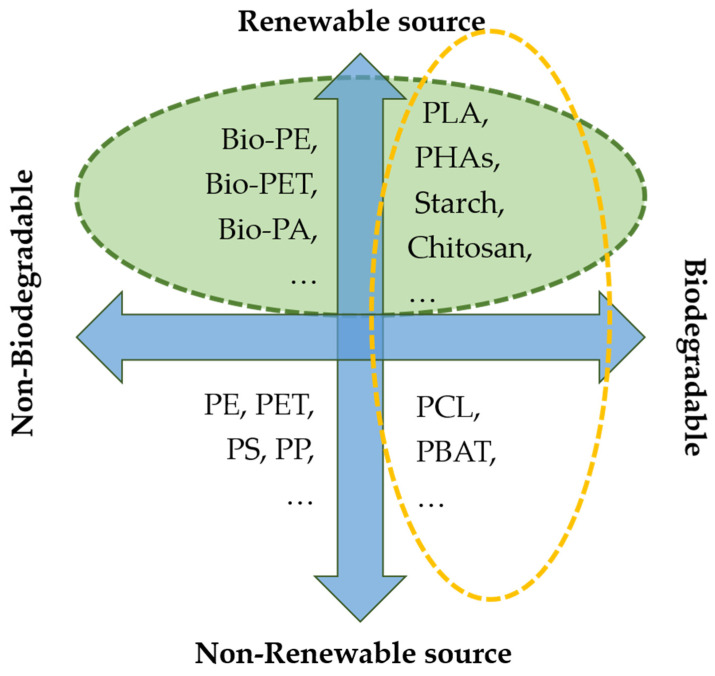
Different classes of polymers.

The use of plastics, which are cheap and versatile materials with a broad range of properties and applications, has strongly increased in the last decades. Since the beginning, traditional polymers have been used as “structural” and “single-use” materials. Moreover, fossil-based polymers such as polyethylene terephthalate (PET), polyethylene (PE), polyvinylchloride (PVC), polypropylene (PP), and polystyrene (PS) have been widely used in different sectors such as construction, electronics, packaging, automotive, sensors, etc. However, their long degradation rate and the large amount of waste generated represent critical points that cannot be neglected in their future development. For this reason, many efforts are now focused on the study of bio-based and biodegradable polymers and their potential industrial application. Moreover, it is important to consider both their processing methods and their end-of-life. Not all bio-based polymers are biodegradable, and some fossil-based polymers can be biodegradable (Figure 1). For example, bio-polyethylene is produced from bio-based sugarcane derivatives but is not biodegradable, whilst polycaprolactone (PCL) is a biodegradable non-bio-based polymer [1].

Moreover, many bio-based polymers can be extracted from agro-food waste, increasing their added values upscaling. In particular, plastic demand is expected to double before 2050, leading to an increase in global pollution caused by plastic waste and greenhouse gas emissions. Various options are taken into account to overcome this issue, including plastic recycling and the use of bio-based and biodegradable polymers [2].

Nowadays, two are the key points regarding plastics: the attention must be focused on designing multifunctional polymeric materials able to be used on demand, as well as we need to shift our effort from a linear to a circular economy, taking into account also the end-of-life of polymer-based materials, as the idea of having a world without plastic is quite impossible.

**Scheme 1:**
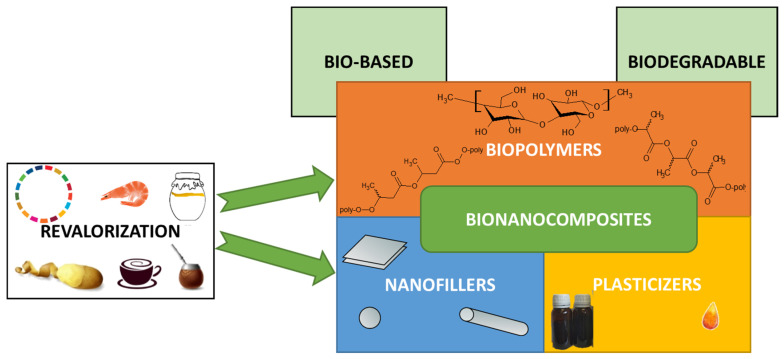
Review contents.

In this Review, we summarize the current state of the art of the most important bio-based and biodegradable polymer improvements reported in the literature, preferably in the last twenty-four years, as well as their main results in terms of blends and/or composites, nanocomposites polymeric materials. We highlight not only the importance of using a good polymeric matrix but also the effect of the addition of micro/nanoparticles and the use of vegetable oils as additives in terms of plasticizers and compatibilizers from a sustainable point of view. In particular, this Review is mainly focused on their synthesis and processing methods scalable to the industrial sector, such as melt blending approaches (melt-extrusion, injection molding, film forming) as well as solution electrospinning, among others, without neglecting to discuss the importance of their degradation process looking for a right balance between industrial interests and environmental issues, as summarized in Scheme 1.

**Figure 2:**
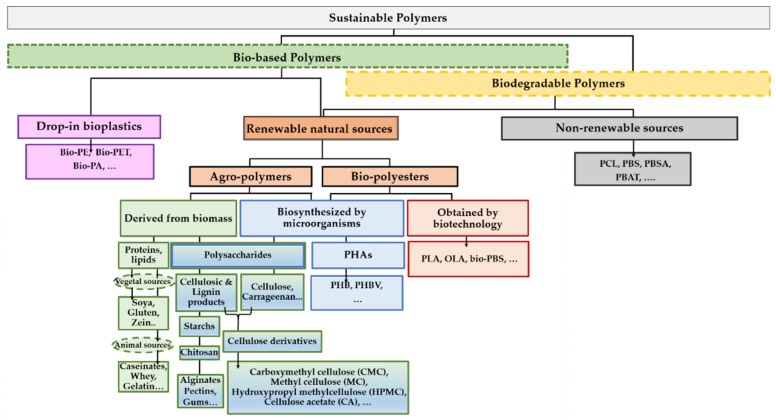
Classification of sustainable polymers according to their sources.

Nowadays, the terms biopolymers, biomass, bio-based, biocompatible, biodegradable as well as bioplastic are widely used. However, it is important to note, as recommended by IUPAC [3], that biopolymers are macromolecules formed by living organisms. At the same time, the use of bioplastics is discouraged, preferably using the expression “bio-based” polymers [4]. Biodegradable polymers, which can be obtained either from renewable resources or from chemical synthesis, can be degraded by living organisms into non-toxic byproducts [5], which can be obtained either from renewable resources or from chemical synthesis. Degradation is carried out by microorganisms producing H_2_O, CO_2_, and methane at different rates according to polymer structure, crystallinity, morphology, and molecular weight [6]. However, to avoid confusion, we define sustainable polymers as all the bio-based and biodegradable polymeric materials, as reported in Figure 2. In particular, sustainable polymers, both bio-based and biodegradable, can be divided into three main groups according to their source, such as drop-in bioplastics derived from renewable or non-renewable sources [1,7] (i.e., cellulose, starch, and chitosan are biopolymers derived from natural sources such as plants and animals [8], biodegradable polymers as polyesters such as poly(lactic acid) (PLA) and PCL, polycarbonates, polyamides, and polyanhydrides, produced by chemical synthesis [9] and polymers produced by microorganisms including polyhydroxyalkanoates (PHAs), polyhydroxybutyrate (PHB), poly(3-hydroxybutyrate-co-3-hydroxyvalerate) (PHBV), bacterial cellulose, dextran, etc. [7].

Furthermore, bio-based polymers find applications as antioxidant and antimicrobial materials [10] in tissue engineering [11], medical and food applications [12], water treatment [13], etc.

Nonetheless, bio-based polymers are characterized by poor mechanical properties, low chemical resistance, and reduced processing windows. To improve these features, biopolymeric composites, and nanocomposites are produced by adding nano or micro reinforcements, as particles or fibers, to obtain a synergistic effect and enhanced properties [14]. Another approach is the fabrication of blends with other bio-based polymers [15] or with synthetic polymers such as PE, polyamides (nylon), PS, synthetic rubber, PVC, etc., to produce materials with both increased processability, resistance, and impact strength but also with shorten degradation of the material [16].

**Figure 3:**
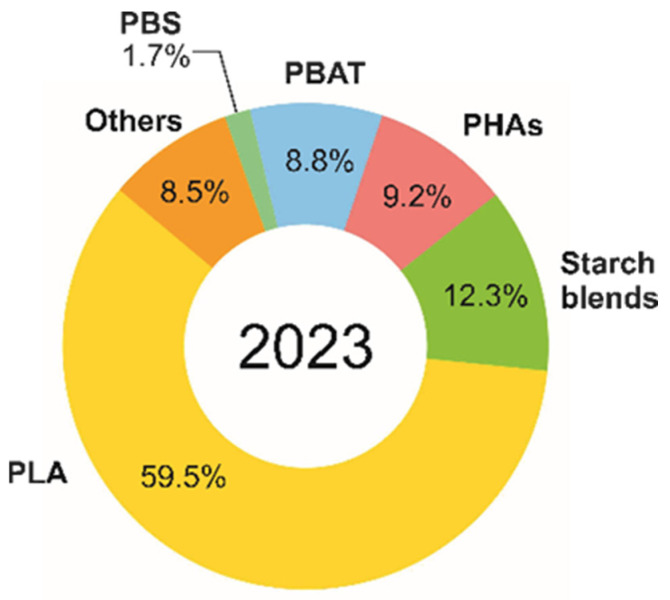
Global production capacities of biodegradable/compostable plastics by material type in 2023, adapted from European-Bioplastics-Association, 2023.

The production of bioplastics has increased again in 2023 after stagnation in previous years, and a significant increase is expected in the coming years from 2.18 million tons in 2023 to approximately 7.43 million tons in 2028. Moreover, it is noteworthy that 52.1% of bioplastics are biodegradable, with PLA being the most produced, as can be seen in Figure 3 [17].

Numerous researchers have studied bio-based and/or biodegradable polymeric bends in recent years to modify different properties. A solution to improve the miscibility of these materials is the use of different types of plasticizers and compatibilizers to improve miscibility between the polymers of the blend and certain properties, such as mechanical properties, to obtain a more competitive material.

Moreover, another interesting approach to improve the polymer’s performance to extend its industrial application is the development of bionanocomposites, which refer to composites based on a biopolymeric matrix loaded with nanofillers that present at least one dimension in the nanoscale range (below 100 nm) [18]. In this regard, if good nanofillers dispersion is achieved within the polymeric matrix, the nanosized fillers can considerably improve some specific properties of the biopolymeric matrices into the final bionanocomposites through strong and large polymer/nanofillers interactions. Bionanocomposite production offers a multitude of advantages as nanosized particles can enhance the mechanical properties, thermal stability, and barrier properties of polymers, as well as they can also provide the final bionanocomposite with specific functionalities as active (e.g., antimicrobial or antioxidant properties) or smart capability (e.g., shape memory, self-healing, or sensitivity to external stimuli) making the resulting bionanocomposites extremely versatile and suitable for several industrial applications at the same time that they are inherently more sustainable than nanocomposites based on traditional plastics derived from oil. Thus, bionanocomposites offer a promising pathway to achieving several of the Sustainable Development Goals by providing sustainable and high-performance polymeric materials that can replace conventional composites based on traditional plastics in many areas such as biomedical, healthcare, construction, electronics, packaging, agricultural applications, and water and air remediation. The introduction of bionanocomposites into the industrial sector not only supports environmental preservation but also promotes economic and social sustainability.

**Figure 4:**
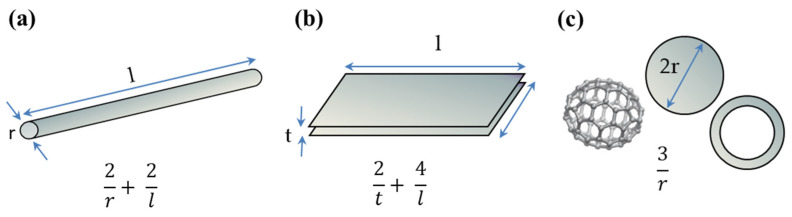
Nanofiller geometries and their respective surface area-to-volume ratios. (**a**) two-dimensional (2D); (**b**) one-dimensional (1D); and (**c**) zero-dimensional (0D) nanofillers.

Nanofillers, Figure 4, are mainly classified into three categories according to their sizes and shapes on the dimensionality of the nanosized particles [18,19,20]: (i) two-dimensional nanofillers (2D) or nanofibrous with two dimensions beyond the nanoscale range that exhibit a diameter below 100 nm and have an aspect ratio of at least 100 (Figure 4a). The most well-known 2D nanoparticles are carbon nanotubes and nanocellulose substrates (cellulose nanofibers, nanocrystals, or nanorods); (ii) one-dimensional nanofillers (1D) with only one dimension in the nanometer scale, are layered materials (nanolayers) classically with a thickness on the order of 1 nm, but with an aspect ratio following their two remaining dimensions of at least 25 (Figure 4b). The most popular 1D nanofillers are layered silicates, graphene, and covalent organic frameworks; and (iii) zero-dimensional nanofillers (0D) or referred to as equiaxed (isodimensional) nanoparticles with all dimensions confined below 100 nm (Figure 4c). This category includes nanoparticles such as silica nanoparticles and nanosized metal oxides, buckyballs, and quantum dots.

In the case of 2D and 1D nanofillers, the surface area/volume is governed by the first term in the equation. The second term (2/*l* and 4/*l*, respectively) has a minimal influence and is commonly omitted compared to the first one. Therefore, changing the nanofiller diameter (changing either the layer thickness or fiber diameter) from the micrometer to the nanometer scale will affect the surface area-to-volume ratio by three orders of magnitude. Meanwhile, for 0D nanofillers, the smaller the nanofiller diameter, the greater the surface area per unit volume [18].

Nanofillers can additionally be classified depending on their pathway as (i) natural, produced through natural processes in the environment (e.g., minerals, volcanic dust, etc.), (ii) incidental, as a waste of industrial processes (e.g., coal combustion, welding fumes, diesel exhaust, etc.) and (iii) engineered nanoparticles, produced either by “top-down approach” mainly by milling a large sample to obtain nanosized particles of the starting material, or by “bottom-up” approach, assembling smaller subunits through crystal growth or chemical synthesis from atoms or molecules up to nanofillers, better-controlling sizes, shapes, and size ranges [19,20].

Many nanosized fillers based on bio-based polymers can be produced by “top-down approaches,” starting from a polymer and reaching nanosized particles based on it. Similarly, bionanocomposites production are mainly produced by “top-down approaches” as the ingredients (polymer, nanoparticles, and other additives) are macroscopically mixed either at laboratory scale level by dissolving the polymer and dispersing the nanoparticle within the polymeric matrix and further allowing the solvent to evaporate (solvent casting method) or produced by industrial processing technologies based on melt blending approaches. In this regard, the major challenge in the development of nanocomposite materials is to achieve nanosized particle dispersion within the polymeric matrix [20]; this is why other additives are sometimes incorporated to facilitate the nanosized particles dispersion within the polymeric matrix, such as the case of plasticizers. As plasticizers increase the polymer chain mobility, they can easily increase the nanoparticle distribution along the polymeric matrix.

The most important bio-based and biodegradable polymeric matrices and nanosized particles extracted from them are summarized in this Review. Moreover, the strategy to improve polymers’ performance through the development of bionanocomposites is reviewed, as well as the different strategies used to improve the dispersion of nanosized particles within the polymeric matrix and the top-down processing technologies to produce bionanocomposites. Finally, the potential applications of such bionanocomposites are discussed and highlighted in relation to both industrial interests and environmental issues.

In order to obtain optimization in the biopolymeric blends as well as on the bionanocomposites, however, we also have to consider the importance of plasticizers and/or compatibilizers in the composition of the materials and in their final properties. In fact, plasticizers are essential additives in polymer formulation, playing a fundamental role in improving their physical and mechanical properties. They are organic compounds characterized by their ability to intercalate between polymeric chains, reducing intermolecular forces of attraction and facilitating sliding between them [21]. For this reason, they increase the flexibility, malleability, and processability of polymers as they modify viscosity, decrease the glass transition temperature, and enhance the ductile properties of polymers [22].

Plasticizers can be classified as primary or secondary plasticizers, depending on their primary function in the polymer formulation. Primary plasticizers provide flexibility, impact resistance, and tear resistance by using a single plasticizing compound that is miscible with the polymer. However, they may pose challenges in terms of thermal stability, migration, and environmental compatibility. The most commonly used primary plasticizers are phthalates such as diisononyl phthalate (DINP) and diisodecyl phthalate (DIDP), although other examples include adipates, such as diocyl adipate, sebacates like dioctyl sebacate, azelates, trimellitates, phosphoric acid esters, or epoxy softeners [23]. On the other hand, secondary plasticizers are less polar than primary ones and have lower compatibility with polymers. Being less soluble and having low compatibility, they are usually employed in combination with primary plasticizers. The mixture of both plasticizers provides a more stable blend against migration [23,24].

The benefits of using plasticizers are copious. Firstly, they enable the production of polymers with a wide range of mechanical and thermal properties, making them suitable for a variety of industrial applications, from flexible packaging to medical devices. Additionally, plasticizers can enhance the durability and impact resistance of materials, contributing to a longer lifespan and better service performance. Finally, plasticizers can also facilitate the processing and manufacturing of polymers, reducing production costs and increasing efficiency in the supply chain.

However, it is important to consider that plasticizer migration can occur due to factors such as temperature, pressure, exposure to chemicals, or contact with food and bodily fluids. This migration can lead to environmental contamination and human exposure to these compounds, raising concerns about potential toxicity and long-term health effects [25]. Phthalate plasticizers are a concern due to their migration and toxicity. As a result, both the European Union and the United States, through the Environmental Protection Agency (EPA) and the Food and Drug Administration (FDA), respectively, have prohibited or restricted the use of several phthalates in various products, especially those intended for children or for storing food, cosmetics, or medications [24,26].

A promising alternative is the use of natural plasticizers, as they exhibit low toxicity and migration. In this group, glycerol [27], vegetable oils [28], sugars such as sorbitol [29], or citric acid [30], among others, can be found. The use of natural plasticizers has not only been explored as a potential substitution for phthalates but is also associated with the growing interest in the development of new polymeric materials.

In a wide range of applications, biodegradable polymers are recognized as excellent candidates due to their environmentally friendly decomposition process. To broaden their applicability, it is necessary to adapt some of their intrinsic properties. Enhancing flexibility and reducing brittleness address the material’s limitations in various application contexts [31]. Similarly, modifying processing conditions, such as reducing the melt viscosity, facilitates easier molding and shaping, thereby expanding their usability across diverse manufacturing processes [32]. Furthermore, optimizing thermal properties to meet specific requirements while ensuring the polymers’ biodegradability is preserved is crucial for their effective use in industries ranging from packaging and agriculture to medical devices, where their degradation process does not contribute to pollution [33]. These adaptations are pivotal for integrating biodegradable polymers into sectors that demand environmental sustainability alongside functional performance [34].

Therefore, in this Review, the most important bio-based and biodegradable polymeric matrices, plasticizers, and compatibilizers, as well as nanosized particles extracted from natural sources, are summarized. In particular, the strategy to improve polymer performance through the development of bionanocomposites is reviewed, as well as the different strategies used to improve the dispersion of nanosized particles within the polymeric matrix, as well as the importance of the use of vegetable oils as additives. In particular, 10 different bio-based and/or biodegradable polymers are discussed, such as PLA, PBAT, PHAs, Starch, Cellulose, Lignin, Chitosan, Gelatin, polybutylene succinate (PBS), poly(itaconic acid), summarizing their synthesis and processing approaches. Moreover, for every polymer, the last part of the discussion is dedicated to their degradation process, which is considered a very important aspect of their end-of-life from a circular economy perspective. Therefore, in the case of natural polymers, such as starch, cellulose, and lignin, we discuss the main results in terms of extraction of nanofillers from them, evidencing their double face to be used as polymeric matrices from one side and as nanofillers from the other one.

## 2. Polylactic Acid

Polylactic acid (PLA) is an aliphatic polyester (2-hydroxypropionic acid) that was synthesized for the first time in 1932 by Carothers [35]. PLA is a biodegradable, bio-based, and biocompatible polymer derived from natural and renewable resources, such as agricultural waste [36]. This polymer is constituted by monomers of lactic acid, which are present as D and L enantiomers. Indeed, three types of PLA are present: poly-_L_-lactic acid (PLLA), poly-_D_-lactic acid (PDLA), and poly-_D,L_-lactic acid (PDLLA) [37].

The content of enantiomers affects the properties of the polymer; PLLA and PDLA are semi-crystalline polyesters with a melting temperature (T_m_) of around 175 °C and a glass transition temperature (T_g_) of 55–60 °C, while higher content of D-isomer in PDLLA leads to the formation of amorphous materials with T_g_ of 55–60 °C [18,38].

The homopolymer presents four polymorphic crystalline structures, α, α’, β, and γ, which can be formed in different crystallization conditions [39]. In particular, the α-phase is the most common and stable crystalline structure and can be obtained from the melt or solution, while the α’-phase is produced at lower crystallization temperatures and can be converted to the more stable α-phase at temperatures above 120 °C [40]. The β-phase is formed by deforming the α-form at high temperature and high drawing ratio, whereas the γ-form is obtained through epitaxial crystallization [41].

The crystallinity of PLA influences its barrier properties. Indeed, PLA presents CO_2_ and O_2_ permeability coefficients similar to PS and PET but with lower barrier properties against water vapor [42]. The crystallinity of the polymer also affects its properties. PLA presents high modulus (3 GPa) and tensile strength (50–70 MPa). However, it has a brittle nature [43,44].

**Figure 5:**
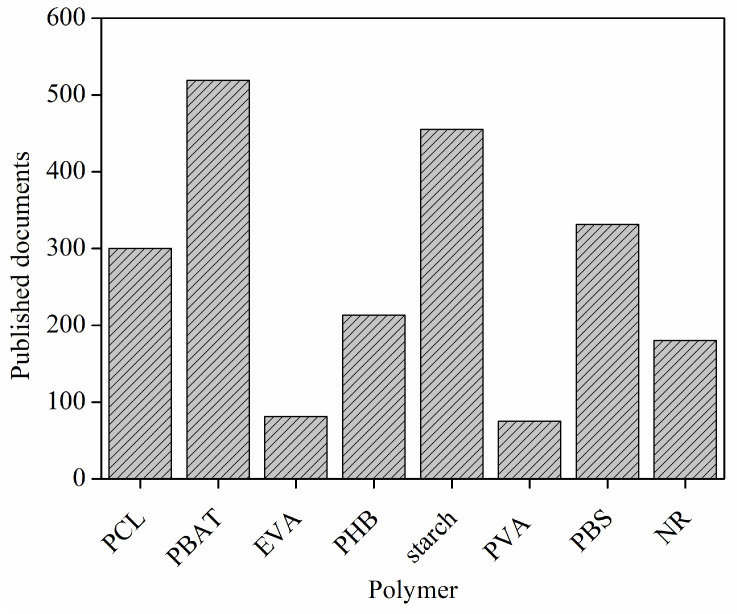
Number of publications of PLA blends (Scopus Source up to June 2024).

In order to improve the mechanical properties, in particular its toughness, blends of PLA with more flexible polymers have been produced, [45,46]. From 1988 to 2024, many works have been published based on PLA blends, as reported in Figure 5.

In addition, various organic and inorganic reinforcements such as carbon, metals, fibers, clays, and natural materials have been incorporated into the polymeric matrix to improve its permeability mechanical, antimicrobial, and thermal properties [47,48].

**Figure 6:**
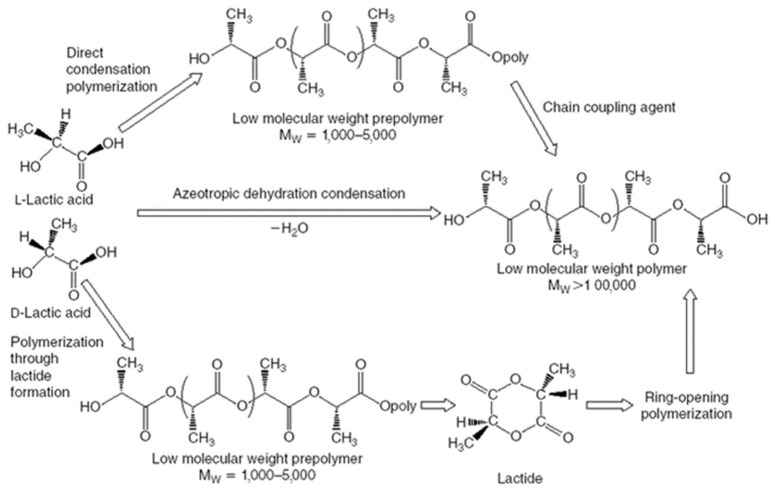
Synthetic routes of PLA synthesis, adapted from [49].

Three routes have been reported for the synthesis of PLA (Mw > 10,000), as shown in Figure 6, involving (a) direct condensation polymerization, (b) azeotropic dehydration condensation, and (c) lactic ring opening polymerization (ROP), which is the most common process for the production of PLA with high molecular weight [49].

Polycondensation leads to the fabrication of polymeric chains with low molecular weights due to the increased viscosity of the polymer melt and the presence of impurities and water as byproducts [50].

Azeotropic dehydration condensation permits the production of high molecular weight PLA without using chain extenders and esterification adjuvants. It involves a distillation of lactic acid for 2–3 h at 130 °C under reduced pressure. Subsequently, catalyst and diphenyl ether are added, then a molecular sieve is used, and the solvent is returned to the container for a further 30–40 h at 130 °C. Finally, the polymer is separated for further purification [51].

ROP implicates polycondensation, depolymerization, and ring opening of the LA lactide. Although this route needs several purification steps and requires the use of heavy metal catalysts (i.e., Tin(II) octoate, Sn(Oct)2)) [52], it is the most industrially used to obtain high molecular weight PLA [18].

On the other hand, the synthesis of shorter polymer chains of poly lactic acid from the monomer, changing the polymerization conditions to obtain compounds with different molar masses, is an alternative to get the oligomeric lactic (OLA), which acts as a plasticizer [53].

PLA is the most used bio-based polymer, and several processing methods are used to achieve different properties for numerous applications. The main manufacturing techniques include extrusion, cast films, injection molding, thermoforming, foaming, 3D printing, and electrospinning [54].

Melt blending approaches are the processing technologies most studied for producing PLA-based materials as they are used at the industrial level. PLA has been blended with many plasticizers (glycerol [55], citrate esters [55,56], polyethylene glycol (PEG) [55,56], OLA [55,57], vegetable oils [58], among others) to reach a flexible material, while it has also been blended with many nanoparticles to obtain PLA-based nanocomposites such as with carbon nanotubes [59], clays [60,61], nanocellulose [62], metal nanoparticles [63], etc.

Extrusion is one the most used techniques for PLA processing, typically by using conventional screws with L/D of 24–30 and compression ratios of 2–3. The polymer is heated 40–50 °C above its melting temperature due to the presence of heater bands and the friction of the molten polymer between the screw and the barrel, which permit the melting of crystalline regions [64].

Bourbigot et al. [65] prepared a PLA-based nanocomposite with multi-wall carbon nanotube (MWNT) through reactive extrusion, observing an improvement in flame retardancy. Galvez et al. [66] investigated the influence of the speed of the extrusion process for PLA plasticized with acetyl tributyl citrate (ATBC) between 10–30 wt%.

Extrusion is the step that precedes other processes, such as injection, thermoforming, or spinning.

In injection molding, the polymer is injected into a mold following extrusion. In this case, the screw not only rotates but also moves forward to introduce the molten polymer into the mold cavities. During the injection process, the mold is kept closed, and the screw is maintained in the forward position to compensate for the shrinkage of the material caused by the solidification of the material itself. The mold presents a cooling system, which regulates the cooling process. When the material is cooled down, the molded part is ejected from the mold.

To optimize PLA injection, a compression ratio of 2.5–3 and a melting temperature of around 200–205 °C are usually used. It is very important to control the shrinkage of the molded parts, which are generally brittle due to the fast physical aging caused by the low T_g_ of PLA [67].

Aliotta et al. [68] studied the effect of three nucleating agents, LAK, talc, and calcium carbonate (CaCO_3_), on the mechanical and thermal behavior of PLA samples obtained through injection molding. LAK can increase PLA crystallinity but reduce flexural modulus and Charpy impact resistance, while an opposite effect was observed with talc and calcium carbonate.

Pawlak et al. [69] fabricated a composite constituted by PLA, maleinized linseed oil (MLO) as a plasticizer, and functionalized sheep wool fiber by extrusion and then injection molding, obtaining a hydrophobic material with improved mechanical properties.

The biocompatibility of PLA allows it to be used in food packaging, such as cups, lips, blister packaging, containers, and trays that can be produced through the thermoforming process. This technique consists of first heating to soften the polymer by irradiating it with infrared radiation (IR), then compressed air is applied, and the material is pressed against the mold, which is usually an aluminum mold. Specific storage conditions are required for PLA sheets since they are very fragile at room temperature. The ultimate product should be stored at 40 °C or below relative humidity (RH) under 50%, reducing moisture absorption and achieving unwinding resistance [70].

Barletta et al. [71] studied the effect of CaCO_3_ as reinforcement of PLA and polybutylene succinate (PBS) blends obtained by thermoforming using molds specially designed for polyolefins. Wand et al. [72] prepared nanofibrillar PLA/PET through high-pressure microcellular injection molding (HPMIM) to improve PLA melt strength, crystallinity, and foaming ability.

PLA has also been used to produce thin films, usually of 0.076 mm in thickness and sheets of 0.25 mm, by cast extrusion. PLA is first extruded through a lip die, whose gap is fixed around 10% higher than the thickness required for films and sheets, then is quenched on polished chrome rollers refrigerated with circulating cooled water. PLA films are usually orientated between 2 to 10 times their original length at 60 to 80 °C, in particular 2–3 times in the machine direction stretch ratios and 2–4 times transverse stretch ratios, and at higher _D_-lactide contents, the stretch ratios can be increased. The rapid cooling of the material provides good optical properties due to the transparent appearance caused by low crystallinity degree; besides, PLA films present a high Young modulus [54,73].

Furthermore, PLA porous foam structures have been produced for biomedical scaffolds and tissue engineering, as well as for packaging and construction; the main techniques comprise extrusion foaming and foam injection molding. In the first case, the polymer and blowing agent are injected into the extruder to further emerge from the die; the pressure drop induces a thermodynamic instability causing foaming [74].

Meanwhile, foam injection molding uses supercritical nitrogen as a foaming agent; besides, it reduces the polymer melt viscosity and permits the use of lower temperatures and pressures, reducing processing costs. This represents an advantage when biodegradable polymers such as PLA are used since they are thermally sensitive and present a narrow processing temperature range. Nevertheless, the foaming of PLA with this technique needs to be highly monitored due to its low melt strength and slow crystallization kinetics [75].

In the last decade, additive manufacturing (AM) has become one of the most important techniques to develop three-dimensional (3D) objects. Among the various techniques, fused deposition modeling (FDM) was revealed to be one the most used due to its low cost and low maintenance. Furthermore, thermoplastic polymers with low melting points, like PLA, are required [76]. The FDM method presents one or two extruder head nozzles from where the melted polymeric filament is ejected onto a platform layer by layer. Then, the printed polymer solidifies to produce the final 3D object [77].

PLA is one of the most common polymers used in FDM. However, it presents some disadvantages, such as low toughness and elongation at break, low thermal stability, and a small processing window. Moreover, PLA presents low mechanical strength compared to acrylonitrile butadiene styrene (ABS) and polycarbonate (PC). For this reason, the use of PLA composites produced by the incorporation of reinforcement is one of the main approaches used to improve its mechanical properties [78].

Hong et al. [79] incorporated lignin and COOH-lignin into the PLA matrix, achieving a better adhesion between functionalized lignin and PLA and aiming to reduce the cost of printing PLA 3D filaments. Zoumaki et al. [80] covered 3D-printed PLA filament with maize starch-based conventional and nanocomposite films. Starch-based skins enhanced the mechanical properties of neat PLA in terms of strength and stiffness.

**Figure 7:**
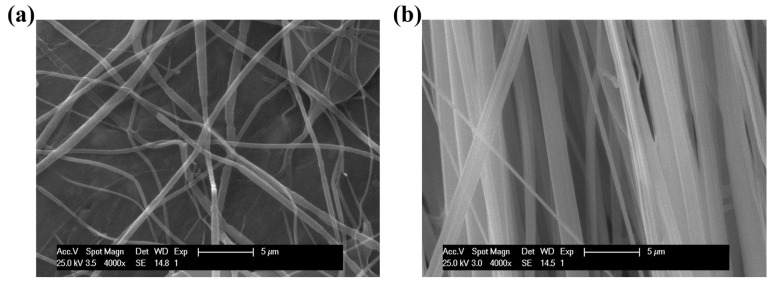
SEM images of (**a**) PLA random nanofibers and (**b**) PLA-aligned nanofibers.

Another technique to produce 3D PLA-based scaffolds is electrospinning. This versatile and low-cost technique enables the fabrication of fibers with diameters in the range of micro and nanometers through the application of a high potential field between a needle and a collector where the fibers are collected [81]. Different types of collector permit the fabrication of both random and orientated fibers (Figure 7), which present different properties such as mechanical behavior and crystallinity depending on their orientation.

In particular, nanoscale fibers present high porosity and high aspect ratio, which allows the incorporation of bioactive substances, which is ideal for biomedical applications [82]. Moreover, PLA fibers find applications in food packaging for their biodegradability and hydrophobicity, improving the water barrier properties of the package [83] and as a membrane due to the interconnectivity of pores, which is useful in the separation process [84].

Duygulu et al. [85] blended ceftriaxone disodium into PLA solution and tested different processing parameters such as flow rates and voltages. The produced electrospun nanofibers showed antimicrobial behavior against *Escherichia coli* (*E. coli*). Meanwhile, Zhu et al. [86] developed a porous membrane of PLA and HKUST-1 by electrospinning, increasing the hydrophobicity and antibacterial activity against *E. coli* and *Staphylococcus aureus (S. aureus)*, with an antibacterial rate of 99.99% with *S. aureus.*

PLA is the most produced biodegradable polymer worldwide and is highly valued in everything from packaging to biomedicine. However, its inherent rigidity and fragility limit its direct application. To mitigate this drawback, the use of plasticizers is a viable solution, as they allow the improvement of flexibility and toughness without compromising their biodegradability.

One way to improve the flexibility of PLA is the use of its own monomers: L-lactide, D-lactide, D,L-lactide monomers, and oligomeric lactic acid (OLA). In this regard, Ara and Fiori patented the process for the plasticization of lactic acid polymers, focusing on the use of oligomeric lactic acid as it is the same molecule with lower molecular weight and thus ensuring good compatibility with PLA polymeric matrix [87]. These plasticizers, used in concentrations between 10 and 25 wt%, improve ductile properties and lower the glass transition temperature. Additionally, they seem to have good storage stability for applications in contact with food [53,88,89,90].

The use of vegetable oils and their derivatives has shown broad interest, as they demonstrate good plasticizing results using low amounts, around 5 wt%, improving ductility, decreasing the glass transition temperature, and showing good compatibility with PLA. These results have been obtained with different modified vegetable oils (MVO), such as epoxidized soybean oil (ESBO) [91], epoxidized linseed oil (ELO) [92], maleinized linseed oil (MLO) [93], epoxidized jatropha oil (EJO) [94] among many others. Other molecules derived from vegetable oils that have been studied as plasticizers for PLA include glycerol, tributyrin, trilaurin, and tristearin, with tributyrin showing the best plasticizing effect, followed by glycerol with just 10 wt% [95]. To improve the plasticizing effect of glycerol, it has been combined with other molecules like succinic anhydride and alcohols for applications in flexible food packaging [96] or modified with different carbonates [97].

PLA has also been plasticized with different polymers, such as polycaprolactone [98], which significantly reduces the Tg, increasing the elongation at break and decreasing the crystallinity of the PLA. Polyamide 6 and polyamide 11 have also been used to improve the ductile properties of PLA; however, better ductile properties are achieved if they are combined with other additives, such as chain extenders [99,100]. Poly(ethylene glycol) (PEG) of different molecular weights, with lower molecular weight PEGs, for example, PEG 200 and PEG 400, being the best candidates for use as a PLA plasticizer, significantly reducing the Tg by up to 30 wt% and increasing the elongation at break [101,102,103]. Thermoplastic starch (TPS) has also been used as a plasticizer for PLA despite its immiscibility. However, TPS-rich domains lead to an improvement in the ductile properties of PLA up to 30 wt% TPS percentages [104,105]. To improve the miscibility of this system, compatibilizing agents such as MLO can be incorporated [106].

Other molecules, such as acid esters, like citric acid ester [107], cinnamic acid ester [108,109], or tartaric acid ester [110,111], among others, seem to be very interesting for use as plasticizers, in addition to incorporating other functionalities such as antioxidant agents, UV light stability, compatibilizers, or improvement of barrier properties that make them ideal for packaging applications, structural applications, or long-lasting products.

PLA can degrade in particular conditions of temperature, moisture, oxygen, and microorganisms to water, carbon dioxide, and other non-toxic products. The main degradation mechanisms include hydrolytic, enzymatic, microbial, thermal, and photodegradative mechanisms [112].

Hydrolytic degradation of PLA occurs in the presence of water and is mainly by bulk erosion, which happens when the rate of water diffusion is higher than the hydrolysis rate, and the material tends to shrink. The cleavage of the ester bond takes place initially in the amorphous regions, leading to a first increase in crystallinity. Hydrolysis leads to the formation of monomers and oligomers with hydroxyl and carboxylic acid end groups that can catalyze the hydrolysis reaction, increasing the degradation speed [113].

Vaid et al. [114] studied the hydrolytic degradation of electrospun PLA nanofibers in different conditions of pH and exposure time and compared them to molecular dynamics (MD) simulations and density functional theory (DFT) calculations. A faster degradation was found at higher pH (10) after 25 days, while slower degradation was observed at lower pH values (7.4, 2, and 3). Furthermore, they observed that PLA nanofibers degrade through a surface erosion mechanism, in contrast to the typical bulk erosion observed in PLA, caused by the increased crystallinity due to the electrospinning process.

Qu et al. [115] studied the accelerating effect of zinc oxide nanoparticles (ZnO NPs) on the hydrolytic degradation of PLA in water, with an activation energy of 38% lower than neat PLA, while Zanella et al. [116] studied the effect of the amount of silver NPs in the in vitro degradation of the nanocomposite in artificial saliva observing that the degradation time of the materials decreased by increasing the amount of NPs.

The cleavage of the ester bond also occurs through enzymatic degradation. Indeed, enzymes produced by microorganisms such as *lipases*, *esterases*, *kutinases*, and *proteases* can catalyze the hydrolysis of PLA chains in particular conditions of pH, temperature, and crystallinity of the polymer. Hegyesi et al. [117] studied the degradation of PLA with *lipase* and *proteinase K*, finding that the second one is more effective.

Donate et al. [118] fabricated composite scaffolds of PLA and CaCO_3_ and β-tricalcium phosphate (Ca_3_(PO_4_)_2_, β-TCP) in order to increase the osteoconductivity of the material and studied the enzymatic degradation by using *proteinase K* and compared with neat PLA, observing an accelerating effect in degradation caused by the presence of the additives.

Microbial degradation starts after PLA chains with high molecular weight undergo hydrolysis and smaller molecules are formed. Thus, microorganisms first release extracellular depolymerizes to further reduce the molecular weight of PLA with the presence of inducers such as gelatin, silk fibroin, and elastin. Subsequently, oligomers are absorbed by microbial membranes where intracellular enzymes convert PLA to water or CH_4_ and CO_2_ [112].

Moon et al. [119] studied the biodegradability of PLA in anaerobic digester sludge by evaluating the extent of the gas production, in particular, and the formation of PLA degradation by-product, observing an increased gas liberation as the surface area of the material increased.

Bubpachat et al. [120] isolated two bacteria from soils and wastewater capable of degrading PLA, *Stenotrophomonas pavanii* and *Pseudomonas geniculata*, respectively. These can produce *proteases*, and we studied the optimal pH for both *proteases* produced by the two types of bacteria, finding 7.5 and 8.0 as optimum values, respectively.

Microbial populations are also present in compost, which is an organic-rich, biological environment where biodegradable polymers are transformed by fungi, bacteria, and actinomycetes into H_2_O, CO_2_, inorganic compounds, and biomass in aerobic conditions. The first step involves the conversion of the polymer into small molecules, followed by a thermophilic phase where the temperature is increased due to microbial activity. Subsequently, in the cooling phase, the temperature is decreased, and humus-like substances are produced. Since plastic recycling machines are not well-equipped to recycle food-contaminated plastics, bags, and containers, compostable plastics such as PLA are a possible solution to deal with this issue [121].

Thermal degradation of PLA can be easily studied through thermogravimetric analysis. Some researchers have studied the degradation mechanism and have suggested that above 200 °C the mechanism involves different reactions, such as intra-molecular transesterification leading to the formation of oligomeric rings, which is the main degradation pathway, cis-elimination leading to acrylic acid and fragmentation producing CO_2_ and acetaldehyde, causing the reduction in molecular weight of the polymeric chains and loss of mechanical performances [122].

Furthermore, PLA is sensitive to ultraviolet (UV) and slightly sensitive to sunlight. The degradation caused by the exposition of materials to radiation is called photodegradation. When aliphatic polyesters such as PLA are exposed to UV radiation, carbon-carbon double bonds and carboxyl end groups are formed. Another mechanism suggested for the photodegradation of PLA entails photolysis of the ester bond of the main chain and the production of carboxylic acid and diketone end groups [123].

## 3. Poly(Butylene Adipate-*co*-Terephthalate) (PBAT)

**Figure 8:**
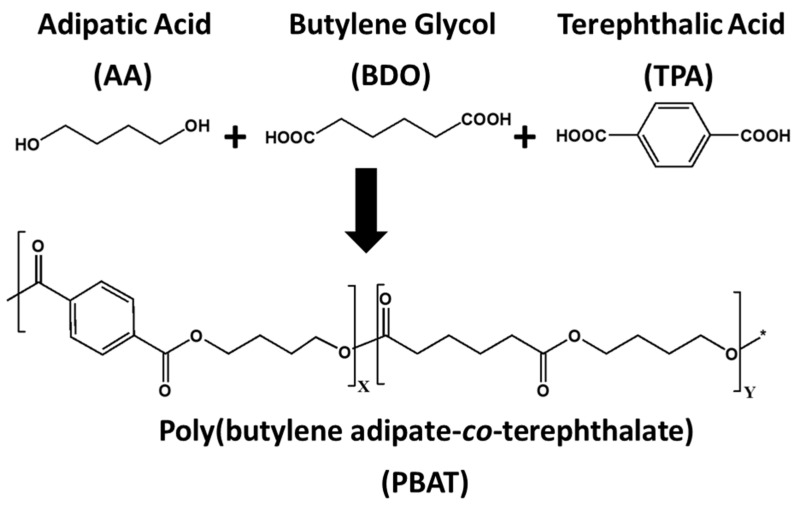
Synthesis of PBAT.

Poly(butylene adipate-*co*-terephthalate), known as PBAT, is a synthetic polymer that is biodegradable under composting conditions and, therefore, regarded as an environmentally friendly alternative to replace unbiodegradable thermoplastic films for packaging or agricultural applications [124]. PBAT combines an aromatic polyester and an aliphatic polyester, usually obtained by reacting butylene glycol (BDO), adipic acid (AA), and terephthalic acid (TPA) [125], as seen in Figure 8. In this copolymer, the aromatic monomer contributes to good thermal stability and mechanical properties, and the aliphatic monomer provides flexibility and biodegradability [126]. PBAT is more flexible than most biodegradable polyesters, including PLA and PBS, with a Young modulus of 20–35 MPa, a tensile strength of 32–36 MPa, and high elongation at break (almost 700%) [127]. The resulting high flexibility is ideal for food packaging applications and is frequently compared to low-density polyethylene (LDPE) because of its high molecular weight and branched molecular structure [128].

PBAT has been marketed under the trading name Ecoflex^®^, initially introduced by BASF towards the close of the 1990s [129], and PBAT-related copolymeric product (Biomax^®^) produced by DuPont [127]. Ecoflex^®^ is widely used to make bags, mulching films, laminated sheets, plastics for wrapping food, toothbrushes, etc. [130]. PBAT’s inferior thermal-mechanical properties compared with polyolefins and high cost (~4 USD/Kg) compared with polyethylene (PE), polypropylene (PP), and polystyrene (PS) (<1 USD/Kg) [131], encourages forming composite products in combination with other inexpensive polymeric materials. Moreover, it is worth noting that the relatively low tensile strength, melt strength, and heat resistance greatly limit its industrial applications and development [132]. Additionally, unmodified PBAT is easily broken in outdoor applications such as mulch films after a short time, and its O_2_ and CO_2_ barrier properties are also clearly lower than other common packaging materials like PET [133]. Thus, enhancing the mechanical and thermal properties of PBAT by methods such as blending, composites, and chain extension is necessary to increase its application scope.

Typically, the synthesis of PBAT is carried out in two steps: esterification, during which comparatively low-MW oligomers form, and a final polycondensation at high temperature and vacuum to get the required Mn values of about 50 kDa [134]. These conditions are necessary to eliminate the smaller, lighter molecules, like water, which can cause hydrolyzation. The esterification reaction between AA and TPA, usually in a 50:50 ratio and excess BDO, to produce the PBAT oligomer is carried out at 200–220 °C [135]. The polycondensation reaction to increase the molecular weight of PBAT polyester is carried out at 250–260 °C, with a catalyzer and low pressure, eliminating the excess BDO. A molar ratio of 50:50 (AA:TPA) produces a PBAT with good processability, optimized mechanical properties, and good compostability for packaging film applications [136]. Organometallic compounds based on zinc, tin, and titanium are normally used as catalysts during polycondensation [137]. Nucleating agents, such as talc, chalk, mica, or silicon oxides, are frequently used to enhance the crystallization ability of PBAT, improving its mechanical and physical properties [137]. Phosphoric compounds such as phosphoric acid and phosphorous acid help stabilize color in PBAT but slow down the condensation rate [125].

On an industrial scale, PBAT can be produced by batch or continuous processes utilizing traditional polyester manufacturing technologies. However, batch processing can produce partially heterogeneous reactions with various intermediate products and struggle to suppress possible hydrolysis reactions during poly-condensation, decaying the molecular weight of PBAT [138]. Therefore, a continuous process with sequential reactors is preferred, as it offers a higher control over product quality and molecular weight. Despite being mainly derived from fossil sources, PBAT is potentially obtainable from bio-derived raw materials [125]. Alternatively, PBAT can also be produced from the low molecular weight degradation product of post-consumer PET waste since a significant portion of PET’s structural backbone is TPA [139].

Wu et al. [132] studied the influence of the branching effect on PBAT using dicumyl peroxide. Generally, samples displayed higher elastic modulus and higher crystallization with increasing branching levels. Although the branching structures promoted nucleation, they reduced the diffusion rate of PBAT chain segments during the transformation of the PBAT. Moreover, melt crystallization was easier due to the higher crystallization temperature as the branching level increased.

PBAT-based films can be manufactured through multiple processing techniques, including solution casting, compression, molding, blown film extrusion, and hot pressing. Solvent casting is a simple and cost-effective method to prepare PBAT-based films, suitable for small laboratory-scale production. Chloroform is frequently the preferred solvent [140], with process conditions varying from stirring at 60 °C for 24 h and casting at room temperature for 48 h [141] to stirring at 60 °C for 1 h and drying at 70 °C for 5 h [131]. Solvent casting has also been used to prepare films by blending PBAT with other polymers, including PLA [142], PBS [143], and ternary blends such as PBAT/PLA/Babassu [126]. However, solvent casting is a less efficient method to elaborate PBAT composites when compared to other solvent-free techniques like melt processing [127].

Extrusion and blown methods are commonly used for large-scale film productions, requiring advanced equipment and large batch samples. Extrusion has several benefits, such as better control over feed, residence time and mixing degree, ease of handling high-viscosity polymers, and a wide range of processing conditions [144]. Blown film extrusion is one of the most important industrial manufacturing processes for polymeric films and is used to elaborate PBAT-based films in packaging, bags, and coverings. The process involves extruding a molten polymer into a tube shape, drawing it in both extrusion and transverse directions, and then blowing it by injecting air into a larger tube ratio [145]. The orientation of macromolecules and the final morphology of blown films depend on the chosen process parameters. PBAT/TPS (70/30) mulch films prepared by blow extrusion and compared to PE mulch films exhibited higher elongation at break and a seemingly low WVP but lower maximum tensile strength [146]. Other works have used blown processing to develop PBAT/TPS films with α-tocopherol as an antioxidant [147], PBAT/lignin films for enhanced photo-stability [148], or PBAT/TPS films loaded with polyhexamethylene guanidine hydrochloride (PHMG) for antimicrobial properties [149].

Recently, new applications for PBAT using less common PBAT processing techniques have been developed, including electrospun PBAT scaffolds with nanohydroxyapatite (nHAp) to improve bone regeneration [150], hollow PBAT electrospun microfibers loaded porous MFs (HPMFs) for controlled and sustained release of polypeptides [151], and 3D printing PBAT powder for selective laser sintering additive manufacturing [152].

**Figure 9:**
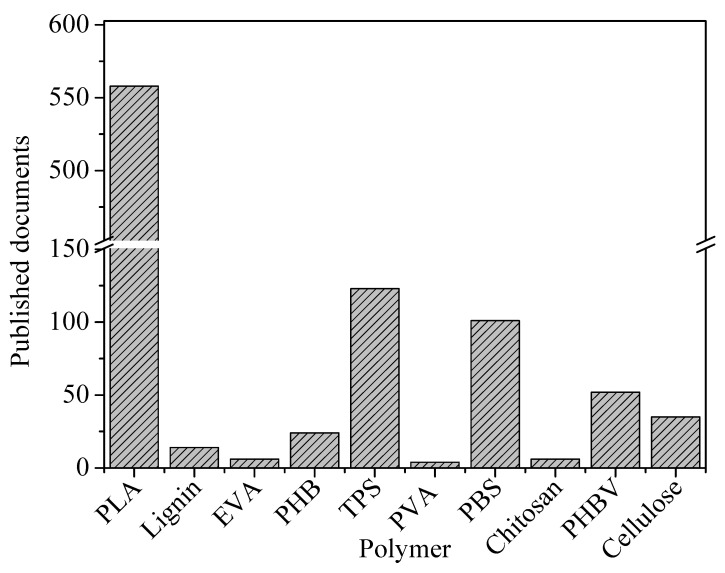
Number of publications of PBAT blends (Scopus Source up to June 2024).

PBAT is a biodegradable synthetic polymer based on fossil resources, with high elongation at break and very flexible. However, PBAT’s high cost has restricted its application; as a result, research frequently focuses on combining it with other inexpensive polymeric materials with acceptable mechanical qualities [153]. PBAT is particularly indicated for the production of flexible films blended with PLA and TPS, as seen in Figure 9, or mineral fillers; the first study in this regard dates from 2001.

Poly(lactic acid) (PLA) is a bio-based and biodegradable polyester with good processability, high transparency, and complementary mechanical properties to PBAT, exhibiting high stiffness but also high brittleness [154]. Nevertheless, because of the poor compatibility between PBAT and PLA, improving the properties of PBAT by direct melt blending these polymers can be challenging [155]. The compatibility can be improved using physical compatibilization, usually through synthesizing block/graft copolymers or reactive compatibilization strategies, such as employing dicumyl peroxide or multifunctional epoxides (ADR) [156].

Biodegradable plastic based on blends of polyester and thermoplastic starch (TPS) dominates the market of biodegradable polymers, representing more than 50% of the market share [157]. Therefore, it is unsurprising to see starch, mainly with glycerol in its thermoplastic form, has been recurrently applied as a filler for PBAT. Starch is highly regarded because of its biomass origin, high availability, quick biodegradation, affordable pricing, and low carbon footprint. However, the mechanical properties and WVP of films prepared with TPS/PBAT blends are inferior to those of conventional films, further declining as the amount of starch increases [158]. Moreover, TPS and PBAT are incompatible due to their poor interfacial affinity as a consequence of the hydrophilic nature of TPS and the relative hydrophobicity of PBAT [159]. PBAT/TPS blends require high shear forces, thus, higher screw speeds in the extrusion compounding process to obtain a homogeneous dispersion of TPS inside the PBAT matrix [135].

An effective way to improve the compatibility between TPS and PBAT is to functionalize the PBAT matrix by grafting highly reactive functions. These grafted functions can react with the hydroxyl groups of starch to form covalent bonds, providing better control of the phase size and strong interfacial adhesion [160]. Alternatively, compatibilizers including citric, maleic, and tartaric acids [159], soybean oil [158], or sodium hypophosphite as a co-agent with citric acid [161], reduce the interfacial tension caused by the difference in polarity between PBAT and TPS. A method to address the poor mechanical and water sensitivity of the TPS phase without sacrificing biodegradability is to reinforce it with the addition of nanofillers. Multiple nanofillers have been proposed in PBAT/TPS blends, including halloysite clay nanotubes (HNTs) [162], needle-like sepiolite [163], bentonite (BT) [164], and cellulose nanofibrils (CNFs) [157].

Nanocomposites offer an attractive method to improve pristine PBAT properties. A wide range of nanomaterials have been added as reinforcements or additives to PBAT films derived from natural organic sources, such as acetylated cellulose nanocrystals (CNFs) [131] and CNFs modified with octadecyl isocyanate [165] and inorganic resources such ZnO [141], TiO_2_ [166] or Ag [140] nanoparticles, and layered silicate nanoclays including MMT [167], octadecylamine-modified MMT [168] or MMT/Cloisite/Bentonite [169].

A comparison between PBAT/MMT and PBAT/CTAB-modified MMT (CMMT) nanocomposites was carried out by Mondal et al. [170]. PBAT/CMMT showed greater property enhancement, decreasing WVP, and stronger antimicrobial activity, especially against gram-positive bacteria. However, PBAT/CMMT displayed less biodegradation after 100 days. Han and Park [171] proposed the elaboration of a PBAT/PE blend to improve the mechanical properties of PBAT mulching films via blown film extrusion and extend its decomposition period. The blend was compounded with a PE mixture (LDPE:LLDPE = 15:85) and added peroxide di-(2-tert-butly-peroxyisopropyl) benzene (BIBP) as a crosslinking agent. The compatibilization effect of BIBP increased the storage modulus of the blend, provided higher bubble stability during extrusion, and improved the film’s surface morphology.

The degradation of PBAT waste can be divided into abiotic degradation (physical and chemical degradation) and biotic degradation. Biodegradability in composting conditions and soil burial conditions is affected by both biotic and abiotic factors of the environment, such as temperature, moisture, pH, bio-surfactant, and enzymes, and by polymer characteristics, such as chain flexibility, crystallinity, regularity and heterogeneity, functional groups, and Mw [172]. Biotic degradation is a slow process that uses microorganisms or other organisms to degrade PBAT into carbon dioxide and water. In contrast, the fast abiotic process degrades PBAT into smaller molecules. Moreover, abiotic methods preferentially target the chemical bonds with higher energy (especially C-O and C=O) of PBAT, eventually shortening the polymer chain and then reducing the molecular weight [173].

PBAT can theoretically be disposed directly into the soil without recycling, entirely decomposing into water, carbon dioxide, and microbial biomass [174]. Nevertheless, PBAT has a very low biodegradation rate compared to those of other biodegradable polymers such as PHAs and PCL, caused by the high resistance to enzymes to the TPA ester bonds and the small number of PBAT-degraders in the environment [175]. Therefore, the biodegradation rate is highly influenced by the aromatic segment of TPA, with faster biodegradation occurring with decreased TPA content in the structural backbone [172]. Ma et al. [176] explained how biodegraded PBAT films in soil exhibited an increase of O elements and a decrease of C elements in XPS, indicating that the degradation process involved the oxidation of molecular chains. In addition, the hydrolysis of ester bonds in PBAT films led to the breakdown of molecular chains and subsequent degradation into smaller molecules.

The biodegradation rate under mesophilic (37 °C) and thermophilic (55 °C) anaerobic conditions was studied by Svoboda et al. [177] by measuring biogas production. Under mesophilic conditions, the biodegradation after 126 days was only 2.2%, Mw had changed from 93,000 to 25,500 g/mol, and crystallization had experienced slight changes. On the other hand, under thermophilic conditions, biodegradation reached 8.3% after 126 days, Mw decreased from 93,000 to 9430 g/mol, and the crystallization behavior changed significantly.

Nomadolo et al. [178] compared the biodegradation of PBAT/PLA and PBAT/PBS blend films under industrial and home composting conditions. PBAT-PBS reached 82% and PBAT-PLA 90% mineralization in 120 days in industrial conditions, and under home composting for 200 days, samples reached 72% and 50% degradation, respectively. Dammack et al. [179] examined the use of maleated poly(butylene adipate-*co*-terephthalate) (PBATg-MA) and maleic anhydride (MA) as compatibilizers in PBAT/TPS blends and their effect on biodegradation. In the presence of MA, the degradation profile of the blends was close to that of neat PBAT and TPS. On the contrary, in the presence of PBATg-MA, biodegradation of the blends was 70–75% after 80 days, while it exceeded more than 85% without any compatibilizer. The authors explained that PBATg-MA might have created a chemical continuity between the PBAT and the TPS phase, generating an interfacial domain that is harder to degrade.

The photodegradation and thermophile biodegradation of two commercial mulching films based on PBAT and a PBAT/PLA blend were studied by Morro et al. [180]. Photodegradation was efficient in both films, reducing the Mw by 16% for PBAT and 31% in the blended film. The photodegraded samples then presented higher susceptibility to biodegradation as the reduction in molecular weights facilitated bacterial action. On a similar note, Zhang et al. [181] examined the effect of light stabilizers on the weathering resistance of PBAT/PLA blend films. The addition of a light stabilizer prevented the chain scission of polyesters by absorbing damaging UV, preventing a significant decrease in Mw.

Yang et al. [182] employed *Thermobifida fusca* cutinases, a type of enzyme, to break PBAT films into fragments in 4 h, with further digestion into small particles in 12 h and a complete decomposition in 48 h. Another alternative approach was proposed by Pang et al. [183], employing a 1,4-butanediol-alkali combined method for the hydrolysis of PBAT. Subsequently, a two-step synthesis produces a foam nickel-supported NiOOH catalyst for the electrocatalytic reformation of the PBAT hydrolysate into succinate salts, a higher-value chemical.

## 4. Polyhydroxyalkanoates

Polyhydroxyalkanoates (PHAs) are a natural, biodegradable, and biocompatible family of aliphatic polyesters that find applications in food packaging and the biomedical field [184].

This biopolymer is produced by more than 300 bacteria, both Gram-positive and Gram-negative, under particular conditions [185].

**Figure 10:**
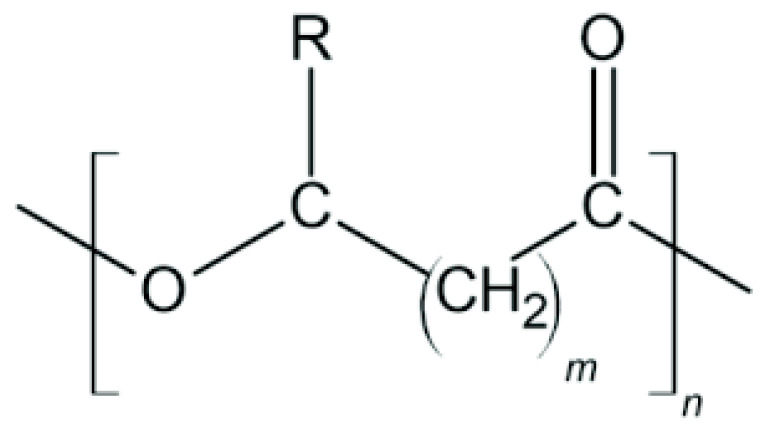
PHAs general structure.

The general structure of PHAs is reported in Figure 10.

The –R group can be a saturated, unsaturated, or branched alkyl group, and according to the chain length, can be divided into three categories [186,187]: (i) short chain length PHA *(scl-PHA)* that consists of monomers of 3–5 carbons and present a high degree of crystallinity, (ii) medium chain length PHA *(mcl-PHA)* with monomers of 6–14 carbons that can be aliphatic or aromatic. They present a low degree of crystallinity and are elastomeric. (iii) long chain length PHA with 15 or more carbons in the building block.

PHAs are generally UV stable and present a variable degree of crystallinity. Indeed, they could be completely amorphous or reach a crystallinity of 70%, depending on the monomeric building block [188].

Their mechanical properties are similar to those of polypropylene (PP) and polyethylene (PE) but more brittle and with lower elongation at break. However, the mechanical properties depend on the length of the polymeric chain and the crystallinity of the material [189]. In general, PHAs present a Young modulus between 0.008 and 3.5 GPa and tensile strength between 6.6 and 50 MPa [190].

Concerning the thermal properties, T_m_ decreases by increasing the side chain of the carbon length while T_g_ increases. Indeed, T_m_ can vary from 53 to 180 °C and T_g_ from 1 to 50 °C [191].

Due to their biodegradability and barrier properties, PHAs find applications in particular in industrial applications such as food packaging [192]. Genovesi et al. [193] prepared blends of poly(3-hydroxybutyrate-co-3-hydroxyhexanoate) (PHBH) and poly(3-hydroxybutyrate-4-hydroxybutyrate) P(3HB)(4HB) through reactive extrusion obtaining a film suitable as packaging material for vegetables and fruits, with increased applications.

Their biocompatibility and biodegradability allow the use of these kinds of polymers for biomedical applications such as medical devices and implants [194].

Ye et al. [195] developed a material for bone tissue engineering by using PHA as a biodegradable polymeric matrix reinforced with β-tricalcium phosphate (β-TCP) at different percentages from 0 to 20 wt% to improve its mechanical properties and osteoconductivity and finding that this scaffold can promote the proliferation, adhesion, and migration MC3T3-E1.

PHAs have many advantages, but their poor mechanical properties and low thermal stability represent a shortcoming for their processing and applications, which are overcome with the production of blends or nanocomposites [196].

Polyhydroxybutyrate (PHB) belongs to the family of PHAs and is characterized by a –CH_3_ group in the side chain, which makes it highly crystalline, thermoplastic, and hydrophobic [197].

Bio-based synthesized PHB presents an isotactic structure with the carbons in the R(-)-configuration. Indeed, only this configuration can be recognized by microorganisms and degraded, while synthetic PHB presents more irregularities and cannot be degraded to a high extent [198].

The high crystallinity (around 70%) is the main reason for its outstanding mechanical properties, with an elastic modulus around 2.5–3 GPa and tensile strength of 25–40 MPa, comparable with PP and PE [199].

PHB, which presents less permeability than PE and PP [200], finds application in food packaging as a replacement for common PP-based packaging [201]. Furthermore, as bio-based and biodegradable polymer it is widely used in the biomedical field [202], tissue regeneration [203], and drug delivery [204].

However, PHB presents low thermal stability, and the narrow range between its Tm and Td, which is close to 180 °C, represents a limitation in the processability of this polymer [205].

**Figure 11:**
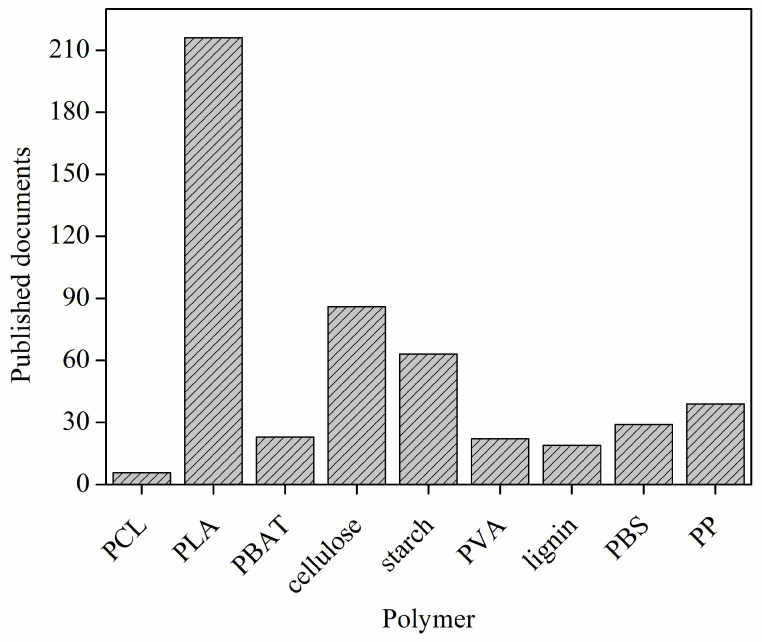
Number of publications of PHB blends (Scopus Source up to June 2024).

In order to overcome these problems, PHB was blended with various natural and synthetic polymers since 1998, as reported in Figure 11 [206]. The addition of reinforcement as fibers or both organic and inorganic nanoparticles also permits the improvement of mechanical and conductive properties and increases the thermal stability of PHB [207].

Poly(3-hydroxybutyrate-co-3-hydroxyvalerate), PHBV, is obtained by the copolymerization of 3-hydroxybutanoic acid and 3-hydroxypentanoic acid or 3-hydroxyvalerate (HV) and can be produced by both gram-positive and gram-negative bacteria, as *Ralstonia* or *Alcaligenes eutrophus* [208].

The physical and mechanical properties of PHBV depend on the 3HV content; indeed, the crystallinity and melting point decrease by increasing 3HV content while the degradation rate increases [209].

Since the presence of HV leads to the formation of a less crystalline structure, the reduced chain packing increases flexibility and toughness with respect to PHB [210].

**Figure 12:**
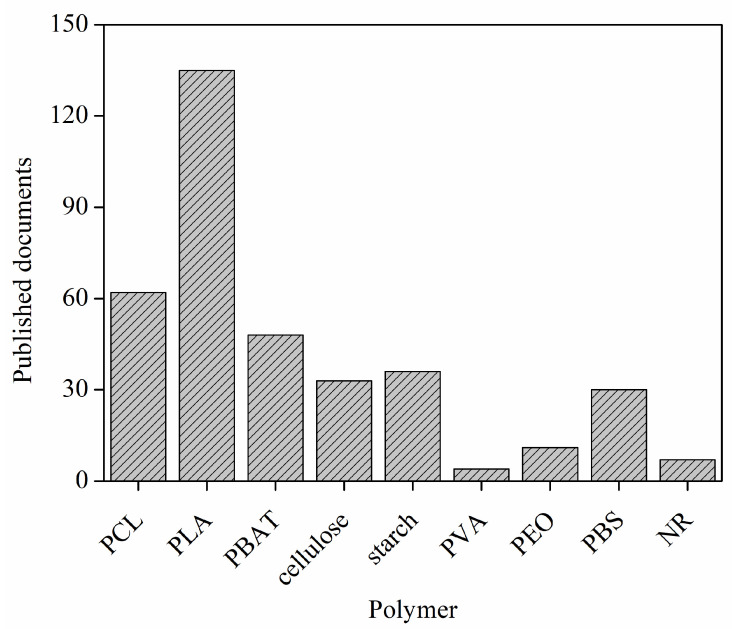
Number of publications of PHBV blends (Scopus Source up to June 2024).

Besides its biodegradability and biocompatibility, it presents some shortcomings, such as hydrophobicity, poor mechanical properties, and low thermal stability, which can be improved by fabricating nanocomposites with natural fibers, clays, silicates, etc. [211]. Moreover, blending PHBV with other polymers such as PCL, PLA, PBS, or cellulose and starch, as shown in Figure 12, permits the overcoming of these issues [212].

− Natural biosynthesis

PHAs are usually produced through the induction of enzymatic reactions in microorganisms from diverse feedstocks [213].

When there is an excess in carbon source and a lack of nutrients such as nitrogen, sulfur, and phosphates, bacteria start to accumulate PHA, stored as granules in the cytoplasm, which undergo depolymerization and are metabolized by bacteria when the external carbon source is consumed [187].

In general, the biosynthesis of PHAs can be summarized in three different pathways: direct synthesis pathway, β-Oxidation pathway, and fatty acid synthesis pathway [214].

− Direct synthesis pathway

The first route, which is more typical for the synthesis of scl-PHA, is carried out by *Ralstonia eutropha* and *Azotobacter beijerinckii*. The synthesis involves the conversion of Acetyl-CoA to Acetoacetyl-CoA, which is subsequently transformed into (R)-3-Hydroxyacyl-CoA, catalyzed by *PhaA* and *PhaB* respectively. Finally, (R)-3-Hydroxyacyl-CoA is converted into scl-PHA [215].

− β-Oxidation pathway

This pathway is one of the main routes for the synthesis of mcl-PHA, which occurs in *Pseudomonas* strains by using fatty acids [216].

Acyl-CoA is used as substrate and is converted into Enoyl-CoA, which is subsequently transformed into (R)-3-hydroxyoxycil-CoA catalyzed by (R)-specific-enoyl-CoA hydrase. Lastly, (R)-3-hydroxyoxycil-CoA is used as a substrate by PHA *synthase* [217].

− Fatty acid synthesis pathway

mcl-PHA can be produced either through the β-Oxidation pathway or through the fatty acid synthesis pathway, both using fatty acids as starting material [218].

In this route, fatty acid synthesis leads to the production of 3-ketoacyl-ACP, then transformed into (R)-3-hydroxyacyl-ACP. The enzyme *PhaG* catalyzes the transformation of (R)-3-hydroxyacyl-ACP into (R)-3-Hydroxyacyl-CoA, which is finally converted into mcl-PHA [219].

− Bioengineering strategies

As a result of the advancement of bioengineering, new methods have been developed in order to increase the bio-catalytic activity of PHA synthase, the utilization of new HA monomers, and the genetic modifications of bacteria [220,221].

Thomas et al. [222] carried out the characterization of *Halomonas* sp. SF2003, which is a marine bacterium able to produce PHA by using two genes identified as PhaC1 and PhaC2, and the study of potential carbon substrates for PHA production and the production itself. Furthermore, PhaC1 and PhaC2 *synthases* were expressed in *Cupriavidus necator* (*C. necator*) H16 PHB¯4 (DSM 541). *Halomonas* sp. SF2003 and *C. necator* showed preferably glucose and fructose as substrates, respectively. 

Cha et al. [223] produced four types of scl-PHA through the modification of the levulinic acid (LA) metabolic pathway in *Pseudomonas putida* EM42 and expressing different types of PHA *synthase* to produce PHA with different monomer fractions, reaching 40% of cell dry mass under non-optimized flask culture.

Another method used for PHA synthesis is the use of waste-activated sludge (WAS), which also permits a sustainable treatment of wastewater and represents a source of mixed microbial culture (MMC). The process entails a first enrichment of *bacteria* in a sequencing batch reactor (SBR) and a second accumulation of PHA in *bacteria* through the aerobic dynamic feeding method (ADF), where *bacteria* are submitted to alternating feat and famine periods [224]. PHA accumulation has reached 90% gPHA/gVSS on a laboratory scale and a value between 40 and 80% gPHA/gVSS on an industrial scale [225].

Estévez-Alonso et al. [226] carried out the production of PHA through activated sludge from municipal wastewater treatment and demonstrated that the addition of calcium stimulates the selective enrichment of PHA-accumulating bacteria and improves PHA production three times more compared to control without calcium after 48 h.

Zhang et al. [227] investigated the production of PHA using volatile fatty acids (VFAs) from acetate to valerate in SBRs inoculated with activated sludge in ADF at nitrogen-limiting conditions with high yields.

As with all types of PHAs, PHB as well is synthesized by microalgae and in bacterial cells in carbon-excess and critical environment conditions as limited nutrients [187].

Vroman individuated three main routes to synthesize PHB [197]. ROP of β-butyrolactone (BL) is one of them [228]. Yang et al. [229] carried out the synthesis of PHB through ROP by using different bifunctional organoboron catalysts in order to avoid the use of metallic catalysts and obtain a polymer selectivity above 99% with the optimized catalyst. Jian et al. [230] used Zirconium compounds as initiators and obtained a PHB with an average molecular weight of 12,000 g/mol.

The utilization of natural/transgenic plants, which contain acetyl-CoA that is used as a substrate, is another approach [231,232]. Lössl et al. [233] used transplastomic tobacco to obtain PHB, and Parveez et al. [234] realized PHB biosynthesis in oil palm by using three bacterial genes, *bktB*, *phaB*, and *phaC*.

The third and more common method is through bacterial fermentation [235]. Acetyl-CoA is the main substrate; two molecules are condensed by *PhaA* to obtain acetoacetyl-CoA, which is subsequently reduced by *PhaB* to 3-hydroxybutyryl-CoA. Lastly, *PhaC* catalyzes the polymerization of (R)-3-hydroxybutyryl-CoA [236].

El-Kadi et al. [237] produced PHB using three different carbon sources: dried whey, sugar beet molasses, and date molasses. Thirty isolated bacteria were tested, and *Bacillus paramycoides* MCCC 1A04098, *Azotobacter salinestris* NBRC 102611, and *Brevundimonas naejangsanensis* BIO-TAS2-2 were identified as the best bacteria to obtain PHB.

*C. necator* is widely used for PHB production. Lambauer et al. [238] carried out first the cultivation of *C. necator* H16 in the presence of an explosive gas mixture, H_2_, O_2_, and CO_2_ under O_2_ in a bioreactor; 98% of PHB was obtained from CO_2_. However, in order to increase PHB production, metabolic engineering is used to modify the native metabolic pathways of this bacterium [239].

The biosynthesis of PHB in bacteria involves two parallel pathways, one for the production of (3-hydroxybutyrate) monomer and the other for the production of (3-hydro- xyvalerate) monomer [210].

In the first step, two molecules of acetyl-CoA, derived from glycolysis and tricarboxylic acid (TCA) cycle, are condensed by *phaA* into acetoacetyl-CoA, which is further converted into 3-hydroxybutyryl-CoA by *phaB*. Other substrates, such as propionic acid, which is the precursor of 3HV, are metabolized to obtain propionyl-CoA that subsequently reacts with acetyl-CoA to form 3-ketovaleryl-CoA. *PhaB* catalyzes the reduction of 3-ketovaleryl-CoA to 3-hydroxyvaleryl-CoA. Finally, the polymerization of the two monomers is catalyzed by *phaC* [240].

**Figure 13:**
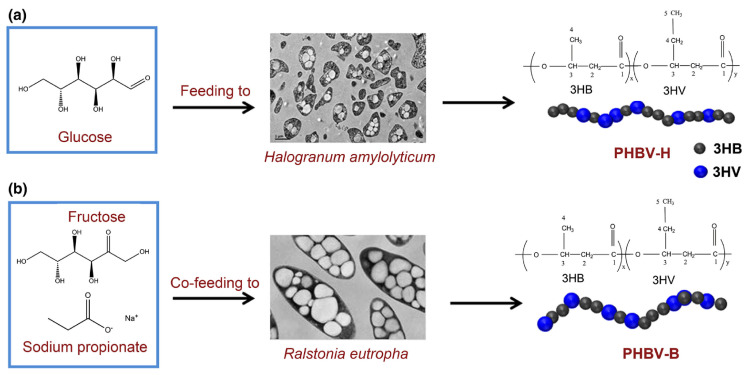
Schematic representation of PHBV production (**a**) in *H. amylolyticum* and (**b**) *R. eutropha* [241].

There are different bacteria able to produce PHBV; Zhao et al. [241] studied the production of PHBV in *Halogranum amylolyticum* (PHBV-H) and *Ralstonia eutropha* (PHBV-B) by using different carbon sources (Figure 13).

The two copolymers presented similar content of 3HV monomer, almost 21%, but exhibited different mechanical and thermal properties, crystallinity, surface energy, and wettability.

Meng et al. [242] carried out the production of PHBV in *E. coli* by expressing two genes, namely *phaCp* and *phaABp*, cloned from *Propylenella binzhouense* L72T. The highest yield (1.06 g/L) was obtained when glucose and propionic acid were used as substrates. The obtained PHBV showed improved thermal stability (298 °C) and high crystallinity.

Recently, few works have been focused on the reutilization of waste as carbon sources. Indeed, Altun et al. [243] generated PHBV from waste cooking oil in a lab-scale bioreactor and reinforced it with pistachio shell flour to fabricate a biocomposite through melt mixing. Suhazsini et al. [244] obtained PHBV from *Bacillus megaterium* of cheese whey permeate by co-feeding propionic acid used as a plasticizer. Parameters such as time, C/N ratio, and propionic acid content were optimized. Indeed, they obtained the highest yield (3.64 g/L) after 72 h, 0.1% of propionic acid, and a C/N ratio of 15.

Furthermore, PHBV production was also studied in activated sludge. Chen et al. [245] used primary sludge (PS) and waste-activated sludge (WAS) as carbon sources, achieving 65.6% of the content, and identified *Thauera* as the predominant strain.

However, fed-batch fermentation, which consists of the continuous addition of substrates to microorganisms and incessant removal of products, is the most used method to obtain a higher amount of PHBV [240].

Chang et al. [246] utilized genetically modified *Methylorubrum extorquens* AM1 to over-express PHBV. Fed-batch fermentation implemented with multiple cycles was used to remove the excess of accumulated sodium, obtaining 2.76 g/L of copolymer.

Hathi et al. [247] reutilized food waste from restaurants to produce PHBV, using *C. necator* as a PHBV-producing strain through a fed-batch fermentation strategy. They obtained 5.32 g/L of PHBV with a 50% molar 3HV fraction.

The recovery process, which involves the separation of PHA from biomass after its production, is the first step before the processing of PHA. Several approaches have been adopted in order to extract polymeric chains from cells; the downstream processing depends on various factors such as microbial strain, type of PHA, and content [248].

The typical processing techniques adopted for polymer manufacturing are used for PHAs as well, as solvent casting, fiber spinning, injection molding and film extrusion, phase separation, freeze drying, foaming, 3D printing, and the synthesis of NPs [249,250].

Mármol et al. [251] produced films of PHA by solvent casting, reinforced with graphene nanoplatelets (GNPs) at different concentrations and studied the mechanical, electrical, and piezoelectric response of this material that found potential applications as sensors.

Chen et al. [252] fabricated PHA/polyvinyl alcohol (PHA/PVA) membranes by combining electrospinning and spin-coating methods. The membrane is used as a drug delivery system for levofloxacin (LEV), which was added at 0.05 and 0.1 wt% and showed antibacterial activity against *E. coli* and *S. aureus.*

Liu et al. [253] integrated graphene-decorated gold NPs (RGO/Au) into poly-3 hydroxybutyrate-co-12 mol.% (P3HB-co-12 mol.%) hydroxyhexanoate (HHx) fibers through electrospinning. They produced a scaffold suitable for nerve tissue regeneration by combining the conductivity of graphene with nanofibers. Indeed, the electroactive scaffold promoted Schwann cell (SC) proliferation and migration.

Ivorra-Martinez et al. [254] reinforced poly(3-hydroxybutyrate-co-3-hydroxyhexanoate) [P(3HB-co-3HHx)] with hydroxyapatite nanoparticles (nHA) in a range between 2.5 and 20 wt%. The nanocomposite produced through injection molding exhibited improved mechanical properties, in particular with concentrations above 10 wt% of NPs, which presented ductility and strength similar to native bone.

PHA has also been found to be applicable in soft tissue engineering. Chen et al. [255] developed a scaffold via a phase separation method based on 3-hydroxybutyrate (3HB) and 4-hydroxybutyrate (4HB) (P34HB) (or PAP34HB) reinforced with ZnO NPs for oral soft tissue regeneration and antibacterial activity against bacteria as *Porphyromonas gingivalis* and *Streptococcus mutans.*

Thermally induced phase separation followed by freeze drying was used by Zeinali et al. [256] to produce a 3D microporous scaffold of poly(hydroxybutyrate-co-hydroxyvalerate) (PHBV) copolymers. The scaffold promotes cell adhesion and proliferation, which makes this scaffold suitable for tissue regeneration.

Lopresti et al. [257] blended PLLA with PHA at 30 wt% in order to produce a foam suitable as a scaffold for tissue engineering. Blending PLLA with PHA improved the wettability, biocompatibility, and proliferation capacity of the scaffold.

Zhang et al. [258] developed a 3D-printed scaffold based on poly(3-hydroxybutyrate-co-4-hydroxybutyrate) (P34HB) by fused deposition modeling (FDM). PHA scaffold was integrated with bone morphogenetic protein 2 (*BMP2*) and immobilized through a polydopamine (PDA) coating, improving its osteogenic bioactivity and cell adhesion and proliferation.

As a thermoplastic polymer, PHB can be processed with typical processing techniques such as film casting and blowing, injection molding, electrospinning, extrusion, thermoforming, etc. [259].

However, it is extremely brittle and presents a narrow processability range of temperature due to its T_d_ close to its T_m_ (180 °C), limiting the residence time of some techniques to a few minutes [260]. To overcome this issue, Uzun et al. [261] fabricated different composites of PHB and three different bio-fillers as lignin, alpha-cellulose, and cellulose nanofibrils by solvent casting, observing an increased thermal performance in terms of T_c_ and T_m_, particularly when lignin was added.

In order to expand PHA application fields, Anbukarasu et al. [262] developed a nanocomposite constituted by PHB and GNP through solvent casting, suitable as an actuator or sensor. The resistivity was studied at different casting temperatures, and observing a decrease in resistivity by increasing the casting temperature, from 42.3 Ω cm for 80 °C to 3.01 Ω cm for 110 °C and 1.5 Ω cm for 140 °C. Furthermore, the resistivity decreases with the physical aging due to an increase in the crystallinity and density of the material, resulting in a smaller separation of GNP reinforcement, improving the mobility of electrons between the nanoplatelets.

With the aim of replacing petroleum-based plastics with biodegradable polymers, Hernandez et al. [263] added microspheres, carbon fibers, or polyethylene glycol (PEG) of different MW: 2000, 10,000, and 20,000 through injection molding. They studied the influence of the reinforcement in thermal stability, observing a slight increase only when PEG at higher MW was added; however, all the samples presented a reduction in hardness and compressibility.

Bucci et al. [264] fabricated material for food packaging through injection molding; PHB material was compared with PP-based packaging in terms of dimensional and mechanical properties. PHB resulted in a more rigid material with respect to PP, showing a lower deformation of about 50%.

PHB-based material for packaging applications was studied also by Olejnik et al. [265] by blending PHB with PLA at different mass ratios, 100/0, 50/10, 50/20, 40/30, 50/50, 30/40, 20/50, 10/50 and 0/100 mass ratio through extrusion, identifying PLA/PHB 50/10 the blend with the optimal properties.

Weinmann et al. [198] realized a chemical modification of PHB by reactive extrusion in order to get either branched or crosslinked material with improved thermal and mechanical properties, also with the addition of rapeseed oil and PEG 1500 and PEG 4000 as plasticizers. The addition of plasticizers increased the melt stability and melt viscosity. Furthermore, the materials presented higher thermal stability by retarding degradation reactions.

Electrospinning is another technique used for PHB manufacturing. Arrieta et al. [266] fabricated PLA-PHB nanofibers in different proportions (100:0; 75:25; 50:50, 25:75, and 0:100) and studied their thermal, mechanical, and morphological properties. The proportion PLA-PHB 75:25 showed the highest interactions between the two polymers, and PHB reduced the presence of beads. Furthermore, they added acetyl tri-n-butyl citrate (ATBC) as a plasticizer to the best formulation, obtaining a more flexible and ductile material with improved thermal stability.

Kundrat et al. [267] fabricated PHB meshes for drug delivery applications. Levofloxacin was incorporated into the nanofibers depending on the viscosity of the PHB solution and morphology of nanofibers in a range of 14.4–81.8%. The sponge model incorporated the highest amount of levofloxacin, while nanofibers with and without beads entrapped 22.3% and 14.4%, respectively. Moreover, drug release was studied in PBS, and all samples were able to release between 30.4 and 32.5% of the incorporated drug.

PHB has also been processed by thermoforming. Sánchez-Safont et al. [268] produced thermoformed trays of PHB, highly purified commercial cellulose fibers (TC90) were used as filler and hexamethylene diisocyanate (HMDI) as compatibilizer. Wei et al. [269] obtained a cross-linked PHB with dicumyl peroxide (DCP) (0.25–1 wt%), obtaining an improvement in melt strength, which is an important property for processing techniques such as thermoforming.

The common processing techniques used for PHB can be used to process PHBV as well. PHBV presents higher flexibility with respect to PHB, and the presence of HV expands the processing range of temperatures with higher melt stability [270].

Erceg et al. [271] fabricated films based on PHBV and PCL-diol by solvent casting, obtaining the optimal composition in terms of film morphology with 60 wt% of PCL.

Perez-Martinez et al. [272] fabricated PHBV/PLA sheets for food packaging in order to replace PP, which is commonly used in this field. The films were developed by solvent casting and extrusion in different proportions: 50/50, 60/40, 80/20, and 100/0 (%*w*/*w*). PHBV presents exceptional barrier properties, while PLA improves the thermal stability of the material. The extruded films showed better mechanical properties, and the formulation PHBV/PLA 50/50 presented properties similar to those of PP.

With the same purpose, Samaniego-Aguilar et al. [273] obtained a binary blend of PHBV and thermoplastic polyurethane (TPU) by reactive extrusion and using hexamethylene diisocyanate (HDMI) as a reactive agent and compatibilizer. The material presented improved ductility and impact toughness, due in particular to the presence of HDMI.

Frącz et al. [274] developed PHBV-based reinforced with 30% of three types of fibers (1 mm length): flax, hemp, and wood. The material was processed first by extrusion and then by injection molding. Only hemp and flax fibers improved the mechanical properties of neat PHBV.

Mallegni et al. [275] reutilized agro-food residue as coffee silverskin (CS) to produce a PHBV/CS biocomposite, utilizing ATBC as a plasticizer and CaCO_3_ as reinforcement. Furthermore, they produced coffee capsule bodies through injection molding and studied the migration properties of the biocomposite with simulants at 100 °C, which was below the limit (10 mg/dm^2^) needed for materials apt for food-packaging applications.

Electrospinning is a versatile technique used to produce nanofibers, and PHBV electrospun nanofibers find applications in the biomedical field and industrial applications [276].

Rabbani et al. [277] fabricated PHBV beaded nanofibers and beadless microfibers. In order to increase the porosity and roughness of nanofibers, polydimethylsiloxane (PDMS) treated silica was added to the solution and subsequently treated with stearic acid at 80 °C. The incorporation of NPs and stearic acid decreases the crystallinity of the material. Furthermore, they obtained a superhydrophobic material with high static contact angle WCAs ≥ 155° and sliding angles ≤ 5° suitable for biomedical applications but also water filtration, oil-water separation, and food packaging.

Karbowniczek et al. [278] developed PHBV-based electrospun fibers reinforced with HA for bone tissue engineering. The hybrid material improved filopodia formation, cell proliferation, and anchoring within the scaffold.

Another system widely used for the production of scaffolds for tissue engineering is 3D printing. Kontogianni et al. [279] blended PHBV with PLA and PCL in a ratio PLLA/PCL/PHBV 90/5/5 wt% through twin-screw extrusion. Furthermore, they added nano-HA and strontium-substituted nano-HA (Sr-nano-HA) to produce the filament to be printed by FDM. The presence of reinforcement and the porous structure of the scaffold facilitated cell attachment and proliferation.

de Carvalho et al. [280] used the same technique to fabricate a filament of PHBV reinforced with ZrO_2_·nH_2_O particles between 1 and 10% wt/wt. The presence of particles decreases the crystallinity of PHBV and increases the relative density.

There are numerous studies on the compatibilizing effect of modified oils in blends of immiscible polymers. They observed how the separate incorporation of epoxidized soybean oil (ESO), acrylate soybean oil (AESO), and maleinized soybean oil (MSO) improved compatibility in immiscible blends of PLA/PHB/PCL (60/10/30), obtaining formulations with improved ductile properties in all cases [281]. Other blends with different percentages of PLA (75%)—PHB (25%) obtained an interesting result after the incorporation of modified oils, epoxidized (ECO) and maleinized (MCO) corn oil showed an effect on the mechanical properties, obtaining an increase on ductility for both plasticizer, thermal properties were slightly reduced, but the incorporation does not affect the thermal stability of initial polymers. Also, the degradability of samples showed an increase in the degradability grade compared with the initial blend, PLA—PHB (Sempere-Torregrosa et al., 2022 [282]).

Adding the OLA plasticizer to a blend of PLA (85%) and PHB (15%) reduces tensile strength but increases ductility, indicating a more flexible material and a decrease in Tg was observed. Although other thermal properties decrease, thermal stability remains stable up to 20% by weight of OLA. With 30% OLA, degradation intensifies. Also, water vapor and oxygen permeability increase, but crystallinity and hydrophobic behavior are suitable for food packaging applications [88].

The incorporation of a polyester plasticizer (Lapol 108) at two different concentrations, 5 and 7% by weight per 100 parts of the blend PLA/PHB, was studied. Thermal values show only one Tg value, indicating polymers of the blend are miscible. The rest of the results showed that the plasticizer does not affect the thermal stability of PLA and PHB. Also, the addition of plasticizer improved ductile properties, obtaining an increase of elongation at break values [283].

PHAs are biodegradable polymers obtained through biosynthesis and biopolymerization by microorganisms. The most studied and employed ones are poly(3-hydroxybutyrate) (PHB) and the copolymer poly(3-hydroxybutyrate-co-3-hydroxyvalerate) P(3HB-co-3HV) or PHBV. Additionally, their mechanical and thermal properties are like those of common polymers obtained from fossil sources. Although their properties vary considerably depending on their structure, they are generally characterized by a high crystallinity of up to 80%, exhibiting high rigidity and brittleness, as well as low thermal stability. These properties can be modified by the incorporation of plasticizers, resulting in significant variation.

The plasticizers used to modify the properties of PHAs are based on the use of vegetable oils and their derivatives, such as glycerol, citrates, and glycol esters. Seoane et al. studied the effect of epoxidized soybean oil (ESBO) on the properties of PHB. The results demonstrated how the incorporation of 5% by weight of oil increased the elongation at the break of PHB by 45% and slightly reduced its tensile strength and elastic modulus. As for thermal properties, the incorporation of 5% by weight of ESO slightly increased the onset temperature of PHB degradation without affecting the melting temperature, thus maintaining its thermal stability [284]. The effect of vegetable oils such as epoxidized linseed oil and ESBO have also been studied as compatibilizers of PHBV, both showing good plasticizing properties incorporating 10 wt% since they reduce tensile strength and elastic modulus and increase elongation at break. In addition, a decrease of between 4 and 5 °C in the melting temperature was also observed [285].

Glycerol (G) and its derivatives, such as glycerol tributyrate (GTB), glycerol triacetate (GTA), and polyglycerols (PGs), have also been studied as plasticizers for PHB. Among them, only the additives GTB and GTA, by acting as plasticizers, improved the mobility of the polymer chains, which reduced the glass transition temperature and modified the mechanical properties, decreasing tensile strength and Young’s modulus while increasing elongation at break. On the other hand, the additives G and PGs had a negative effect due to thermal degradation, which decreased the strength and elasticity of the material [286].

Some authors have also studied the compatibility and effectiveness of acetyl tributyl citrate (ATBC) as a plasticizer for PHB. They investigated the effect of incorporating different amounts of ATBC (10, 20, 25, and 30% by weight) on the mechanical and thermal properties of PHB, observing how the plasticized samples exhibited lower rigidity and greater ductility than pure PHB, resulting in decreased tensile strength, reduced elastic modulus, and increased elongation at break of the plasticized samples. Also, the sample plasticized with 30 wt% of ATBC decreased the melting temperature by more than 10 °C and the Tg by more than 35 °C. However, this plasticizer considerably reduced the thermal stability of PHB, lowering its onset degradation temperature to 189 °C for the sample plasticized with 30% by weight of ATBC [287]. Parra et al. studied the effect of different amounts (2, 5, 10, 20, and 40 wt%) of PEG in PHB films obtained through dissolution in chloroform. A considerable increase in elongation was achieved at breakup to 32% with 20 wt% by weight of PEG. Furthermore, the sample with 10% by weight of PEG reduces the melting temperature by 21 °C and reduces the crystallinity of the material, increasing the permeability to water vapor and also increasing the degradation rate [288].

PHAs, as polyesters, undergo aerobic and anaerobic degradation following the mechanisms described previously.

In particular, since this kind of polymers are synthesized by bacteria in excess of the carbon source when the external carbon source is exhausted, the microorganisms themselves release enzymes called PHA depolymerase, which are able to hydrolyze PHA chains into smaller products soluble in water that can be absorbed by cells and metabolized [289].

The degradation of PHAs has been studied in soil, river and seawater, sludge, and compost [290,291] but also in phosphate buffer, simulated body fluids, and muscular tissue [292,293].

Ong et al. [294] studied the degradation of films of four different types of PHAs, poly(3-hydroxybutyrate) [P(3HB)], poly(3HB-co-8 mol% 3-hydroxyhexanoate) [P(3HB-co-8 mol% 3HHx)], P(3HB-co-12 mol% 3HHx) and P(3HB-co-21 mol% 3HHx) with three different thickness. The films were buried in a secondary forest for 8 weeks, and the soil bacterial population was studied and directly correlated with the degradation of PHA, observing a richness in PHA-degrading bacteria close to the sampling sites.

Borelbach et al. [295] investigated the degradation of PHA blended PLA nanofibers in soil for 12 weeks and also the degradation in compost at 58 °C for 4 weeks and evaluated the degradation by studying the variation in morphological, mechanical, and thermal properties.

Komiyama et al. [296] analyzed the degradation of poly[(R)-3-hydroxybutyrate-co-(R)-3-hydroxyvalerate] (P(3HB-co-3HV)) powder and processed as cast film, undrawn fiber and fivefold-drawn fibers in seawater and freshwater. They observed that powder showed faster degradation with respect to the other materials, and fivefold-drawn fibers showed slower degradation with respect to undrawn fibers due to the higher crystallinity.

Furthermore, they observed that the biodegradation of the materials depends on the amount of bacteria present in the degradation medium.

Few works have been focused on tuning the degradation rate of PHAs by blending with other polymers. Kim et al. [297] achieved to accelerate degradation rate of poly(3-hydroxyundecenoate) (PHU), which is a mcl-PHA with a long side chain that makes it highly hydrophobic, by blending with poly(lactide-co-glycolide), PLGA. The higher compatibility of PHU with blood proteins was combined with the better mechanical properties and higher degradation rate of PLGA, obtaining a material suitable for biomedical applications.

PHB is the most common PHA and can be degraded by microorganisms such as *Alcaligenes*, *Comamonas*, and *Pseudomonas* [298].

PHB degradation has been studied in different environments, such as freshwater, soil, composting, landfills, and sludge, and different strains able to degrade PHB have been studied [299,300]. PHB degradation was also studied in other conditions, such as hydrolytic and thermal degradation [301,302].

Sabapathy et al. [303] studied the degradation of PHB-sugar cane bagasse (SCB) in soil and water, which showed a degradation between 7 and 18 weeks.

Rech et al. [304] prepared films of PHB loaded with eugenol by solvent casting and studied the degradation for 60 days in agricultural, sandy, and landfill soil, observing a higher degradation rate in agricultural soil due to the higher presence of microorganisms and phosphorus availability.

Pei et al. [305] evaluated PHB degradation in municipal waste-activated sludge over two weeks at different pH, 5.5, 7.0, and 10 at different temperatures (25, 37, and 55 °C).

Brzezinka et al. [306] incorporated three types of polyhexamethylene guanidine (PHMG) derivatives in PHB and studied biodegradability in compost and river water. They were able to isolate the strains present in the film; *Bacillus toyonensis* HW1 and *Variovorax boronicumulans* HK3 were identified.

Cho et al. [307] studied six bacteria present in marine soil, in particular *Bacillus* sp. JY14. After 14 days of incubation, *Bacillus* sp. JY14 reached almost 98% of PHB degradation in solid and liquid conditions.

**Figure 14:**
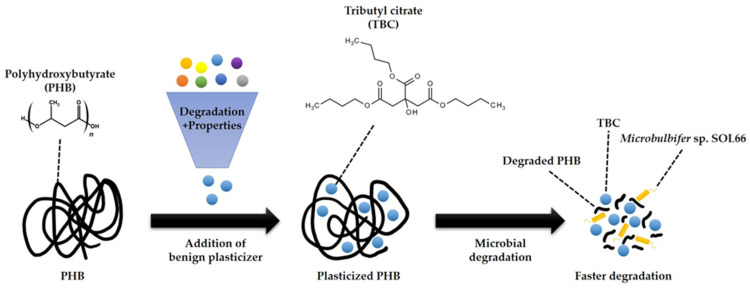
PHB degradation by *Microbulbifer* sp. SOL66 [308].

Furthermore, they studied the microbial degradation of plasticized PHB with 10% and 20% tributyl citrate by using *Microbulbifer* sp. SOL66 [308] (Figure 14).

The presence of plasticizers increased the degradation rate of PHB by 88% after 1 day. However, the samples with and without tributyl citrate presented similar degradation after 3 days.

ATBC as a plasticizer was also used by Mosnáčková et al. [309] in order to study the hydrolytic degradation of a blend constituted by PLA and PHB reinforced with hydrolyzed keratin showing faster degradation at 60 °C.

Zhou et al. [310] fabricated an artificial peripheral nerve conduit (PNC) through electrospinning of PHB/chitosan (PHB/CS) composite polymers and studied the hydrolytic degradation at different pH, observing a decreasing degradation rate from acidic > alkaline > PBS.

Râpă et al. [311] studied the in vitro degradation of PHB/bacterial cellulose (BC) in PBS of 7.4 for 20 days. The reduction in crystallinity caused by the presence of BC increased the degradation rate of PHB.

The degradation of PHBV was studied in different environments, such as soil, compost, freshwater, seawater, hydrolytic degradation, enzymatic, and aerobic composting [312].

Since PHBV is widely used in food-packaging applications, a lot of work has been focused on soil degradation.

Kim et al. [313] studied the degradation of PHBV in soil with 100% and 40% RH. PHBV was totally degraded in 100% RH soil after two weeks, showing a reduction in mechanical properties already after 3 days, while showing small changes in mechanical and thermal properties, crystallinity, and MW over six weeks in 40% RH soil.

Furgier et al. [314] studied the degradation in the soil of PHBV reinforced with three different reinforcements derived from organic waste, human hair waste (HHW), sawdust (SD), and chitin from shrimp shells for five months. The organic reinforcement is able to increase the biodegradation of the composite, showing 35% weight loss with HHW, with respect to neat PHBV (only 5% weight loss).

Furthermore, Hernández-García et al. [315] studied the degradation of films based on PLA/PHBV 75:25 and 2% (*w*/*w*) of ferulic, p-coumaric or protocatechuic acid and 15 wt% of polyethylene glycol (PEG 1000), in composting conditions during 35 and 45 days. PLA-PHBV films were degraded already after 20 days, while films with p-coumaric and protocatechuic after 21 and 26 days retarding the biodegradation.

Bonnenfant et al. [316] studied the biodegradability of PHBV reinforced with acid and quercetin. The degradation was studied in both culture media of *Streptomyces exfoliates* and in compost. Both polyphenols are able to inhibit bacterial growth, however, without preventing the mineralization of the material within the time established by the NF T51 800 standard [317].

Meereboer et al. [318] studied for the first time the degradation in the simulated marine environment of PHBV reinforced with *Miscanthus* (Misc) fibers and distillers’ dried grains with soluble (DDGS) using ASTM standard D7991-15 [319]. PHBV/Misc (85/15) and (75/25) showed 15 and 25%, while PHBV/DDGS (85/15) and (75/25) presented 17 and 40% more biodegradation compared to PHBV, respectively. The two biocomposites were totally degraded in 412 and 295 days, respectively.

Hammiche et al. [320] studied the effect of alfa fiber as reinforcement in the PHBV matrix. Alkali treatment of the alfa fiber surface improved the compatibility between the polymeric matrix and reinforcement. Moreover, treated composites showed higher degradation with respect to neat polymeric matrix and reinforced material without treatment in distilled and seawater.

Furthermore, hydrolytic degradation of PHBV was carried out by Patel et al. [321]. They developed a porous bone scaffold based on PHBV by utilizing Selective Laser Sintering (SLS), which was incubated in PBS for over 20 weeks. A bulk degradation mechanism was observed, and the crystallinity of the scaffold increased by around 7–15% due to a first degradation of the amorphous region and a further recrystallization of lower MW chains.

Zhu et al. [322] studied the hydrolytic degradation of PHBV reinforced with three types of cellulose nanoparticles (CNPs). The hydrogen bonds between CNPs and PHBV slow down the penetration of PBS and, therefore, the degradation of the material, in particular with 5 wt% of CNPs.

Kovalcik et al. [323] synthesized two types of PHAs through *C. necator* by using fructose and grape sugar extract to obtain PHBV-1 and PHBV-2, respectively. The enzymatic degradation of both PHAs was studied in synthetic gastric juice and PBS for 81 days with lipase. PHBV-2, which showed a lower crystallinity of almost 50% with respect to PHBV-1, also showed faster degradation, demonstrating the crystallinity-dependence of degradation.

## 5. Starch

Starch is a natural biopolymer extracted from various botanical sources. Worldwide production is mainly based on four common sources: corn, which represents 80% of the world’s manufactory for bioplastics [324], cassava, wheat, and potato. However, starch has been extracted from diverse natural sources to obtain different properties of the extracted starch and diversification of agricultural resources [325].

The structure of starch is formed by the succession of α-D-glucose linked to each other through (1 → 4) glycosidic bonds. This biopolymer presents two different structures in its matrix [326]: the slightly branched named amylose (20–30% of the structure) and the highly branched denominated amylopectin. The predominant linear structure of amylose is mainly linked through (1 → 4) glycosidic bonds with a slight presence of (1 → 6) glycosidic bonds, which allows for its helicoidal 3D structure. In the case of amylopectin, although being linked through (1 → 4) glycosidic bonds, it is also bonded through (1 → 6) glycosidic bonds, resulting in a highly branched structure with 24 to 30 residues of glucose units. Due to the presence of many end-chain points onto which enzymes can attack, this structure has a higher solubility and can be degraded more quickly than amylose [327]. The ratio of amylose and amylopectin depends on the source of extraction of the starch [328], as well as its characteristics and properties, as seen in Table 1. This ratio varies in most cases from 20 to 30% amylose and 70 to 80% amylopectin [329].

Several methods have been developed and used for the extraction of native starch from a wide variety of botanical sources; due to this reason, the ratio between amylose and amylopectin may vary from each extraction source. Of all the extraction methods reported for starch obtention, two are the most widely used.

In the first method, the botanical source is grounded, followed by its treatment with a dissolution of hydrogen sulfite 0.1 M at 50 °C for 24 h. After this treatment, the substrate obtained is grounded again with distillate water to extract all the starch present in the substrate, followed by several screenings through different mesh sizes to separate the residues from the starch. Finally, the starch slurry is centrifuged several times and dried in an oven at 40 °C for 12 h [330].

In the second method, the ground from the botanical source is treated with an aqueous solution of sodium metabisulfite 0.1 M for 30 min. This homogenate is screened through different mesh sizes, keeping the filtrate until the complete deposition of starch. This starch is recovered and treated with an aqueous solution of NaOH 0.2 M for 1–2 h and finally dried at 40 °C for 12 [331].

Starch can present different granule shapes and sizes, depending on the botanical source, as seen in Table 2. For example, some starch granules have angular shapes like corn starch, while potato granules exhibit irregular and oval shapes, and rice display pentagonal granules.

**Figure 15:**
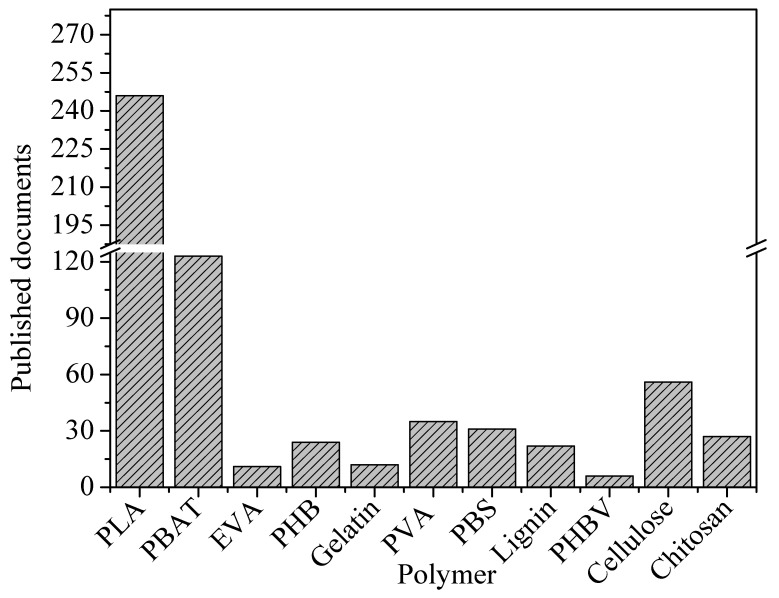
Number of publications of TPS blends (Scopus Source up to June 2024).

Due to limitations compared to conventional plastic materials, such as a high permeability to water vapor and solubility in water, a high gas permeability, and proximity to its melting and degradation temperatures, the native starch form cannot be used as a material in various industrial applications, especially the food packaging industry [332], for this reason the formation of blends based on starch are a good alternative to overcome these limitations as shown in Figure 15 using a data range from 1956 up to June 2024

**Figure 16:**
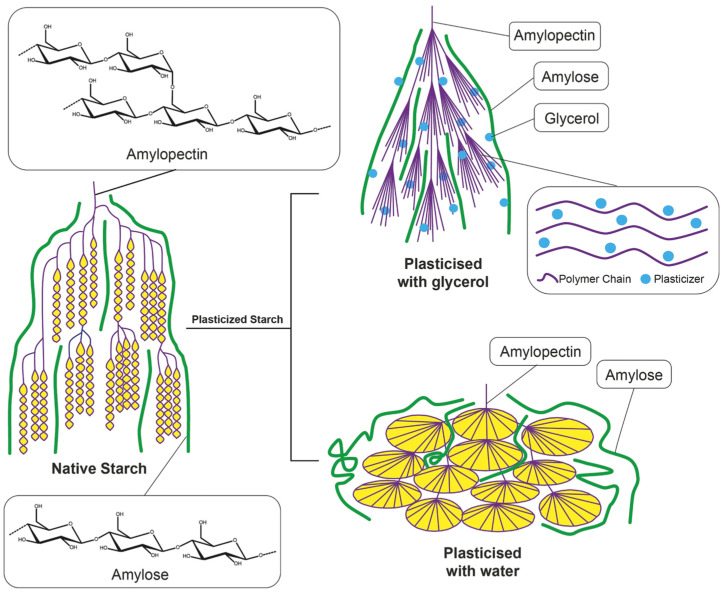
Starch microstructural changes under treatment with plasticizers or water [332].

However, starch can be processed to obtain a continuous polymeric entangled phase denominated thermoplastic starch (TPS), which can have industrial applications. To obtain TPS, it is necessary to add water and/or plasticizers [333] and have elevated temperatures and shear. The process involves various chemical and/or physical interactions such as water diffusion, starch granules’ expansion, gelatinization, and polymer melting [334]. The presence of a plasticizer changes the molecular structure of starch by destroying the inter and intra-molecular hydrogen bonding of the starch granule [244]. Meanwhile, in the gelatinization process, heat and pressure destroy the crystalline structure of starch, which transforms into amorphous structures with the presence of water and/or plasticizers [335], as shown in Figure 16.

Solvent casting is the most widespread processing method on a laboratory scale, as it does not require long processing times or high amounts of materials [336]. In this method, the starch matrix is heated above its gelatinization temperature, generally between 80 and 90 °C, for 15–20 min under constant mechanical stirring or ultrasonication. After this process, filler materials can be added and mixed, as well as through mechanical stirring or ultrasonication. Finally, the solution is poured into a glass or petri dish and dried in an oven to remove the solvent for 48 h until constant weight.

Despite its simplicity, practicality, and its cost-effectiveness. The properties obtained from materials processed through this technique can vary significantly between batches, influenced by environmental conditions, which can also slow the production process [337].

Extrusion is one of the common processing techniques for melting thermoplastic matrixes. A rotating extruder is utilized to turn solid pellets of a particular polymer into liquid, with a motor that assists in rotating the screw and a metal barrel that may be heated electrically. The polymer melts because of the heater’s heat energy, the frictional heat produced by the screw’s friction, and the immense quantity of heat emitted by the barrel. The L/D controls the residence time, and shear melts is the proportion of the screw’s flight length towards its external diameter. Higher L/D ratio screws allow for better mixing, longer melt residence times, and greater shear heating extruder [338].

Thermoplastic starch, TPS, is obtained after mixing starch with a plasticizer (often glycerol or water) to allow for the melting of the material at a temperature lower than the decomposition temperature of starch [339]. TPS is produced during the extrusion process as a result of the initial starch structure expanding, breaking, and melting in conditions of high temperature and shear. The partially crystalline structure of starch can be destroyed and transformed into an amorphous state. A decrease in the degree of crystallinity has been observed in various types of starch after the extrusion process as well as the granule morphology and the molecular structure of starch [340].

Several conditions of extrusion have been tested on starch matrices, and several research studies have been done on this topic. In the study carried out by González-Seligra et al., the morphology of the granules of a matrix of starch/glycerol was studied with different screw speeds. After characterizing the starch obtained, they concluded that the effect of different screw speeds was determinant on the final structure and physicochemical properties of the starch, with 80 rpm being the ideal speed for obtaining a homogeneous TPS [341]. Pushpadass et al. [342] conducted a similar experiment but used different temperatures and different plasticizers, from 110 °C to 120 °C, concluding that the temperature needed for the correct formation of TPS is intrinsically connected to the plasticizer used. Logié et al. [343] compared the mechanism of extrusion with the same conditions for different types of starch. In this case, potato and pea starch did not show significantly different results; the only essential difference was that potato starch presented larger macromolecular chain splitting.

Three-dimensional printing (3D printing) is a technology that uses computer softwares to design and print 3D models, with the material being added together (such as liquid molecules or powder granules being fused), typically layer by layer. This technique offers the advantage of producing complex shapes that would be impossible to construct by hand, including hollow parts or parts with internal truss structures to reduce weight. This technology has revolutionized the manufacturing industry.

Starch materials printed by 3D printing are characterized by a smooth surface, which can be compared to the surface observed in the filaments of oil-based polymers [344]. The suitability of starch-based materials for 3D printing applications is determined by their printability, gelling behavior, mechanical properties, and heat resistance. Often, the printability of starch-based materials can be enhanced by combining them with other materials [345].

Electrospinning is a method of producing fine fibers with diameters in the nanometer and micron range from polymer solutions to melts by electrostatic interaction. It is widely used in the food and biomedical sectors. In this process, a solution of the polymer is forced through a needle with a high voltage power supply, forming a conical droplet named Taylor cone due to this potential. When the electrostatic force overcomes the surface tension, the polymer solution is ejected from the Taylor cone towards the grounded collector. The solvent evaporates before the jet reaches the collector, and the polymer chains in the jet tend to stretch and orient themselves [346].

Starch has promising applications through this technique due to its high bioavailability and biocompatibility. Several studies have demonstrated the potential use of this polymer with other polymers. Hanaa et al. [347] successfully processed these various mixtures of starch and polyvinyl alcohol to improve the spinning ability of neat starch, concluding that the one that presented better characteristics was 40:60 PVA:Starch.

Malafatti et al. [348] obtained nanofibers with various ratios of PLA:Starch, from 10% to 50% (*w*/*w*) loaded with manganese carbonate as a source of Mg^2+^ ions used as a model fertilizer and source of Mn^2+^ ions required for plant metabolism. As a result, they found out that the addition of 20% of starch was the optimal proportion, but in general, the addition of starch into the PLA matrix showed a better affinity for water molecules, allowing better diffusional performance for application in fertilizer release, also showing characteristics capable of packaging and releasing particles.

Asl et al. [349] designed scaffolds based on a blend of PHB and starch (from 5 to 15 wt%) for bone tissue engineering. They observed that the addition of starch decreased the fiber diameters, decreased the brittleness and crystallinity of PHB, and enhanced thermal stability. The addition of up to 10 wt% starch also resulted in an improvement in mechanical and tensile strength and toughness. Also, scaffolds showed higher hydrophilic nature and higher degradation compared with neat PHB.

Preparations of TPS blends with conventional polymers were made to create novel materials that are low-cost and highly biodegradable. Much work has been done on creating PLA/starch mixes to lower the overall cost of raw materials and improve their degradability. However, the poor interfacial contact between hydrophilic starch granules and hydrophobic PLA is the main issue with this blend system [350].

Different adipate and citrate esters were used as plasticizers to produce PLA/TPS sheets by a calendering-extrusion process. The sheets were made using 30-weight percent TPS and 70-weight percent PLA plus plasticizer (*w*/*w*). The plasticizer corresponds to 10% of the PLA component. It was found that the mechanical characteristics and processability of PLA/TPS sheets are enhanced by the addition of adipate or citrate esters. Better plasticization was determined, and diethyl adipate produced lower tensile strength and young modulus and higher elongation at break among the plasticizers tested. Additionally, diethyl adipate increases the water vapor permeability (Shirai et al., 2015).

It was discovered that substituting formamide and water for glycerol as TPS plasticizers significantly increases the fluidity of TPS/PLA, which enhances the plasticization of the starch, raising the mixture’s dispersion and lowering the interfacial tension to produce homogenous blends. Additionally, the TPS/PLA blend’s qualities were enhanced by formamide and water. This was due to the PLA and TPS plasticized with formamide and water becoming more compatible and dispersed, resulting in an increase in elongation at a break of 4% while maintaining the tensile strength at 31 MPa, which was comparable to neat PLA [350].

The composition and characteristics of TPS/PLA blends where sorbitol, glycerol, and combinations of glycerol and sorbitol plasticize the TPS phase were examined. It was discovered that even in the absence of compatibilization, the sorbitol-plasticized TPS phase could be more evenly and finely distributed inside the PLA matrix. The TPS/PLA mixes that were plasticized with sorbitol showed significantly greater modulus and tensile strength. It was also demonstrated by thermogravimetric studies to be less volatile. Moreover, when compared to virgin PLA, the blends, including TPS plasticized with glycerol alone, exhibited the lowest shear viscosity. The viscosity of the blend rose when sorbitol gradually replaced glycerol. The blend viscosity for the TPS-only sorbitol plasticized material was comparable to that of virgin PLA [351].

Starch has received much attention in recent decades to produce biodegradable polymers as a potential replacement for plastics derived from petrochemicals [352]. Starch in its native state does not exhibit thermoplastic behavior. Therefore, the structure of granular starch must be broken down to employ starch in the plastics sector [353]. The molecular structure of starch granules changes to thermoplastic through plasticization, which requires (1) temperatures greater than 70 °C, (2) shear stress, and (3) the presence of plasticizers [354]. Plasticizers increase the free volume and decrease the molecular contacts between the polymer chains, increasing the polymers’ flexibility, plasticity, processability, and elongation [355].

Plasticizers with polar groups—such as OH, COOH, and NH2—are required to disrupt molecular connections between starch chains and generate hydrogen bonds [356]. Even though water is a good starch plasticizer, it may volatilize during the starch plasticization process. Therefore, the plasticizers used to obtain TPS from starch are polyols, amines, amides, and monosaccharides. Also, the combination of different plasticizers was investigated.

Glycerol is the most used polyol for manufacturing TPS because it has greater plasticizing and greater diffusion in the starch chains [357]. On the other hand, sorbitol has a lower volatility than glycerol and forms stronger bonds with starch chains, resulting in a material with high tensile stress and low elongation at break [351,358]. However, the tendency of sorbitol to migrate to the component surface and its eventual recrystallization limit its usage as a plasticizer for starch [351]. Studies using the combinations of the two plasticizers have been conducted to achieve a synergy between their respective qualities. The results indicate that a 2:1 ratio of sorbitol to glycerol in a formulation containing 42 weight percent plasticizer yields a formulation with a Tg below room temperature, adequate tensile strength, and significant flexibility [359]. Additionally, it was found that a 1:1 ratio keeps the plasticizers more tightly bonded, hindering them from migrating out of the material [360]. A promising polyol that is in the study as a TPS plasticizer isosorbide, which is non-toxic and seems to prevent TPS retrogradation. Moreover, it would allow energy savings because the temperature profile needed with this plasticizer is 20 °C lower than the employed glycerol [361].

The effectiveness of plasticizers with amine groups in plasticizing starch has been demonstrated to be greater than that of alcohols. TPS plasticized with formamide presents the highest elongation at breakage and the lowest tensile strength compared to sorbitol, glycerol, ethylene glycol, and urea, which points to a higher plasticization [362]. Urea, which is an amine, prevents starch from retrograding, but because it is a solid with no internal flexibility, the TPS that was plasticized with urea became brittle and stiff [363]. It was found that a 1:1 ratio of urea and ethanolamine in a formulation containing 30% plasticizer displayed higher mechanical properties than TPS plasticized by glycerol in the same content [364]. However, the utilization of amines and amides is limited due to their toxicity or unsuitability for interaction with food [357].

Fructose was studied as a plasticizer to produce TPS films. It was determined that in comparison to the other films—plasticized with urea, tri-ethylene glycol, and tri-ethanolamine- the fructose-plasticized film absorbed less water (110 for 30% F at 90 min). Additionally, according to the FT-IR results, fructose was able to generate stronger and more stable hydrogen bonds with the hydroxyl group of starch molecules than other plasticizers [365].

**Figure 17:**
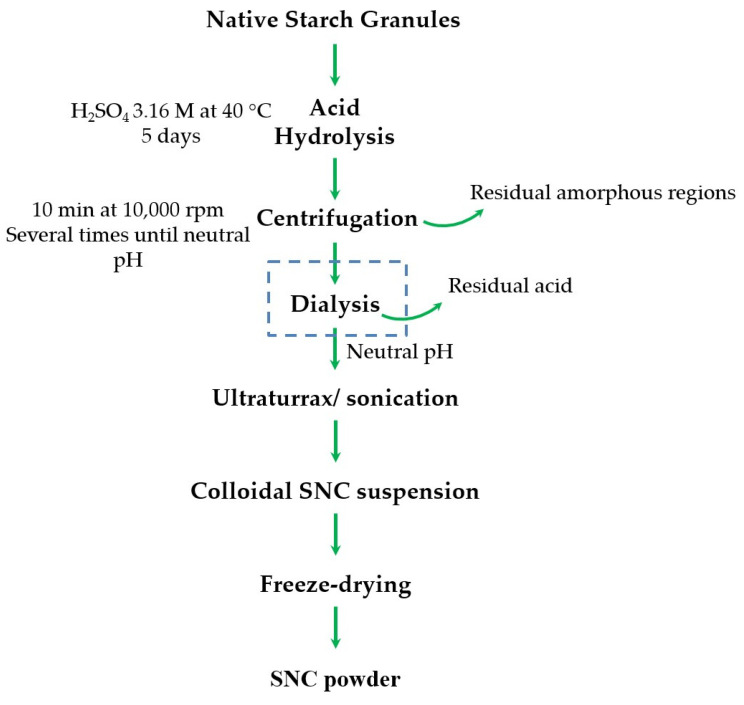
Schematic representation of an acid hydrolysis process to obtain SNCs.

Like cellulose, starch has also been used to produce nanoparticles, namely starch nanocrystals (SNCs) through acid hydrolysis. The major challenge in the production of SNC is completely removing the amorphous region of starch granules but not destroying the starch crystalline structure [366]. The classical extraction procedure of SNCs from their natural starch granules consists of the degradation of native starch granules through strong, controlled acid hydrolysis conditions, mainly using H_2_SO_4_ [367]. HCl hydrolysis is also common but requires a longer time and yields less amount of individual starch nanoplatelets [366]. The acid concentration and time of the acid hydrolysis process depend on the relative proportion of amylose and amylopectin as well as the granule morphology (i.e., size, shape, and surface state), mainly related to the botanical origin of the starch [367]. Firstly, regions of low lateral order are dissolved, and hydrolysis of the amorphous domains takes place while the highly crystalline water-insoluble lamellae remain intact [366]. The acid hydrolysis reaction takes at least 5 days. Then, the final suspension is washed by successive centrifugations and can also be dialyzed in distilled water until reaching neutral pH [336,366]. A sonication or Ultra-Turrax step is generally applied to avoid agglomeration and to improve the SNC dispersion in the final suspension [336,366]. The obtained SNCs through the acid hydrolysis process are of diverse shapes and sizes depending on the starchy plant sources as well as the preparation condition used [366,367]. Starches are categorized into three crystalline types known as A (closely packed with water molecules between each double helical structure), B (more open and water molecules are located in the central cavity formed by six double helices), and C (a mixture of both A-type and B-type) that could lead in different nanocrystals morphology [336]. Figure 17 shows a schematic representation of a classical process to obtain SNCs from native starch granules using an acid hydrolysis process.

**Figure 18:**
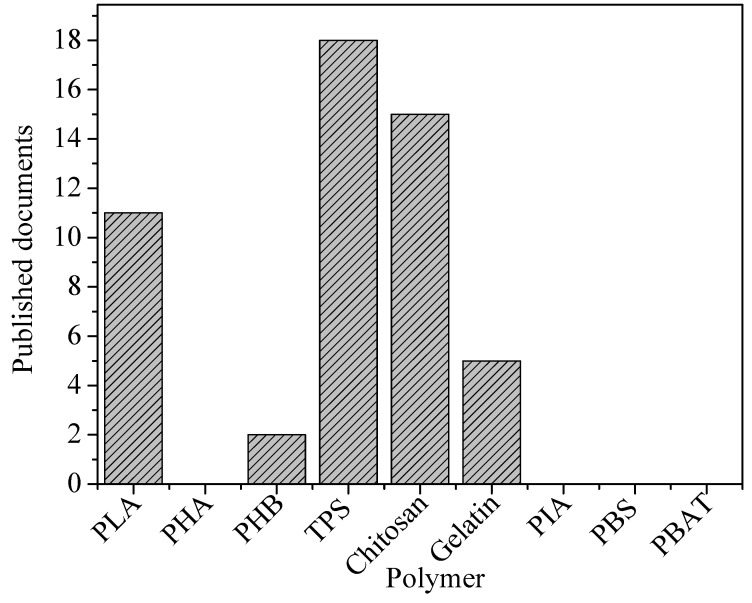
Number of publications of starch nanocrystals (Scopus Source up to June 2024).

SNCs have been used to reinforce starch polymeric matrix, improving the overall mechanical properties of the material [336]. Moreover, SNCs have also been used to reinforce biopolyester matrices, such as to promote the PHB spherulite growth [368] as well as to improve the water and oxygen barrier properties of PLA [369]. In Figure 18, the number of publications of starch nanocrystals with different polymeric matrixes is reported.

The main mechanisms in starch degradation can be divided into two: enzymes and thermal degradation.

The term ‘starch-degrading enzymes’ is often imprecise. Enzymes that act on starch granules can also utilize solubilized α-glucans. Additionally, the structural changes on the surface of native starch may facilitate the accessibility of degrading enzymes, but these processes are not yet fully understood. For this reason, we can differentiate different types of starch-degrading enzymes; most of them cleave inter-glucose bonds, although there are other enzymes that can act on inter-glucose bonds, they are functionally related to their cleavage for starch degradation [370]. The first step in the starch degradation pathway must, therefore, be catalyzed by an enzyme capable of metabolizing polymers at the surface of a semicrystalline granule rather than in a soluble form. Although several different types of enzymes are capable of releasing soluble glucans from purified starch granules in vitro experiments, no enzyme has been shown to be able to do this [371]. Among these enzymes, we can divide them into several groups in relation to the mechanism of action of the enzyme: exo-hydrolases (β-Amylases), phosphorylases, endo-hydrolases (α-Amylases), debranching Enzymes (DBEs) and disproportionating enzymes (DPE1 and DPE2).

Characteristically, this type of material presents a multi-step thermal degradation, which shows mainly two stages of its degradation. The first one is typically located at 80–120 °C, depending on its temperature with the botanical source of starch. This first loss of weight is attributed to the evaporation or dehydration of loosely bound water and low molecular weight molecules, and the second step represents around 70% of the total thermal degradation, at around 270–400 °C which corresponds with the degradation of saccharide rings [372].

## 6. Cellulose

Cellulose consists of a semi-crystalline and high-molecular-weight homopolymer of β-1,4-D-anhydroglucopyranosyl [373]. Cellulose is the most widely distributed bio-based and biodegradable polymer in nature since it is part of higher plants and available all over the world with around 8 × 10^10^ tons of annual production. It is mainly extracted from wood (50% of cellulose content) and also from cotton (90% of cellulose content), where individual crystalline cellulose chains (20–50 nm) form cellulose fibers arranged in layers bounded by a cross-linking polymer (lignin) and by strong hydrogen bonds interactions [373,374,375]. Therefore, crystalline regions of cellulose are embedded in an amorphous matrix of hemicelluloses and lignin [376], where the high amount of hydroxyl group interactions plays a major role in the cellulose crystalline packing, which governs its physical properties [377]. There are a number of cellulose derivatives that can be prepared from cellulose, including microcrystalline cellulose (MCC), a purified pulp of cellulose that is obtained by acid treatment of the alpha cellulose (type I_β_) coming from fibrous plants; it is partially depolymerized cellulose [373] with dimensions ranging between 10 and 50 µm [378]. Depending on the wood sources, MCC can exhibit significant variations in structural organization, such as crystalline region percentage or chemical composition, varying the proportions of cellulose, hemicelluloses, and lignin [373]. The degree of polymerization of the cellulose depends on the starting material, resulting in approximately 10,000 glucopyranose units in the case of wood cellulose and 15,000 glucopyranose units for cotton cellulose [374,379]. For nanocomposite production reinforced with nanocellulose (cellulosic material with at least one dimension in the nanometer scale), there are two main structures of cellulose extracted by top-down method from plants that lead to nanocellulose, nanofibrilated cellulose (NFCs) and cellulose nanocrystals (CNCs). While plants are the main source of cellulose and nanocellulose, it is noteworthy that cellulose can alternatively be biosynthesized through a bottom-up strategy from glucose with the aid of acetic acid-producing bacteria, known as bacterial cellulose (BC) [379]. Although cellulose, BC is structurally different with respect to plant-sourced cellulose, presenting a more compact network within the nanometer due to the predominant I_α_ allomorph content in its structure [380]. Cellulose is likewise found in several microorganisms, such as fungi, bacteria, and algae [375,381]. Although plant-derived cellulose is a renewable polymer, environmental concerns associated with both intensive deforestation and the chemical processing extraction methods are currently driving forces to produce cellulose in a more sustainable way, such as from microorganisms [382]. Particular attention is being paid to the obtention of BC from food waste as well as food by-products, such as from the kombucha probiotic beverage production, since the side product BC produced is considered a discarding by-product [380,383,384]. While plant-derived cellulose also contains lignin and hemicellulose that should be eliminated through strong basic and/or acid conditions, BC is mostly composed of pure cellulose, showing some advantages including higher purity, crystallinity, mechanical strength, and hydrophilicity [380], at the same time it is easily produced without the need of strong basic and/or acid extraction procedures that are questioned from an environmental point of view [383,384]. In this regard, BC can be obtained from the revalorization of food waste, introducing the process in the circular economy concept.

The classical extraction procedure of cellulose from its natural plant matrix consists of the removal of lignin, hemicelluloses, and other resins throughout an alkali treatment (2% NaOH at 80 °C) that removes the soluble polysaccharides, followed by a bleaching treatment (i.e., NaClO_2_/sodium acetate buffer pH = 4.8, 80 °C) to eliminate the majority of the matrix materials, i.e., lignin, hemicellulose or polyphenols while leaving nanocellulose intact [377,385]. Then, the bleached fibers could either be mechanical or enzymatic and disintegrated, leading to NFC, or subjected to acid hydrolysis to obtain CNC [378].

− Nanofibrillated cellulose extraction

**Figure 19:**
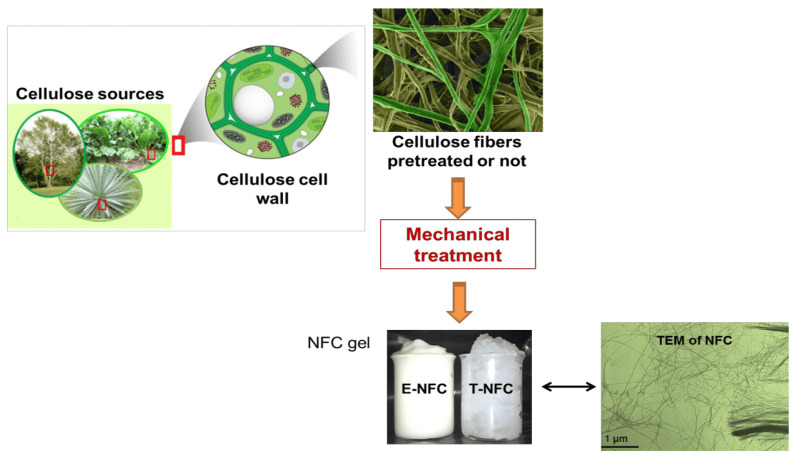
Classical process to obtain nanofibrillated cellulose (NFC). Reprinted from Missoum et al., 2013 [387] under Creative Commons CC BY license.

The mechanical (i.e., refining, grinding, high-pressure homogenization, etc.) or enzymatic disintegration of wood or cotton pulp fibers leads to a fine network of alternating crystalline and amorphous and flexible cellulose fibrils with less than 100 nm. The denominations most often used to describe such fibrils is Nano/Microfibrillated Cellulose (NFC/MFC) [386,387]. The mechanical disintegration process can also be combined with chemical or enzymatic pretreatments [374], and then the previously cut softwood fibers (0.6–0.7 mm) are homogenized under controlled temperature (70–80 °C) and high pressure (55 MPa). Then, the mechanical process itself is conducted in such a way that the applied high shear forces irreversible separate the highly ordered cellulose microfibrils, leading to a viscous aqueous suspension. This suspension is further dried with carbon dioxide, yielding isolated fibril and microfibril aggregates (diameters of about 100 nm and 2–10 nm thick), while 10–50 microfibrils compose the NFC [388,389]. Figure 19 shows a schematic representation of a classical process to obtain NFC from wood or other annual plants using a mechanical top-down approach leading to the obtention of NFC gel-like suspension [387].

**Figure 20:**
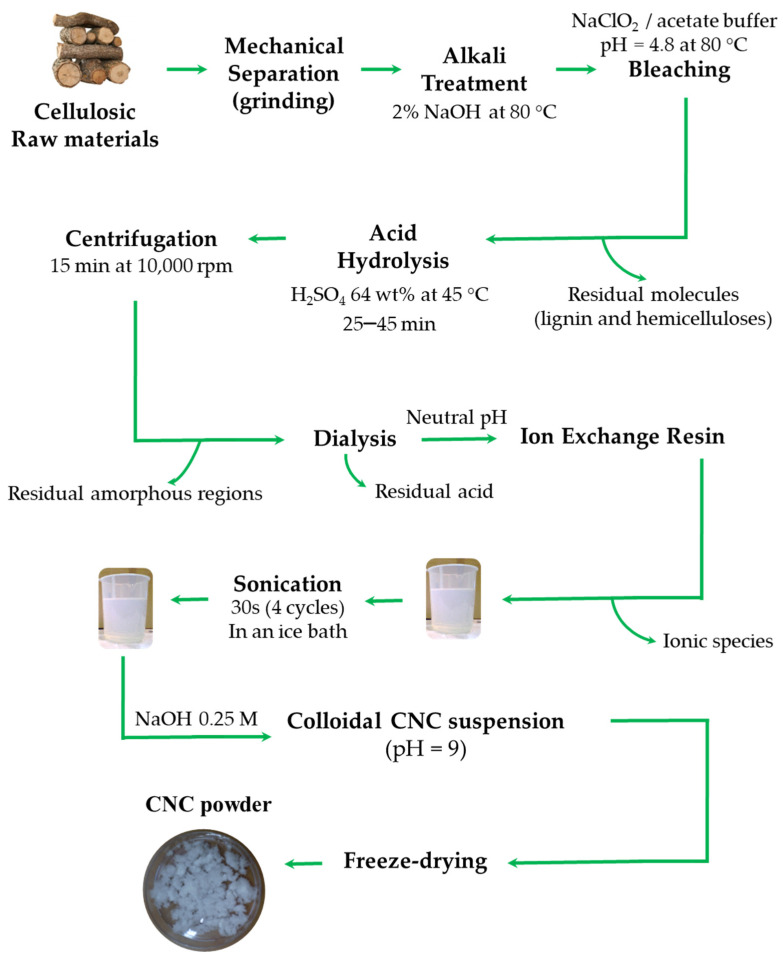
Schematic representation of pre-treatment, purification steps of the raw materials and CNC. Adapted from. Arrieta et al. [378].

The main mechanisms of extraction of nanocellulose can be summarized as the extraction of cellulose nanocrystals (CNC) by acid hydrolysis (Figure 20) and bacterial cellulose obtention.

The amorphous regions of cellulose are susceptible to hydrolysis; thus, typical procedures to produce CNC starting from natural fibers, waste, or commercial MCC, are conducted through strong and strictly controlled acid hydrolysis conditions (H_2_SO_4_ 64 wt% at 45 °C for 25–45 min) [18], yielding shorter and more crystalline fragments of MCC which, in turn, lead to the formation of highly crystalline (between 54% and 88%) and high-purity single cellulose nanocrystals (CNC) [20,373,390]. Strong acid hydrolysis conditions promote a transverse cleavage of cellulose, disrupting the amorphous regions between cellulose microfibrils, leaving the crystalline regions intact [374]. The acid is subsequently removed with deionized water through centrifugation, followed by dialysis until a neutral pH is attained. Sulfuric acid is currently chosen among other studied acids (i.e., hydrochloric acid, phosphoric, and hydrobromic acids) since it yields in nanoparticles, CNC, with negatively charged surfaces, which forms a stable colloidal suspension due to the esterification of hydroxyl groups in C6-position by sulfate ions [391]. The ionic species are then removed through an ion exchange resin with the exception of the H^+^ counter ions associated with the sulfate groups on the CNC surfaces [392,393]. Then, a controlled sonication step is generally applied to improve the CNC dispersion in the final suspension while preventing the desulfation of the sulfate groups on the cellulose surface due to overheating [377,394]. The CNC colloidal suspension is raised up to pH 9 (0.25 M NaOH), promoting the interchange of H^+^ counter ions associated with the sulfate groups with Na^+^ on the nanocrystal surface, affording CNC with improved thermal stability and with dimensions ranging from 100 to 300 nm in length and between 5 and 10 nm in width [392]. Finally, the CNC suspension undergoes a freeze-drying process to produce the nanoparticles in the form of a powder able to be processed by industrial melt processing techniques [377,378]. However, during the freeze-drying process, the intrinsic hydrophilic nature of cellulose combined with the nanoparticle’s large specific surface area and/or high surface energy promote irreversible agglomeration, leading to the formation of strong additional hydrogen bond interactions between CNC [388,395].

As was already commented cellulose can also be obtained through a bottom-up method by biosynthesis from glucose through acetic acid-producing bacteria, i.e., *Acetobacter xylinus, Acetobacter hansenii and Acetobacter pasteurianus* [375,396] in a culture medium, containing carbon and nitrogen sources under controlled temperature and pH conditions for several days, leading to microfibrils based on aggregates of relatively straight, continuous and dimensionally uniform (2 to 4 nm in diameter) glucan chains, which finally bundle to form microbial cellulose ribbons, known as bacterial nanocellulose (BNC) with a diameter less than 100 nm [388].

Concerning the revalorization of food waste, the production of bacterial cellulose from the kombucha beverage industry has gained considerable attention during the last few years due to the kombucha’s relatively high cellulose-generating capacity [383,384,397,398]. Kombucha beverage results from the fermentation of sugared infusions by a symbiotic community of bacteria and yeast (SCOBY). These aerobic microbial strains, mainly from the *Acetobacter Xylinum* family, in the kombucha culture produce the broth fermentation of a cellulosic membrane as a by-product during the infusion fermentation that keeps growing while sucrose is still available, often leading to the formation of multiple separate layers [380].

Many reports focus on the study of culture methods, fermentation techniques, media formulations, and bioreactor designs. Another strategy is the design of culture media using sugared wastes added with alcohols or organic acids at low concentrations to activate cellulose synthesis [398,399]. In addition, it was reported that there is an increase in cellulose yield production due to a synergic metabolism of yeast and bacteria, and this effect is present during kombucha fermentation. The kombucha symbiotic community of bacteria and yeast (SCOBY) ecosystem involves cooperative and competitive interactions but predominates the symbioses between microorganisms [399]. Regarding bioreactor selection, static cultures with large surfaces promote cellulose production and prevent shear stress to cells during nanofiber exportation. The surface area is directly linked to the availability of oxygen in the growth medium as dissolved oxygen, and the surface-to-volume ratio (S/V) determines the aeration conditions [398,399]. In this regard, Ramirez Tapia et al., 2023 studied the optimization of BC production during the fermentation of kombucha in yerba mate infusion. During the study, the evaluated variables were culture volume (mL): 50, 150, and 250; and initial sucrose concentration (g/L) 20, 100, and 200. Response variables were broth pH, film production (g/L), soluble solids (% d.m.), total sugar consumption (g/L), and yield conversion (g/g). results demonstrated that a lower level of volume and higher sucrose content favored the formation of the film [397].

**Figure 21:**
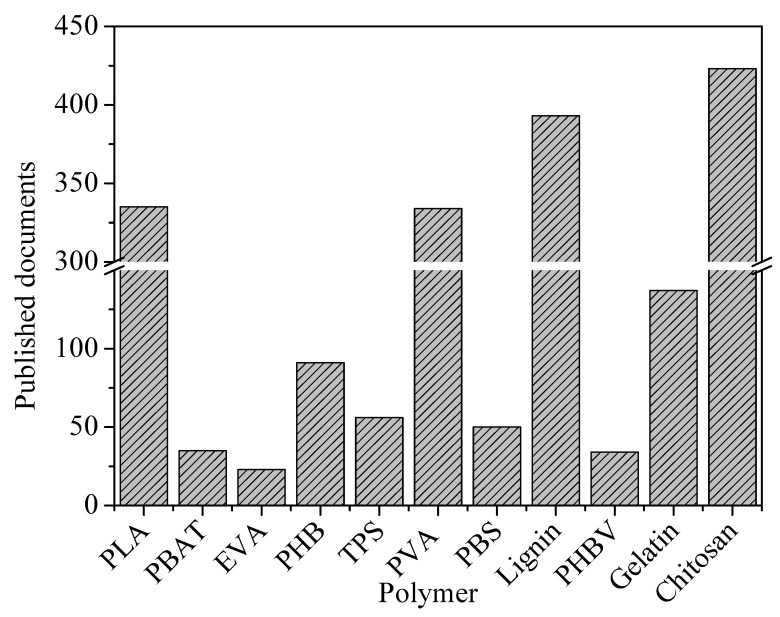
Number of publications of cellulose blends (Scopus Source up to June 2024).

With the aim to improve the mechanical, thermal, and barrier properties of other polymers, cellulose, and its derivatives have been widely blended with other biopolymeric matrices, producing polymer blends, composites, and nanocomposites with enhanced properties shown in Figure 21 using a range of years from 1943 up to June 2024.

At the industrial level, as already mentioned, cellulose-based blends, composites, and nanocomposites are mainly processed by melt-processing approaches to improve the physical properties of the final material formulation over a wide range through a readily available in the plastic manufacturing industry, simple and cost-effective processing technology. The melt processing of a polymeric matrix reinforced with cellulose tends to produce cellulose particle aggregation, which in turn leads to their phase-separation from the matrix, with a consequent reduction of the overall properties of the composite and/or nanocomposite [20,400]. As the major challenge in the production of nanocomposites is to achieve a homogeneous dispersion of the nanoparticle into the polymeric matrix, to promote the good dispersion of cellulose particles into the melt-processed polymeric matrix, lowering the surface energy of the cellulose particles is required and thus, cellulose micro and/or nanoparticles are frequently physically or chemically modified [388,395].

As lignocellulosic materials start to degrade close to 220 °C are ideal candidates to reinforce biopolymeric matrices, including biopolyesters such as semicrystalline PLA (with melting temperature between 130 and 180 °C [18,401]) and PHAs (with T_m_ between 165 and 190 °C [402]. Thus, CNC has been successfully introduced in PLA [18,392,403] and PHAs (PHB-CNC [404] and PHBV-CNC [405]) matrices leading to nanocomposites with improved thermal and mechanical properties, while it has also been used as a compatibilization strategy in PLA-PHB based blends [406,407,408]. In this regard, PLA-PHB blends reinforced with CNC have also been processed by the electrospinning technique, avoiding the PHA matrix thermal degradation [407]. Additionally, other typically used bioplastics at the industrial level, such as TPS (with Tm between 80 and 140 °C [409], have been reinforced with CNC, leading to materials with improved oxygen barrier and mechanical properties [410].

**Figure 22:**
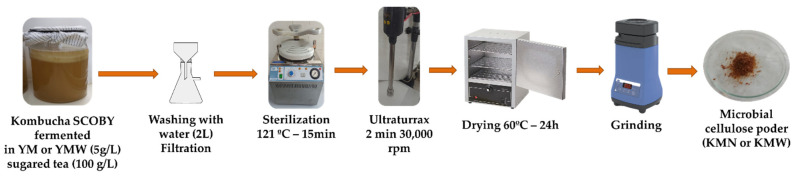
The classical process to obtain kombucha microbial cellulose. Reprinted from Agüero et al. [384] under Creative Commons CC BY license.

Agüero et al., 2023, used bacterial cellulose from kombucha fermented in wasted yerba mate (KMW) and new yerba mate (KMN) [384] as well as in spent coffee grounds (SCG), leading to spent coffee kombucha (SCK) and coffee kombucha (CK) [383] to reinforce a mechanically recycled (reprocessed 3 times, r3) PLA (r-PLA) and neat PLA matrix and further plasticized with a citrate ester (acetyl tributyl citrate) or maleinized linseed oil (MLO). The plasticized r3-PLA-ATBC matrix was then loaded with KMW and KMN in 1 and 3 wt%, while PLA was loaded in 3 and 5 wt% with SCK and CK. The strategy used to obtain bacterial cellulose in the form of powder is described in Figure 22.

The results obtained in the study suggested the potential interest in recycled and bio-based materials using food waste, which means the revalorization of food residues and their industrial application in food packaging and/or agricultural films [383,384].

Finally, the obtention of nanocomposites by a solution casting process is briefly commented on. As it has been shown, the main drawback of cellulose for its use as reinforcement in polymer matrices is its strong hydrophilic character, which means that common solvents cannot be used in the casting method. Extensive research has been conducted trying to find the more appropriate solvent, and several solvent systems have been studied: LiCl/N,N-dimethylacetamide(DMAc) [411]; Ca(SCN)_2_/water [412], LiOH/urea/water [413], chloroform/N, N-dimethylformamide (DMF) [414] and ionic liquids. Although solution casting is, in general, a simple process, the incompatibility of cellulose with polymer matrices requires the use of these non-common solvents, making the obtention of composites a major challenge. Besides, some of these solvents are not recommended due to their toxicity.

**Figure 23:**
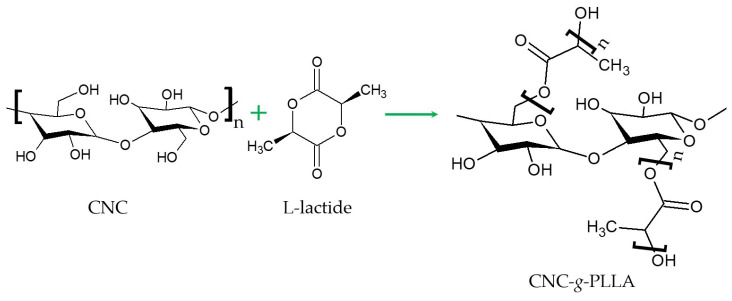
CNC functionalization with PLLA by “grafting from” reaction.

Cellulose is widely chemically modified by functionalization of its hydroxyl groups, which typically involves etherification and esterification, aimed at creating new desired characteristics, tuning the solubility, or in order to improve properties. The majority of cellulose derivatives are commercially available, and some typical examples of chemically modified cellulose are cellulose acetate (CA), methyl cellulose, carboxymethyl cellulose (CMC), cellulose nitrate, and cellulose sulfate [381]. Likewise, to improve the compatibility of nanocellulose with other polymeric matrices when producing polymeric nanocomposites, nanocellulose is typically grafted with specific groups, including the formation of esters, ethers, carbamates, and siloxanes [415]. The relatively reactive surface of nanocellulose allows for “grafting onto” and “grafting from” strategies (Figure 23). The “grafting onto” strategies are based on the attachment of previously synthesized polymer chains with reactive end-groups onto modified hydroxyl groups of the nanocellulose surface [374]. But nanocellulose is more frequently chemically modified by using the “grafting from” strategy, in which a polymer is directly grown in situ at the nanocellulose surface from the reactive hydroxyl groups, which act as initiating sites for ring-opening polymerization [374]. Navarro-Baena et al. studied the thermally-activated shape memory behavior of polyurethanes reinforced with modified cellulose nanocrystals by grafting PLLA onto their surface [416]. Peltzer et at. modified the CNC surface by grafting from L-lactide by ring-opening polymerization with the aim of improving the compatibility of nanocrystals and hydrophobic polymer matrices [417]. Sessini et al. studied the effect of the addition of polyester-grafted-cellulose nanocrystals on the shape memory properties of biodegradable PLA/PCL nanocomposites [418]. Mujica-García et al. studied the effect of the addition of functionalized cellulose nanocrystals on PLA melt-spun fibers [419]. Peponi et al. functionalized CNC by grafting from both PCL and PLA on the surface of the CNC by bio-catalysis by using an enzyme as catalyzer [420].

**Figure 24:**
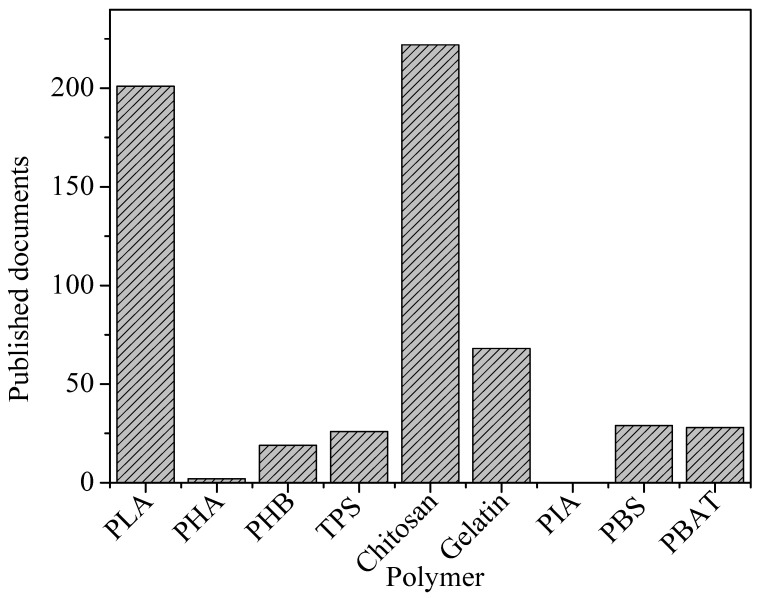
Number of publications of cellulose nanocrystals (Scopus Source up to June 2024).

Additionally, CNC can be easily physically modified by simply adding surfactants to the CNC suspension that offer both hydrophobic and hydrophilic end-groups. The CNC adsorbs the hydrophilic tails of the surfactant on its surface, and the hydrophobic head offers a non-polar surface able to lower the surface tension of the CNC, which in turn provides a better nanoparticle with non-polar polymeric matrices. Recently, Bovi et al. used bacterial cellulose and rice husk cellulose as reinforcement of PLA by means of the application of three strategies for the incorporation of the filler: direct mixing of dried milled nanocellulose and PLA, masterbatch by solvent-casting of native nanocelluloses followed by melt compounding and finally, masterbatch by solvent-casting of acetylated nanocelluloses followed by melt compounding. Masterbatches of PLA/nano celluloses were useful for the incorporation of the fillers into processing equipment due to a reduction of the hornification of cellulose during drying. Indeed, acetylation of nanocelluloses improved the dispersion of the filler. This study demonstrated that filler loading played an important role in mechanical properties, such as increasing the stiffness of the films while reducing the translucency of the materials [421]. In Figure 24, the number of publications of cellulose nanocrystals with different polymeric matrixes is reported.

## 7. Lignin

Lignin, together with cellulose and hemicellulose, is the major component of the secondary cell walls present in vascular plants. Lignin usually represents between 15% and 30% of the total dry weight and provides rigidity, water-impermeability, and resistance against microbial attack. However, lignin is usually present as a by-product of the bioethanol and wood pulp processing industries [422]. It is either discarded as waste or used as fuel [423].

**Figure 25:**
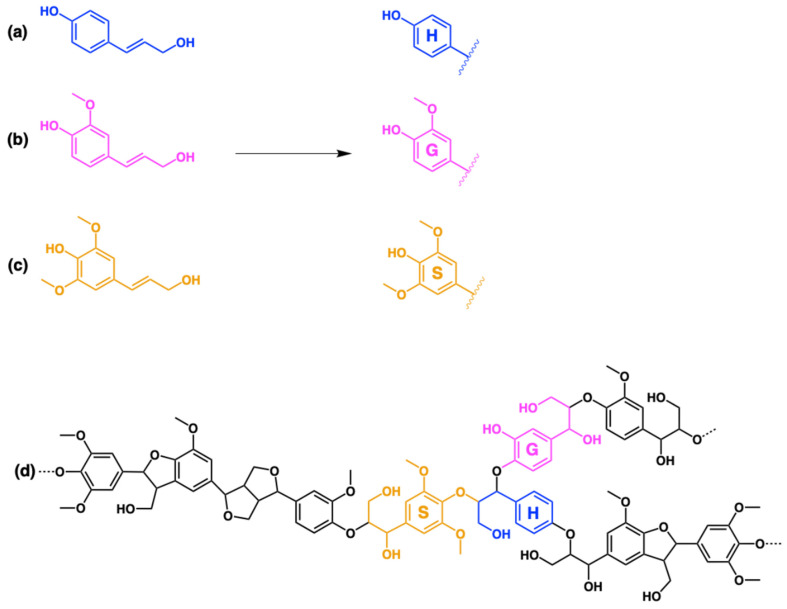
Monoligols present on the structure of lignin: (**a**) p-cumaryl alcohol and p-hydroxyphenyl; (**b**) coniferyl alcohol and guaiacyl; (**c**) sinapyl alcohol and syringyl and (**d**) lignin structure.

Lignin is a complex aromatic polymer derived from three monoligols, namely p-cumaryl, coniferyl, and sinapyl alcohols. In the polymeric form, the monolignols become individual units of p-hydroxyphenyl (H), guaiacyl (G), and syringyl (S), which are present in varying amounts and connected through several different possible C–O or C–C bonds forming a highly crosslinked and branched structure, Figure 25. The proportion of H, G, and S depends on the plant species and origin [424]. However, a small percentage of lignin building blocks are not derived from traditional monolignols, such as hydroxycinnamyl alcohols. These non-traditional building blocks include dihydroconiferyl alcohol units, as well as units with aryl conjugated carbonyl/carboxyl groups, such as vanillin, cinnamic aldehydes (conifer and sinapaldehyde), vanillic acid, and the cinnamic acids (ferulic and sinapic acid). In some lignin, acylation occurs with p-hydroxybenzoate (poplar species) or with p-coumarate (grasses) [425].

Lignification is a dynamic, flexible process reinforcing cell walls according to the different needs (water conduction, mechanical support, defense) of the plant during its life [426].

During the S1 and S2 developmental stages of the secondary wall, monolignols are initially integrated into the middle lamella of the primary cell wall. Subsequently, incorporation into the secondary wall primarily occurs after the S3 stage.

Lignification of the vessel wall, cell corner, and middle lamella begins in the early stages of xylem differentiation. In contrast, lignification of fiber secondary walls is most active during the middle and late stages.

High-resolution microautoradiography of developing hardwood xylem has revealed that the three monolignol units are incorporated at different stages of cell wall formation. During the earliest stages of S1 layer formation, H (p-hydroxyphenyl) units are primarily incorporated in the cell corner and middle lamella. The deposition of G lignin continues from early to late stages, while S lignin is mainly deposited in the middle and late developmental stages, particularly in the secondary fiber wall, after the formation of the inner layer of the secondary wall has begun [427].

The main sources of technical lignin are pulp and paper mills and emerging cellulosic biorefineries, as well as agricultural and forestry residues. The annual production of industrial lignin is about 1.8 × 10^9^–2 × 10^10^ tonnes, which is mainly obtained as a by-product from the pulp and paper industry and emerging cellulosic biorefineries, while a cellulosic ethanol plant can produce 100,000–200,000 tonnes of lignin/year [428].

**Figure 26:**
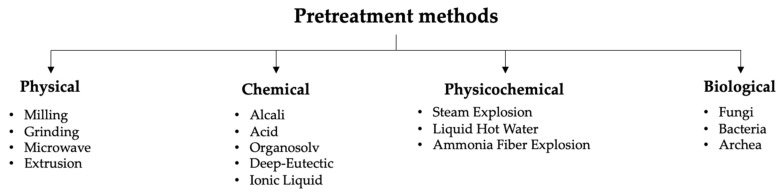
Different pretreatment methods for lignocellulosic biomass.

Many extraction methods have been used to isolate lignin from the vegetal, but not one allows for obtaining it in its native form (protolignin). Therefore, pretreatment is necessary for the valorization of the different compounds present in the matrix. The traditional pretreatment of lignocellulose aims to eliminate the hemicellulosic barrier surrounding cellulose and disrupt the lignocellulosic structure. This enhances cellulose accessibility and enzymatic hydrolysis, primarily for bioethanol production. To effectively use lignocellulosic compounds, it is crucial to select the optimal pretreatment method, considering the specific requirements of each compound and its corresponding final product. The main pretreatment methods can be divided into three major groups: physical, physicochemical, chemical, and biological, as shown in Figure 26 [429].

These methods have their advantages even though. For example, alkaline processes attack or disrupt ether bonds, like β-O-4′. The most relevant technical lignins for material applications are lignosulfonates (LS), kraft lignin (KL), organosolv lignin (OSL), and soda lignin (SL) [430]. For this reason, the combination of different techniques is a good alternative in order to extract lignin in a similar way as proto lignin can and to make the process more sustainable. Nunes da Silva et al. used a combination of an acid extraction with two different acids, in this case, phosphoric acid and sulfuric acid, followed by an alkaline delignification of the cellulignin resulting from the acid treatment [431].

Liu et al. used microwave-assisted with a ternary deep eutectic solvent composed of formic acid, lactic acid, and choline chloride. This method showed better yield and purity of the recovered lignin as well as its molecular weight, dispersity, thermal stability, and antioxidant capacity [432].

− Lignocelulosic nanoparticles extraction

**Figure 27:**
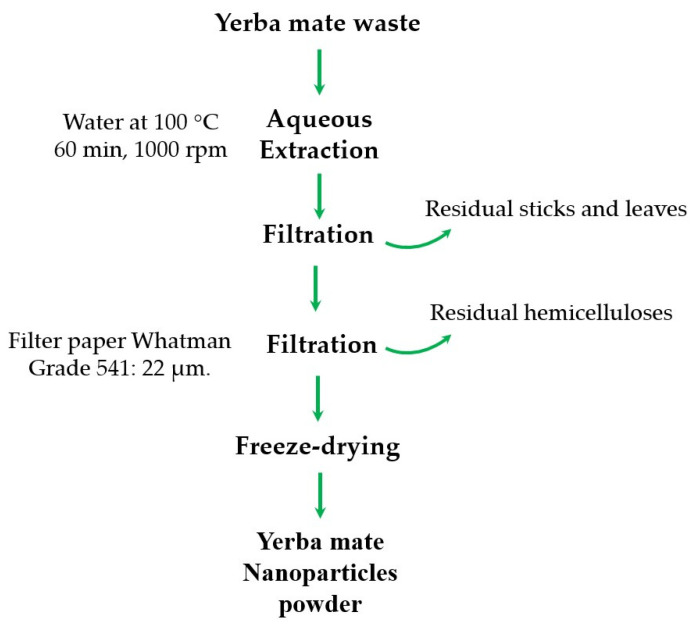
Schematic representation of the production of lignocellulosic nanoparticles from yerba mate wastes through an aqueous extraction procedure.

Lignocellulosic nanoreinforcements, have gained huge interest mainly because of their high availability. In this regard, Arrieta et al. [433] developed a simple and green method to produce lignocellulosic nanoparticles from yerba mate (Ilex paraguariensis, a typical South American infusion) waste. The lignocellulosic nanoparticles were produced by an aqueous extraction process, avoiding the use of acids and/or organic solvents. In brief, the yerba mate waste was dried after its consumption and further subjected to an aqueous extraction process (in a flask with distilled water, heated up to 100 °C under reflux for 60 min and under vigorous magnetic stirring). The obtained solution was filtered off twice (filter paper Whatman Grade 541: 22 μm particle retention), frozen, and then freeze-dried to obtain a powder. Figure 27 shows a schematic representation of the procedure used to obtain lignocellulose nanoparticles from yerba mate waste granules using an aqueous extraction process.

These lignocellulosic nanoparticles have been used to reinforce the PLA matrix, allowing the obtained materials to have improved thermal resistance and mechanical performance [433].

− Lignin nanoparticles extraction

Lignin nanoparticles (LNPs) can be obtained from various resources following different chemical/physical strategies [434]. In this sense, Gupta et al. [435] have synthesized LNPs by means of the acid precipitation method, leading to individual and randomly distributed nanoparticles. For that, lignin is suspended in distilled water and stirred for 2 h at room temperature, and then the temperature is varied between 50 and 95 °C for 4 h. Once the lignin suspension reaches the desired temperature, a 50% sodium hydroxide solution and/or an ammonia hydroxide (25%) solution are added and maintained under stirring for 2 h (the pH varies in the range of 7.5–12). Then, a formaldehyde solution (37%) is added to the reaction mixture, and the obtained LNPs are further precipitated at pH 2 (HCl, 2%), separated by centrifugation, washed, and finally dried. Two alternative methods to obtain lignin nanoparticles with very different stability upon change of pH have been compared by Frangville et al. [436]. One of them leads to lignin nanoparticles that are stable at low pH and based on the acidic precipitation of lignin from a high pH aqueous solution. The other one, based on the precipitation of low sulfonated lignin from an ethylene glycol solution through a diluted acidic aqueous solution, leads to very stable lignin nanoparticles over a wide range of pH. Lignin nanoparticles have widely been used to reinforce biopolyesters such as PLA, showing that low amounts of nanolignin (1 wt%) can be processed by melt-extrusion achieving an improvement of the mechanical properties while higher amounts lead to nanolignin aggregation [437]. Although nanolignin was less used to reinforce PHAs, there are some works in which it is evident that it is a useful nanoparticle to improve the thermal stability of PHB [438].

Lignin is a promising material for additive manufacturing due to its good mechanical properties, which gives lignin-based products good strength and durability. However, lignin has a complex structure, high molecular weight, huge steric hindrance, and low reactivity, which severely limits its applications in 3D printing materials. Researchers have focused on the depolymerization or modification of lignin to improve its reactivity [439].

Even though lignin has some interesting properties as its use in additive processing in photo-curing 3D printing material could be a photosensitive prepolymer, as can be the case of the study carried out by Nisha et al. in which lignin extracted from biomass sources was used for the synthesis of an ionic liquid-lignin epoxy prepolymer [440]. The most used 3D printing techniques for lignin processing are those based on fusion. In this study, Prakash et al. developed lignin-acrylonitrile butadiene styrene through extrusion, which was 3D printed through the FDM technique [441]. In the study by Nguyen et al., a blend of nylon and lignin was obtained through extrusion with different weight ratios of 40, 50, and 60% [442]. Or the study carried out by Tanase-Opedal et al. in which a blend of PLA/lignin with different ratios of lignin was produced by extrusion and then 3D printed through the FDM technique [443].

Due to the variance in the physicochemical properties depending on the method chosen for the extraction, several parameters have to be taken into consideration in the extrusion process:

- Structure: syringyl lignin melts easier compared to guanicyl lignin, this is due to the availability of guanicyl on the second one which can easily form condensed structures with C_5_-position of p-hydroxyphenyl. This forms β-5′ and 5-5’ linkages, which limits molecular rotation [444].
- Aryl-ether bonds (β-O-4′): It has been shown that a higher ratio of aryl-ether linkages, specifically β-O-4′, increases thermal mobility [445].
- Decomposition temperature: The greater its value, the more volatile the compound formed as a consequence of degradation during the extrusion process, which can compromise the final structure of the product [444].
- Glass transition temperature (T_g_): Glass transition temperature is related to free polymer chain movements; for this reason, lower glass transition temperatures facilitate polymer chain movement in extrusion. In the case of lignin, a higher concentration of condensation products has, as a consequence, a reduction of free volume, increasing glass transition temperature [445].

**Figure 28:**
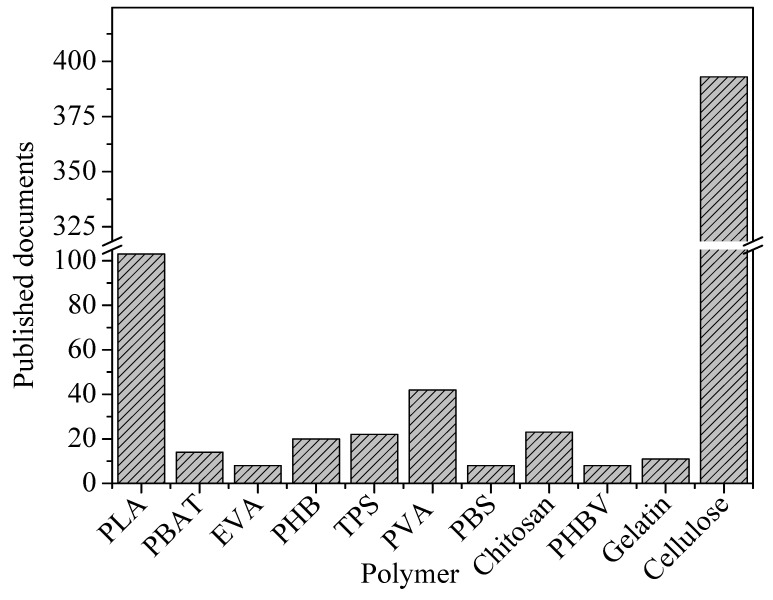
Number of publications of lignin blends (Scopus Source up to June 2024).

The increase in the compatibility of lignin with other polymer (Figure 28) matrixes is a good approach in order to enhance its mechanical and thermal properties. This strategy has been under research since 1976. In order to achieve this, several chemical modifications have been researched in the last few years using different strategies.

**Figure 29:**
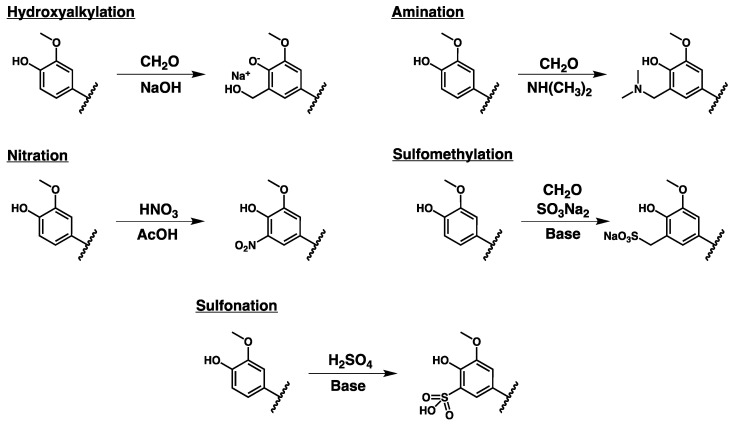
Different mechanisms for the insertion of new chemical active sites on lignin.

**Figure 30:**
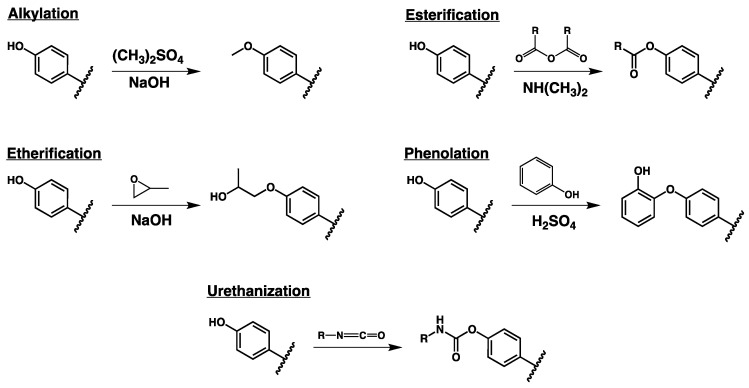
Different mechanisms for the functionalization of hydroxyl groups on lignin.

(a) Fragmentation of lignin. Due to the multiple low molecular weights in which lignin can be degraded, the use of lignin as raw material due to this fragmentation of this structure to obtain molecules with high added value like vanillin or quinines is an interesting approach for the valorization of lignin residues. Diverse techniques have been used in order to achieve this purpose, such as pyrolysis, which is based on the thermochemical decomposition of the lignin by heating biomass at 500 °C, oxidation which aims to obtain phenolic derivates using different oxidant molecules as can be metallic oxides, air, and oxygen which also permits to conserve the aromatic rings giving aldehydes as a result or gasification which is a process that converts lignin into gases, the gasification of lignin involves a series of reactions, including hydrolysis, polymerization, hydrogenation, water-gas shift, steam reforming, and methanation. These reactions are influenced by various factors such as temperature, reaction time, pressure, concentration, and catalysts [446].
(b) Synthesis of new chemical active sites. The insertion of new reactive sites can solve some of the limitations and increase the range of applications of the material. Lignin is a complex biopolymer with a variety of functional groups. Due to this reason, different strategies have been implemented, such as sulfomethylation/sulfonation, amination, hydroxylation, alkylation/dealkylation, nitration, and halogenation [447]. These mechanisms are shown in Figure 29.
(c) Functionalization of hydroxyl groups: Due to the high presence of hydroxyl groups, which are the most reactive functional groups and can affect the chemical reactivity of the newly formed material. Modifications on hydroxyl groups can lead to the formation of polyol derivatives of lignin; the most common modifications are shown in Figure 30 [446].

Lignin is the most abundant source of carbon in the soil after cellulose. Lignin degradation can play a significant role in enhancing Earth’s biofuel resources and serve as an alternative to the harsh technologies used in the paper and pulp industry [448]. Several mechanisms have been studied for lignin degradation, with bacterial and fungal degradation being the most significant.

− Fungal degradation

Several fungal species are widely spread in hardwood forests, and the principal example of a wood-degradation fungal species is white-rot fungi. They are called this because when wood decays, they cause the tree to appear bleached and light in color [449]. These microorganisms are capable of degrading lignin along with other components present in wood, such as cellulose.

White-rot fungi produce several types of extracellular oxidative enzymes. These enzymes include laccases and high redox potential ligninolytic peroxidases such as lignin peroxidase, manganese peroxidase, and versatile peroxidase. Their production is triggered by secondary metabolism conditions in response to nutrient depletion [450].

Various species of brown-rot fungi have been reported to have lignin degradation properties, although these properties are inferior to the ones observed in white-rot fungi [451]. This is because, unlike white-rot fungi, brown-rot fungi primarily target cellulose for degradation, but at the same time, they also modify the chemical structure of lignin via demethylation [452].

− Microbial degradation

Even though fungal degradation of lignin is the most studied process for lignin degradation due to the high bioavailability of fungi, it is well-known that several types of bacteria, such as *Streptomyces viridosporus* which was confirmed to degrade lignin in the 1980s by the report of Ramachandra et al. [453], are able to produce CO_2_ from lignocellulosic residues.

It was not until 2011 that Ahmad et al. characterized two DNA strains from Rhodococcus jostii RHA1 with peroxidase activity and sequence similarity to open reading frames in other lignin-degrading microbes [454]. Further dye-decolorizing peroxidases active for lignin degradation have also been identified in *Amycolatopsis sp. 75iv2* [455], *Pseudomonas fluorescens* [456] and *Thermobifida fusca* [457].

Most of the microbial mechanisms that can degrade lignin are aerobic organisms, even though there have been reported anaerobic organisms [458,459] and facultative anaerobic bacteria [460].

− Enzymes involved in lignin degradation

(a) Fungal peroxidases. All are members of class II peroxidases or plant peroxidases within the superfamily of heme peroxidases. In this class, extracellular lignin peroxidase, manganese peroxidase, and versatile peroxidase are used.

(b) Dye-decoloring peroxidases (DyP-type peroxidase). This type of heme-containing peroxidases has been recently discovered [461]; these peroxidases are inherent to certain types of bacteria, which are capable of degrading lignin along with other compounds [462].

Due to its complex structure and composition, lignin degradation is strongly affected by its nature, reaction temperature, heating rate, and degradation atmosphere, which also affects the temperature domain of degradation, conversion, and product yields [463].

Lignin decomposes at a slower rate than cellulose and hemicellulose components of biomass over a broader temperature range of 200–500 °C. This decomposition presents 3 different peaks, which correspond with β-O-4 linkages at a range between 180 and 310 °C [464], C-C linkages at a range between 310 and 415 °C [465], and β-β linkages at a range from 415 to 500 °C [466].

The degradation of the polymer structure in lignin starts at relatively low temperatures, 200–275 °C, with the main process occurring at around 400 °C, with the formation of aromatic hydrocarbons, phenolics, hydroxyphenolics and guaiacyl/syringyl-type compounds, most of the products having phenolic -OH groups [463].

## 8. Chitosan

**Figure 31:**
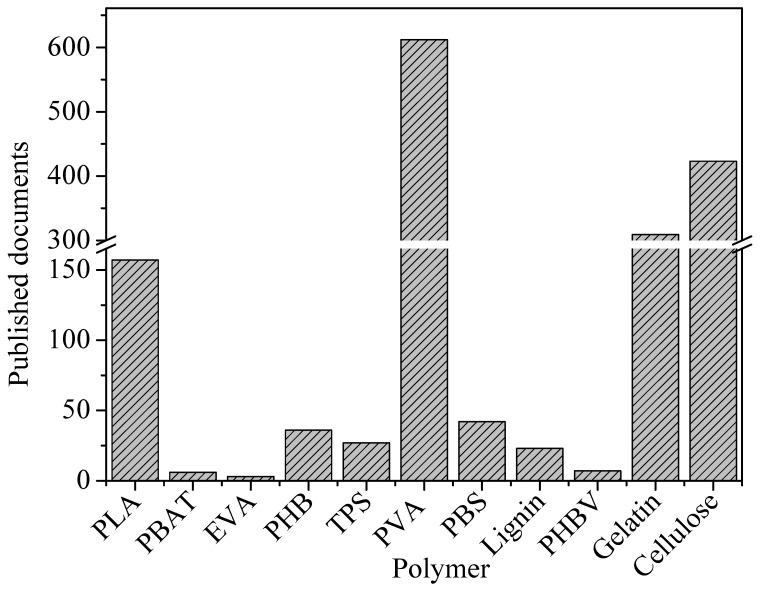
Number of publications of chitosan blends (Scopus Source up to June 2024).

Chitosan is a natural polymer widely used in environmentally friendly applications due to its naturally occurring characteristics and properties. It is a polymer obtained through the extensive deacetylation of chitinous materials, which serves as a fundamental structural component found in the outer shells of marine invertebrates: crabs, prawns, shrimps, krills, lobsters, squids and crawfish, insects: cockroaches, mosquitoes, silkworms, honeybees and microorganism: fungus, yeast and algae [467,468,469,470]. In Figure 31, the number of scientific papers from 1983 up to date on chitosan blends is shown.

**Figure 32:**
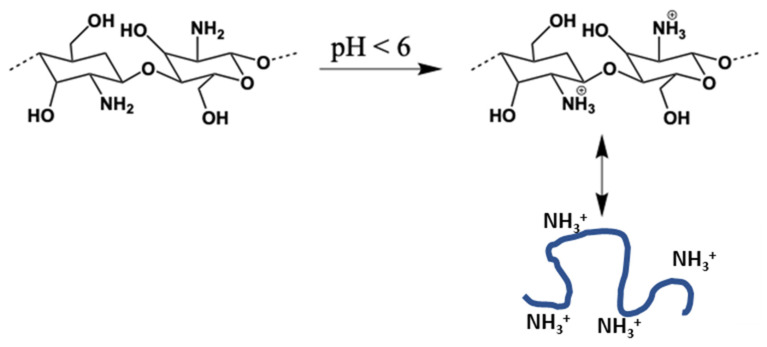
Chemical structure of Chitosan and process of transformation into a polycationic electrolyte.

Chitosan is a cationic polysaccharide with a chemical structure similar to cellulose and chitin. It is primarily composed of β(1-4) linked glucosamine units, with a certain percentage of N-acetyl-glucosamine units (Figure 32).

Chitin is made of acetamido-2-deoxy-β-D-glucose groups connected by β (1 → 4), analogous to the structure of cellulose [471]. Furthermore, it possesses acetamide units at C2. Chitosan is achieved by removing an adequate number of acetyl groups in chitin, which makes the molecule become more soluble in nearly all diluted acids [472,473]. This process is called deacetylation and leads to the discharge of amine groups to form chitosan, which is a heteropolymer with cationic characteristics, with a direct chain comprising β (1 → 4) connected 2-acetamido-2-deoxy-β-D-glucopyranose and 2-amino-2-deoxy-β-D-glucopyranose. It is important to highlight that not all of the acetamide groups in the chitosan structure are replaced by amine groups. In fact, the degree of deacetylation can range from 30% to 95%, depending on the source and production method. This deacetylation degree has a critical influence on solubility, chemical reactivity, and biodegradability [474,475].

Chitosan is soluble in dilute acidic aqueous solvents due to the addition of H+ to -NH_2_ groups. Its oligomers also dissolve under physiological conditions. However, because of hydrogen bonding and hydrophobic interactions, even completely protonated chitosan tends to aggregate in acidic conditions. The hydrophobic activity is based on the collaboration between the core polysaccharide backbone and N-acetyl groups at C2 [476,477]. The transformation of -NH_2_ groups into protonated amine groups (NH_3_^+^) within the chitosan polymer chain renders it polycationic and highly alkaline, which is responsible for its exceptional properties, including metal chelation, solubility in various media, the ability to bind with microbial cells, film-forming capacity, viscosity, optical properties, and structural characteristics [478,479,480].

The main source of Chitosan production is the extraction from shells of marine invertebrates, which are constituted by about 15–40% chitin, 20–40% protein, and 20–50% calcium and magnesium carbonate accompanied by other minor components such as lipids and astaxanthin [473]. The traditional way to produce Chitosan from Chitin using chemical methods includes 3 steps: deproteinization, demineralization, and deacetylation. Furthermore, the industrial production of Chitosan includes a fourth step, which is named decolorization (bleaching). Some authors have described that the order of the different steps, as well as the raw material used for Chitosan production, could affect extraction results. For example, for Chitosan obtained from shells with a high content of protein (i.e., shrimp), the extraction should start with deproteinization followed by demineralization [481], but if it is obtained from crab shells, demineralization with acid treatment should precede deproteinization step [482]. Other authors have reported that using both procedures indistinctly there can be obtained good results [483,484,485].

In the chemical isolation method, minerals and proteins can be eliminated from the structure of chitin by using strong acid and alkali, respectively, resulting in a huge amount of destructive acidic or basic wastewater and severe ecological glitches. Therefore, biological methods can be performed to overcome the disadvantages of the chemical isolation process. The biological methods can be divided into enzymatic reactions and microbial fermentation. The chitin produced by the biological method has a higher molecular weight and crystallinity compared to the chemical method [486,487]. Nevertheless, longer fermentation cycles and expensive enzymes are referenced as the main bottlenecks of the biological process [488].

− Deproteinization

The most common method for this is to use strong bases such as NaOH and high temperatures to render the proteins water-soluble and wash them from the chitin. Sodium hydroxide (NaOH) is the most common base used for deproteination due to its low cost. Reaction temperatures are typically between 65 °C and 100 °C and in treatment methods for 0.5 to 72 h [489]. Concerns about polluting the environment with high concentrations of sodium ions have directed researchers to alternative bases with less toxic cations such as KOH, NaHCO_3_, Na_2_CO_3_, K_2_CO_3_, Na_3_PO_4_, Ca(OH)_2_, Na_2_SO_3_, CaHSO_3_, and Na_2_S or other deproteination methodologies [490].

− Demineralization

This step typically involves exposing crustacean shells to acidic conditions to dissolve shell minerals that are then separated from the residual shell solids. Both mineral and organic acids have been employed for demineralization such as 0.5 to 5 M HCl, H_2_SO_4_, HNO_3_ or CH_3_COOH solutions at varying temperatures, from room temperature to 90 °C, and different treatment periods, from 0.5 to 36 h. Hydrochloric acid (HCl) is the most used, though concerns about environmental toxicity have prompted a turn toward organic acids such as citric, acetic, and lactic acids [491].

− Decolorization

This step aims to remove pigments and colors associated with flexible fragments of chitinous materials. Chitin is treated with various reagents such as potassium permanganate and oxalic acid, acetone, ethanol and hydrogen peroxide [492]

− Deacetylation

The degree of deacetylation plays a pivotal role in solubility, chemical reactivity, and biodegradability of chitosan. Typically, to achieve deacetylation, chitin is subjected to treatment with a concentrated alkaline solution, such as 40% to 70% NaOH or KOH, at different temperatures (ranging from room temperature to 70–120 °C) for a duration spanning from 40 min to three days. This process aims to remove acetyl groups from the chitin polymer, ultimately yielding chitosan. The caustic conditions of chemical deacetylation can lead to reductions in the molecular weight of products. Deacetylation reactions tend to last between 30 min and 5 h; some reactions last up to 24 h Longer reaction times, higher temperatures, and higher alkali concentrations correspond to higher degrees of deacetylation at the cost of molecular weight [489,490,493].

Recently, it is forcefully emerging a wide range of processing technologies involving greener solvents that are characterized by lower vapor pressure, higher thermal stability, better bonding abilities and lower toxicity compared with conventionally used solvents [494]. Ionic liquids (ILs), which are organic salts in the liquid state at room temperature (or close), can outperform the disadvantages of chemical and biological-based methods for the efficient extraction of chitin and chitosan [495].

ILs have been widely adopted in chitosan processing for pharmaceutical applications due to their ability to dissolve hydrogen bonding among chains, not affecting the molecular weight [496]. However, they have some disadvantages such as an increased cost, though some of the cost could be mitigated in the recyclability, and their hazardous toxicity and very low biodegradability [497].

As an alternative, deep eutectic solvents (DESs), recognized as the new green solvents, own similar features to ILs [498].

Deep eutectic solvents (DESs) are composed of a hydrogen bond donor and acceptor duo, having solvation characteristics similar to ionic liquids. Initially, DESs featured metal ions. However, the pursuit of reduced toxicity led to the development of natural deep eutectic solvents (NADESs), comprising primary metabolites. NADESs present a more economical and biodegradable alternative for biomass extraction. Within NADESs utilized for chitin extraction, combinations of choline chloride (ChCl) and organic acids predominate due to their minimal toxicity and capacity to eliminate calcium carbonate. Betaine serves as another prevalent hydrogen bond acceptor, complemented by various organic molecules like glycerol, ethylene glycol, and urea, acting as hydrogen bond donors [499,500].

Chitosan has found widespread application in medicine, cosmetics industry, agriculture, food processing, and paper industry, among others [501,502,503,504]. This is attributed to its remarkable properties, including biocompatibility, biodegradability, non-toxicity, antimicrobial properties, hydrophilicity, and its potential as an anticholesterolemic agent [505,506]. Furthermore, chitosan possesses distinct physicochemical characteristics, including its insolubility in water and typical organic solvents and solubility in weak acids, it has poor mechanical properties and limited water resistance. Despite these limitations, chitosan offers an excellent oxygen barrier, is readily available on a large scale, and features functional groups that facilitate chemical modifications [507].

Chitosan films have been widely studied for food-related applications for the quality preservation of a variety of food since it possesses antimicrobial activity.

Solution-casting method has been widely used to prepare chitosan-based coatings for many food applications with particular interest in fruits as it is an edible polymer. However, chitosan films have relatively high water vapor permeability and this is why lipid materials (i.e.,: fatty acids, fatty acid esters, and essential oils) are frequently added to chitosan-based formulations [508,509]. In this context, Vargas et al. have developed antimicrobial chitosan coatings with oleic acid in 1, 2 and 4% (*v*/*v*) for strawberry protection and found that chitosan-based coating with increasing amounts of oleic acid leads to better preservation of the fruit color, water resistance and mechanical performance during storage, while protected strawberries against fungal infection [508]. Similarly, Perdonés et al. developed chitosan-lemon essential oil edible coatings to extend strawberry shelf-life and observed that when using less amount of 3 wt% of lemon essential oils the chitosan coatings were able to induce fungal decay, while although showing changes in the sensory attributes maintaining pleasant changes [510].

Chitosan has been also widely used in blend formulations or as a reinforcing filler. In this regard, Bonilla et al. have developed PLA-Chitosan composites by melt-extrusion. They used different chitosan particle sizes (the starting one of 715 μm and then grinding this one to a 180 μm) and blended PLA with different amounts of both chitosan particles of 5 and 10 wt%, and observed that the chitosan presence leads to PLA-chitosan films that were able to protect minced meat samples from coliform microorganisms (about 2 log CFU/g) [511].

Arrieta et al. have prepared electrospun plasticized PLA-PHB blends with ATBC and further reinforced them with chitosan particles (chitosan particles between 50 and 100 μm) in 1 and 5 wt%. Although chitosan-loaded electrospun PLA-PHB-ATBC fibers presented some structural defect such as beads, the chitosan-loaded mats showed increased mechanical resistance at the expense of the reduction of the elongation at break [512].

## 9. Gelatin

Gelatin is an affordable and abundant water-soluble protein obtained from partial or full hydrolysis of collagen. It’s a colorless and tasteless substance whose properties vary based on the extraction process [513].

Bovine and pig skins, bones, and demineralized hooves are the primary sources of gelatin extraction. Among these, one of the primary sources of gelatin is pig skin. The entire amount of gelatin produced is made up of 46% porcine, 29.4% bovine hides, and 23.1% pork and cattle skeletons [514]. While pig skin is the most often used raw material for commercial gelatin production. Fish, mammals, or birds killed using the halal method can also be used as an alternate raw material [515]. Fish and poultry by-products are becoming more and more popular as alternative gelatin sources. Due to their high collagenous protein content, poultry wastes including blood, viscera, feet, and bone are increasingly widely utilized in the food industry [515].

**Figure 33:**
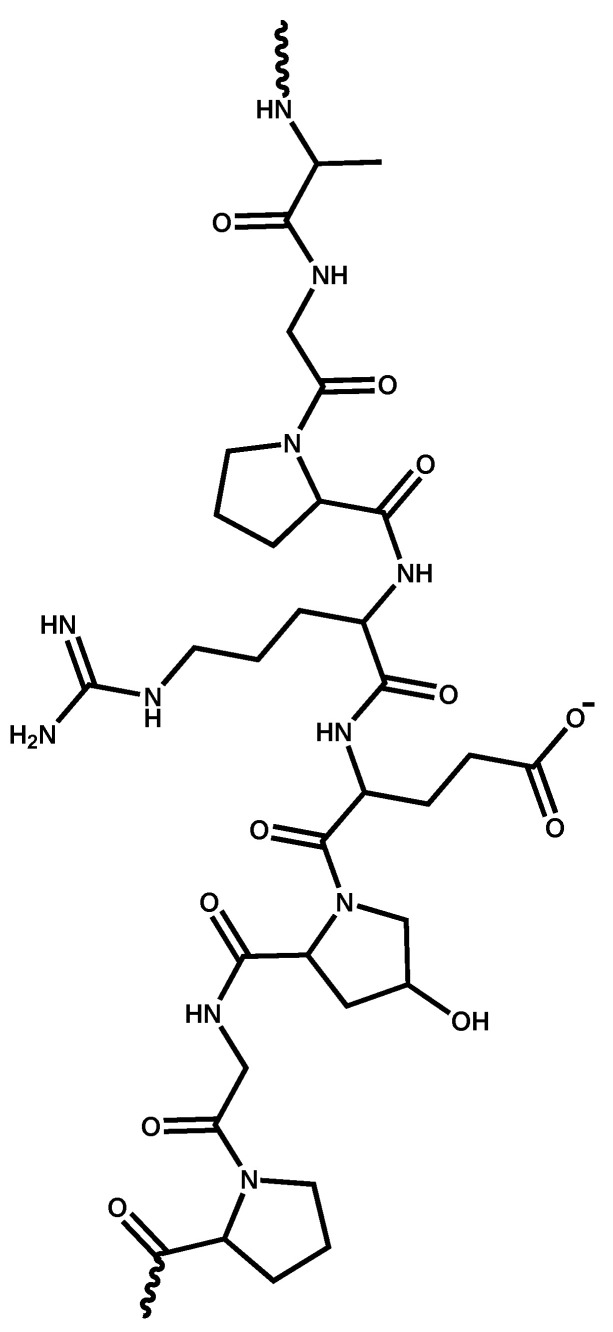
Chemical structure of gelatin.

Structure of gelatin is shown in Figure 33. Gelatin approximately contains moisture 5–8%, ash 1.9–8.9%, total lipids 1.6–4.5%, and total protein 80–91% [516].

Protein gelatin is transparent, brittle, and odorless. Its color can range from dull to slightly yellow. It appears as a tasteless sheet, flake, or powdered form. Glycerol, hot water, and acetic acid can dissolve it, but organic solvents cannot [515]. Gelatin is an amphoteric substance. Depending on the pre-treatment procedure, under acid and alkaline pre-treatment conditions, two types of gelatin are obtainable that are commercially known as type-A (isoelectric point at pH~8–9) and type-B (isoelectric point at pH~4–5) [517].

Gelatin produced from various animal byproducts has strong mechanical and barrier properties. The triple helical structure of the gelatin protein provides physical strength, and the different amino acids present in the gelatin structure easily absorb UV radiation and protect packaged food products from oxidative damage as well as water-binding ability, film- and foam-forming capacity, and emulsifying tendency [518].

**Figure 34:**
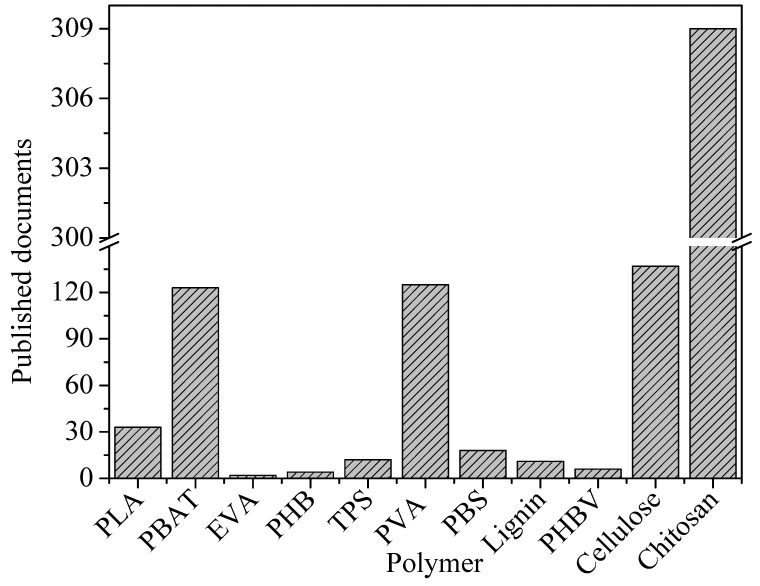
Number of publications of gelatin blends (Scopus Source up June 2024) (1968–2024).

Even though gelatin presents interesting properties for its use as material for the packaging industry, it also has some limitations for its use as raw material for this type of application, these films become fragile under drying conditions, thereby becoming rigid, brittle, and exhibit poor elongation at break. Due to the presence of polar groups on its structure, these films are highly hydrophilic, for this reason, these films present poor protection against highly moisture environments, in which they may dissolve, swell or disintegrate [519]. To overcome these drawbacks, the blending of gelatin with other matrixes has been widely explored from 1968 up to date as shown in Figure 34.

− Solvent casting

As previously mentioned, solvent casting is a technique that allows us to obtain polymeric films on a laboratory scale. In the case of gelatin, several studies have been performed in order to evaluate the properties of this polymeric matrix for its use in the packaging sector usually mixed with other bio-based polymers as PLA.

In this study, Hosseini et al. [520] successfully processed a multi-layer film based on PLA and gelatin, first the PLA layer was formed through this processing technique after which the gelatin film was cast above this first PLA film, the film-forming solution was formed dissolving 3 g of gelatin in 100 mL of water for 30 min and then heated at 45 °C for 30 min while stirring and 30% *w*/*w* glycerol.

Kurek et al. [521] produced a blend of chitosan and gelatin through this technique adding to the film-forming solution gallic acid as an antioxidant agent. Their goal was to try to produce an antioxidant material capable of storing olive oil, as a result, they obtained a material capable of reducing the amount of volatiles observed as markers of olive oil degenerative oxidation.

Zong et al. [522] developed a binary blend of starch and gelatin loaded with sweet potato anthocyanins for its use in packaging as well as a monitor of the freshness of mushrooms. They observed a change on the color of the films in function of the state of oxidation of the mushrooms as well as an enhancement of the mechanical properties of the film.

− Electrospinning

Electrospinning is an interesting technique for its use in the development of nanofiber for the packaging sector, the nanometric scale of the fibers permits the films to strongly attach to the microbial cells, destroy their cell structure, and show antimicrobial activity in the polymeric system [518].

Duan et al. [523] produced gelatin/chitosan nanofibers loaded with curcumin as a multifunctional additive, the effect of the addition of curcumin into the matrix was evaluated observing that an addition of 0.2% m/m of curcumin presented high sensitivity of colorimetric behavior to ammonia (within 3 min) improving also the antimicrobial and antioxidant activity if the matrix.

Thermal degradation is influenced by the fact that gelatin has relatively small amounts of cysteine and cystine, which contain sulfhydryl and disulfide groups, and cannot form disulfide bonds during heating; therefore, heating results in shorter polypeptide chain lengths and a decrease in particle size. An 80% decrease in gelatin particle size was observed after 1 h of heating, indicating that a high degree of degradation of gelatin had occurred [524].

At high temperatures, gelatin presents a single-step degradation in the range of temperatures from 280 °C to 400 °C [525].

## 10. Poly(Butylene Succinate)

**Figure 35:**
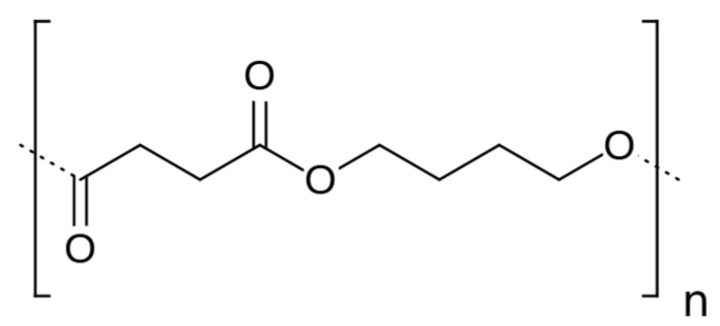
PBS structure.

Poly(butylene succinate) (PBS) (Figure 35) is a biodegradable aliphatic polyester constituted by succinic acid and 1,4-butanediol that can be obtained both from petroleum and renewable sources as starch, cellulose and glycerol [526]. PBS production involves a first esterification of succinic acid and 1,4-butanediol obtaining oligomers, followed by the polycondensation of produced oligomers to obtain higher molecular weights [527].

PBS is characterized by a Tm around 110 and 115 °C and Tg between −40 and −10 °C and good mechanical properties (elastic modulus 300 MPa and tensile strength of 20 to 60 MPa). Furthermore, PBS shows higher ductility respect to PLA and PHAs [528].

Besides the good thermo-mechanical properties, PBS can be processed with common processing techniques and is susceptible to biodegradation [529].

Degradation of PBS was studied in activated sludge, compost but also other methods as hydrolytic and enzymatic degradation [527]. Mao et al. [530] isolated *Fusarium* sp. FS1301, which is a PBS-degrading strain, by screening oil-polluted soil, and investigated the behavior of PBS, observing the degradation of both crystalline and amorphous regions. Nelson et al. [531] studied the degradation of (13C-) labeled PBS in soil in order to evaluate the formation of 13CO2 derived from PBS mineralization quantifying almost 65% of added 13C. Lyyra et al. [532] studied the hydrolytic degradation of PLA/PBS blends reinforced with bioactive glass, observing a fast loss of mechanical properties and molecular weight. Due to its biodegradability, PBS finds applications such as active packaging [533], potential biomedical applications [534], drug delivery [535], electronic devices [536], etc. In order to obtain PBS-based materials suitable for different applications, Ostheller et al. [537] fabricated PBS nanofibers through melt electrospinning, which can be used as stents, sutures and in tissue engineering. With the aim of developing a material for food packaging applications, Xu et al. [538] produced PBS-based films reinforced with chitin whiskers (CHW) and nanocrystalline cellulose (NCC) by melt extrusion, observing an increase in mechanical and barrier properties, with a reduction in oxygen transmission rate and water vapor transmission from 737.7 to 280 cc/m^2^/day and from 83.8 to 49.4 g/m^2^/day, respectively, by adding 3% of NCC. While Rennert et al. [539] produced a fully bio-based composite constituted by bio-based PBS (bioPBS) reinforced with coffee parchment (PMT) with increased antioxidant properties through injection molding.

**Figure 36:**
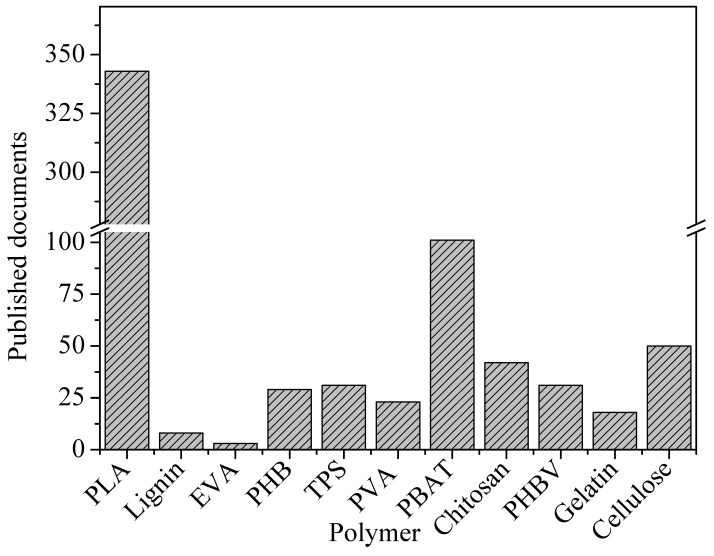
Number of publications of PBS blends (Scopus Source up to June 2024).

PBS presents low flexibility. Indeed, copolymers are produced to improve this feature. Copolymerization of succinic acid and 1,4-butanediol with adipic acid leads to the production of poly(butylene succinate-co-adipate) or PBSA [540]. However, PBSA is characterized by poor mechanical properties in terms of Young modulus and tensile strength, but its lower crystallinity and higher flexibility lead to a high degradation rate with respect to PBS [541,542]. PBSA finds application in food packaging as biodegradable active packaging, blended with other polymers such as PLA [543] and poly(propylene carbonate) (PCC) [544], or by fabricating nanocomposites using PBSA usually reinforced with layered double hydroxides (LDHs) to improve the gas barrier performance [545]. A summary of the number of publications from 1971 up to date of works related to PBS blends with other polymeric matrices, Figure 36.

## 11. Poly(Itaconic Acid)

As previously mentioned, nowadays, there is a need for bio-based and bio-degradable polymers with improved mechanical and functional properties; due to this reason, the use of alternative polymers is necessary. In this context, alternative bio-based polymers like polyitaconic acid have attracted the attention of numerous researchers over the past decade. Itaconic acid is the only one that provides the possibility of substituting acrylic acid and methacrylic acid. Furthermore, it can be used as a building block in producing essential polymers, such as resins, fibers, and rubber [546].

**Figure 37:**
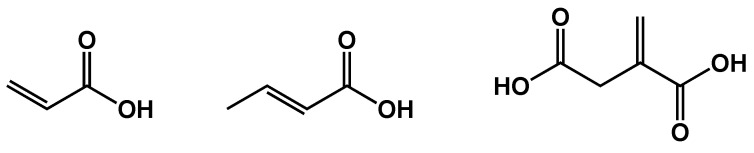
Acrylic, methacrylic, and itaconic acid structure, respectively.

Itaconic acid (2-methylenebutane-1,4-dicarboxylic acid), as shown in Figure 37, is a bio-based monomer structurally related to acrylic and methacrylic acid but polymerizes at a significantly slower rate than these monomers. Monomers derived from itaconic acid are asymmetric, having one carboxyl group on the α-carbon atom and another on the β-carbon atom relative to the double bond.

The identification of the biosynthetic pathway of itaconic acid has been known since 1936, when the synthesis of itaconic acid in Aspergillus itaconicus was reported, postulating that the decarboxylation reaction of cis-aconitate was the key process in the biosynthesis of itaconic acid. Later on, Bentley and Thiessen [547] identified the cis-aconitate dehydrogenase (CadA) in *Aspergillus terreus*, confirming that the citric acid from the Krebs cycle is the precursor in the formation of itaconic acid.

Nowadays, *A. terreus* is the strain used for the industrial production of itaconic acid, with glucose and sucrose as the main carbon source [548].

There have been attempts to homopolymerize itaconic acid via free radical polymerization since the late 50 s using potassium peroxodisulfate, dibenzoyl peroxide (DBPO), and azoisobutyric acid nitrile (AIBN) as initiators [549,550]. As a result, polymers with low molecular masses, broad PDIs, uncontrolled branched architecture and low to medium conversion were obtained. Several alternatives have been used in order to solve these downsides as can be the use of cyclic derivatives of itaconic acid [551], diaconate [552], or other derivatives can be singly substituted derivatives of itaconic [553], disubstituted itaconic acid derivatives [554,555].

As mentioned before, the synthesis of poly(itaconic acid) has severe disadvantages which have to be solved, due to this reason most of the processing of poly(itaconic acid) is produced via binary or ternary blends or modification of the polymer, those

− Electrospinning

Diverse combinations of poly(itaconic acid) with different matrixes have been studied for their processing through the electrospinning technique.

Baskan et al. [556] synthesized a copolymer of poly(itaconic acid) and polyacrylonitrile by emulsion polymerization, this copolymer was then dissolved and added a certain amount of AgNO_3_ as a source of silver nanoparticles. This mixture was then processed through an electrospinning technique using as parameters 0.8 mm needle diameter, 15 kV voltage, 1 mL/h flow rate and 10 m distance between needle tip and collector. The fibers obtained for this copolymer presented a higher diameter than the observed in polyacrylonitrile fibers and presented a reduced cyclization temperature.

Rzayev et al. [557] used a colloidal nanofibre using solution blends of polyvinyl alcohol/octadecylamine montmorillonite, an alternating copolymer of itaconic anhydride with 2-vinyl-1,3-dioxalane and their Ag-carrying complexes for its use as semiconductor electrolytes. In this case, they used the following conditions, voltage of 20 kV, flow rate of 0.7 mL/h and distance of 17 cm between needle tip and grounded collector.

Wilke et al. [558] synthesized a microgel based on poly(N-vinylcaprolactam-co-itaconic acid) for the controlled release of zinc ions in biomedical applications. Subsequently, the microgel was suspended in tetrahydrofuran, and a fixed amount of zinc chloride was dissolved in it. It was then treated with ultrasound for a few minutes and stirred at room temperature for one hour. Subsequently, 2.2 equivalents of sodium hydroxide were added, and the solvent was eliminated by evaporation. The product was washed, centrifuged and freeze-dried. The microgel was combined with PCL in a 7:3 ratio, PCL:composite. The electrospinning parameters employed were a flow rate of 0.3–0.5 mL/h, a distance between the spinneret and the target of 15–20 cm, and an acceleration voltage of 24–26 kV. These fibers presented a regulated zinc ion release during a period of up to five days, it also shows high biocompatibility proving that microfibers do not affect the activity of the human dermal fibroblasts.

− Solvent casting

Nesic et al. [559] prepared a series of films comprising chitosan and poly(itaconic acid) at varying ratios for use as a novel biosensor. These films were prepared by mixing solutions of both polymers and then cast into a petri dish, which was subsequently dried until a constant mass was achieved at 40 °C. The films exhibited superior proton conductivity in comparison to the synthetic sensor Nafion 117, as well as superior water stability.

Işıklan et al. [559] grafted poly(itaconic acid) onto poly(vinyl alcohol) with cerium(IV) ammonium nitrate as an initiator at 45 °C as a separator for acid aqueous solutions. Membranes were prepared by casting technique at 90 °C for 12 h and the cast on a petri dish. The addition of poly(itaconic acid) into the matrix improved the performance of the polymer and showed good permeation results.

Poly(itaconic acid) is well known as a biodegradable polymer, at low molecular weights poly(itaconic acid) is readily degradable in an aqueous medium, even some derivatives from poly(itaconic acid) can biodegrade faster than cellulose-based materials under compost conditions [560].

In relation to thermal degradation, a first degradation is usually observed at 100 °C as a consequence of the loss of moisture, at 170 °C a first degradation step is observed and continues up to 200 °C due to the formation of the polyanhidre, a second decomposition step started at 250 °C due to decarboxylation step of anhydrides formed in high molecular weight poly(itaconic acids) and a last decomposition step at ~450 °C correspond to the scission of the polymeric backbone [561].

## 12. Vegetable Oils as Plasticizers

**Figure 38:**
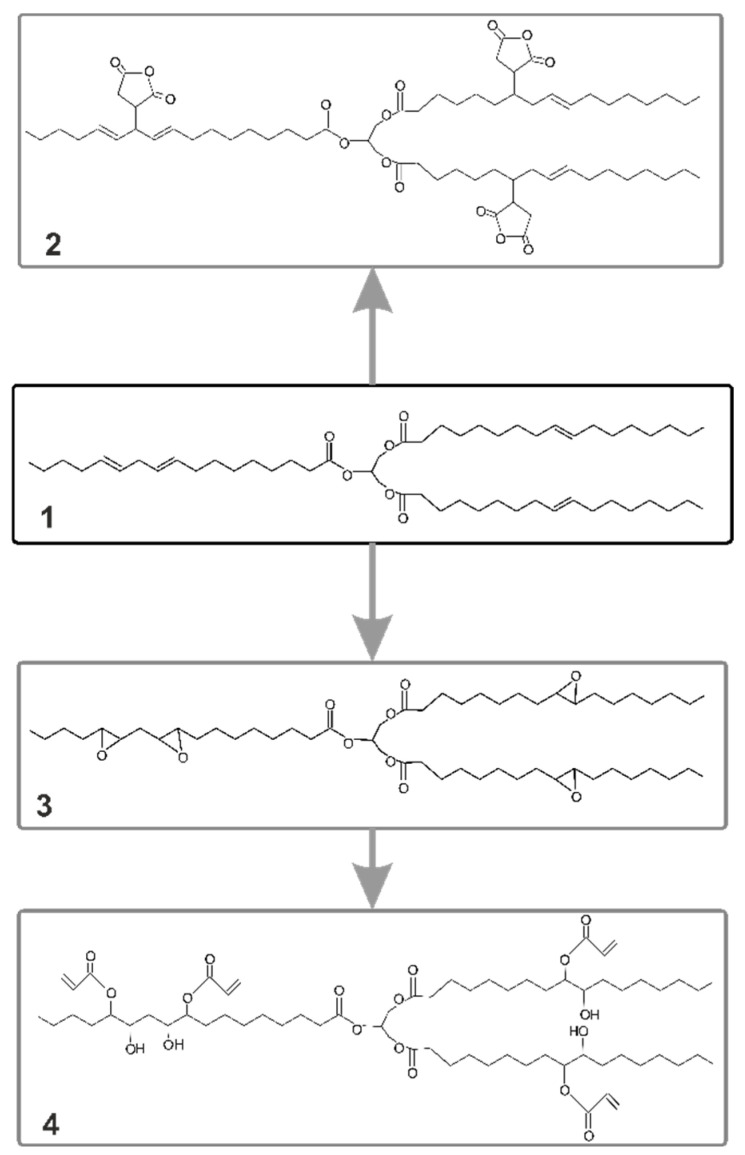
Schematic representation of the triglyceride molecule (**1**) and different chemical modifications: maleinization (**2**), epoxidation (**3**), and acrylation (**4**).

Vegetable oils, which can come from oleaginous plants and trees, are found in abundance in all areas of the planet, making them ideal as an alternative raw material for different applications, including their use as a plasticizer, since they are readily available, biodegradable and have low toxicity. These oils are primarily composed of triglycerides, whose structure can be seen in Figure 38. Triglycerides are made up of three fatty acids attached to a glycerol backbone. Most common oils contain fatty acids whose length varies between 14 and 22 carbon atoms, with between 0 and 3 double bonds per fatty acid. The distribution of fatty acids in some common oils can be observed in Table 3 [562].

Triglycerides have active sites that are susceptible to modification through chemical reactions; these sites include the double bonds, allylic carbons, the ester group, and alpha carbon atoms with respect to the ester group. These active sites can be used to introduce other groups into the triglyceride, thus obtaining molecules with greater reactivity. On one hand, the ester group composed of glycerol and fatty acids can be esterified and used as plasticizers [563]. On the other hand, a double bond can be used to modify the triglycerides and obtain molecules with greater reactivity that increase the compatibility of vegetable oils with polymers, suitable for use as plasticizing additive, as can be seen in Figure 38. From the virgin triglyceride (1), maleates (2) can be attached, or the unsaturation can be transformed into epoxy (3). Also, from the epoxidized triglyceride (3), acrylates can be incorporated using acrylic acid (4) [562].

Epoxidized soybean oil (ESBO) is one of the most studied oils as a plasticizer in various bioplastics. For example, when used as a plasticizer for PLA, it increases the elongation at break by 63% using 9 wt% of ESBO [564]. However, migration of ESBO in PLA films is observed after 30 days of storage under ambient conditions, leading to a slight increase in the glass transition temperature (T_g_) and a decrease in elongation at break and break energy. Despite this, the film exhibited suitable behavior for flexible packaging applications even after an extended storage period [91]. Another alternative is the use of epoxidized soybean oil methyl ester (ESOME), which manages to increase the elongation at break to over 700% using 40 wt%, although the maximum strength is significantly reduced. A balance between ductile and strong properties is achieved with 10 wt% of ESOME [565]. This oil has also been studied as a plasticizer in other polymers such as PBS, where using 5 wt% of ESBO results in a fifteenfold increase in elongation at break and also reduces the T_g_ [566] or for the production of plasticized ethyl cellulose films, with concentrations ranging from 15–25 wt% of ESBO, achieving a balance in mechanical properties and better thermal stability, as well as non-flammability [567]. In addition to its plasticizing effect, it also has a compatibilizing effect in blends, as is the case with PLA/PBAT blends [568] or ternary blends of PLA/PCL/PHB [281].

Another chemical modification that can be applied to soybean oil is acrylation through peroxidation. Acrylated epoxidized soybean oil (AESO) also shows a plasticizing effect on PLA, as there is an improvement in ductile properties such as elongation at break and impact energy absorption, along with a reduction in the glass transition temperature (T_g_) with contents ranging from 2.5 to 7.5 wt% in PLA. These properties favor the use of these combinations for applications in rigid materials with energy absorption capacity [569]. Like ESBO, AESO also exhibits a compatibilizing effect, in this case between PLA filled with orange peel flour. This helps to enhance the interfacial adhesion between the filler and the polymer matrix, improving the overall properties of the composite material [570]. Ozonized soybean oil (OSBO) has also been explored. This modification involves applying an ozone stream to a mixture of soybean oil and ethylene glycol, which causes the double bonds to react, thereby increasing the oxygen content in the triglycerides. Although OSBO enhances the ductile properties of PLA, such as impact resistance and elongation at break [571], its plasticizing effect is less significant compared to using ESBO or AESO.

Epoxidized linseed oil (ELO) has also shown significant interest in its plasticizing properties. Comparing the plasticizing properties that ELO and ESBO provide to PLA, it is observed that both improve ductile properties such as elongation at break and impact resistance, and both oils decrease the glass transition temperature (T_g_). Additionally, they also exhibit a compatibilizing effect between PLA and sisal fibers, as they improve the adhesion between the fibers and the PLA thanks to the epoxy groups. This compatibilizing effect is more pronounced when using ELO, as it contains more epoxy groups per triglyceride than ESBO [572]. The plasticizing and compatibilizing effect of epoxidized linseed oil (ELO) has been demonstrated with various cellulosic fillers and PLA, including hazelnut shell flour or kraft pulp fiber. This use of ELO enhances the mechanical properties of PLA composites by improving the integration and dispersion of the cellulosic material within the PLA matrix. The presence of ELO aids in bridging the interaction between the bio-based polymer and the natural fibers, thereby improving the overall structural integrity and performance of the composite materials [92,573,574].

Maleinized linseed oil (MLO) also shows a plasticizing effect on PLA, increasing the elongation at break and reducing the glass transition temperature. With just 5 phr (parts per hundred resin, equivalent to 4.8 wt%) of MLO, the elongation at break of PLA increases by 40.5%, and this increase grows with higher concentrations of MLO. This indicates that MLO can significantly enhance the flexibility and workability of PLA, making it more suitable for applications requiring more ductile properties [93]. Maleinized linseed oil (MLO) also exhibits a compatibilizing effect, for example, as a compatibilizing/plasticizing agent in blends of PLA with TPS (30 wt%). This combination shows a notable increase in elongation at break and impact resistance, with an increase of over 500% compared to unblended PLA, when using 6 phr (5.7 wt%) of MLO. This significant enhancement highlights MLO’s effectiveness in improving the mechanical properties of PLA/TPS blends, making it a valuable additive for producing more durable and flexible bioplastic materials [106]. The compatibilizing effect of epoxidized linseed oil (ELO) and maleinized linseed oil (MLO) is also evident in lignin/natural fiber composites, such as those found in Arboform^®^. Both oils improve impact resistance, tensile and flexural strength, and elongation at break. The best properties are observed when using 5 wt% of MLO. This enhancement can be attributed to the functional groups in MLO and ELO, which improve the interaction between the lignin matrix and natural fibers, leading to composites with better mechanical properties and more effective stress transfer across the materials [575].

Other modified vegetable oils studied as potential plasticizers in bioplastics include epoxidized cottonseed oil (ECSO). By using up to 10 wt% of ECSO as a plasticizer in PLA (polylactic acid), the elongation at break can be increased by more than 1100% compared to pure PLA. Additionally, this increases impact resistance and lowers the glass transition temperature (T_g_), showing very low migration in n-hexane [576]. Maleinized cottonseed oil (MCSO) also has a good plasticizing effect on PLA, increasing elongation at break. Its maximum plasticizing effect is achieved with a 7.5 wt% addition, and it also improves the barrier properties of PLA, making it ideal for food packaging applications [58]. Carbonell-Verdu et al. reported better plasticizing properties of ECSO compared to MLO, possibly due to the control of the CSO modification process since it was carried out in a laboratory, while MLO was an industrial product.

Epoxidized sunflower oil (ESO) has also been investigated as a plasticizer for PLA, showing that the ductile properties of PLA are improved but without achieving as good ductile properties as with ESBO. Moreover, while optimal properties are achieved with 9 wt% of ESBO, with ESO, it is necessary to increase above 15 wt% to achieve an optimum increase in properties [577]. Chia seed oil (CO), which has a high iodine value (above 190 g I2/100 g oil), also appears to be an interesting oil. For this reason, Dominguez-Candela et al. studied epoxidized chia seed oil (ECO) as a plasticizer in PLA, achieving an improvement in ductile properties, with a 700% increase in elongation at break compared to PLA [578]. Maleinized oils from Brazil nut seed (MBNO) and hemp seed (MHO) have also been studied as plasticizers for PLA, improving elongation at break by 643% with 7.2 phr of MBNO and 771% with 10 phr of MHO compared to PLA [579]. Epoxidized corn oil (ECornO) and maleinized corn oil (McornO) have also been used as plasticizers/compatibilizers in blends of PLA (75 wt%) and PHB (25 wt%), with both showing an improvement in the ductile properties of the system. The best ductile properties were achieved using 10 wt% of ECornO or 5 wt% of McornO, improving the elongation at break of the PLA-PHB blend by more than 400% and by more than 800% for neat PLA [282]. Other modified oils with interesting characteristics for use as plasticizers include epoxidized karanja oil (EKO) and epoxidized jatropha oil (EJO). Using 5 wt% of EKO relative to PLA achieves a balance between tensile strength (only a 9% reduction), elongation at break (improved by 77%), and impact resistance (improved by 42%) [580]. Using just 3 wt% of epoxidized jatropha oil (EJO) relative to PLA results in a 7000% increase in elongation at break and a decrease in the glass transition temperature (T_g_) by 3 °C [94]. MVOs appear to be promising candidates as plasticizing agents in bio-based polymers, particularly with PLA, as can be seen in Table 4.

In the other hand, MVOs also have a compatibilizing effect between PLA blends and PLA composites with cellulosic fibers. It is noteworthy that both effects are enhanced when the oils are composed of fatty acids with more double bonds, and the compatibilizing effect is better with maleinized oils than with epoxidized oils.

## 13. Future Perspectives

Nowadays, the environmental society concern and the European strategies to reduce the consumption of single-use plastics will open new opportunities to introduce biodegradable plastic formulations into the market.

In fact, according to the last report of Plastics Europe [581] the production of plastics from bio-based feedstock still accounts for only 1% of the plastics produced in Europe. However, bio-based feedstock availability is steadily increasing and plastics production from bio-based feedstock has significant potential growth. Moreover, increasing efforts in research and in the managing of plastic wastes are required to increase the circularity of polymer-based materials. From the research point of view there is a need to improve the effectiveness of the different polymerization processes to produce bio-based polymers including the wide variety of the potential biomasses available and to develop greener processing strategies. On the other hand, the management of plastic wastes must increase the efforts for the collection and differentiation of the different plastic fractions with particular attention to the separation of the biodegradable fraction to be used in compost production. In fact, following Plastic Europe recommendations [581], with mounting concerns about food waste’s environmental impact, compostable plastics present a promising solution. By supporting the quality of collected bio-waste, certified compostable products mitigate contamination of this type of waste by contributing to more effective waste management. Bio-waste collection bags, fruit labels, coffee capsules, and food-soiled packaging are all suitable applications for compostable plastics. Standardization plays a pivotal role in validating biodegradability claims. There is an increasing call for uniform requirements for industrial composting procedures, testing, and certifications. Such standards will assist plastic producers and waste managers to achieve a better sorting and recycling of bio-waste and align with shared sustainability objectives [581].

Not only materials but also processing technologies should be considered from an environmentally-friendly point of view. For example, if we want to analyze the electrospinning technique in a circular economy point of view, we have to remark that from one side, normally, no temperature is needed to electrospun the polymeric solutions with a consequence reduction of the energy consumption and from the other side we have to focus the attention on solvents. In this regard, two main approaches can be considered as significant challenges for the near future: the use of more environmentally-friendly solvents and the intensification of solvent recovery and reuse.

The current state of the art of the most used bio-based and biodegradable polymeric matrices has been reviewed here from a circular economy perspective. Their chemical structure, processing methods and their main properties as well as their degradation processes have been analyzed. Then, the most important strategies to produce blend formulations, composites and nanocomposites as well as the introduction of natural plasticizers to improve their performance for extending their industrial applications is summarized.

## Figures and Tables

**Table 1:** Corn, potato, and rice starch content.

Corn (Maize) Starch		
Content	Amount	Units
Density	1.356–1.4029	g/cm^3^
Amylose	24.64–29.4	g/100 g
Amylopectin	72–75.36	g/100 g
Crude fats	0.32–7.13	g/100 g
Crude proteins	0.38–7.70	g/100 g
Moisture content	10.45–12.7	%
**Potato Starch**		
**Content**	**Amount**	**Units**
Amylose	17–38.8	g/100 g
Amylopectin	73.42	g/100 g
Crude fats	0.06–0.20	g/100 g
Crude proteins	0.01–0.09	g/100 g
Moisture content	9–11	%
**Rice Starch**		
**Content**	**Amount**	**Units**
Amylose	32–4.1	g/100 g
Amylopectin	74.56	g/100 g
Crude fats	1.9–28.1	g/100 g
Crude proteins	0–3.0	g/100 g
Moisture content	13	%

**Table 2:** Size and shape of starch granules from different botanical sources.

Plant Source	Size (µm)	Shape
Maize	2–30	Round and polyhedral
Waxy maize	2–30	Round and polyhedral
Wheat	10 and 10–30	Discs
Waxy wheat	>10	Spherical and lenticular
Rice	<20 and 2–8	Polygonal and angular
Tapioca	5–45	Spherical/lenticular
Cassava	30	Kettle-drum truncated
Potato	<110	Oval and irregular
Sweet potato	1–100	Oval, spherical, round
Pea	2–40	Oval, spherical, round
Lotus	14–31	Oblong

**Table 3:** Distribution of fatty acids in various vegetable oils, adapted from Khot S. N. et al [562].

Fatty Acid	C:DB ^(a)^	Canola	Corn	Cottonseed	Linseed	Olive	Palm	Rapeseed	Soybean	High Oleic ^(b)^
Myristic	14:0	0.1	0.1	0.7	0.0	0.0	1.0	0.1	0.1	0.0
Myristoleic	14:1	0.0	0.0	0.0	0.0	0.0	0.0	0.0	0.0	0.0
Palmitic	16:0	4.1	10.9	21.6	5.5	13.7	44.4	3.0	11.0	6.4
Palmitoleic	16:1	0.3	0.2	0.6	0.0	1.2	0.2	0.2	0.1	0.1
Margaric	17:0	0.1	0.1	0.1	0.0	0.0	0.1	0.0	0.0	0.0
Margaroleic	17:1	0.0	0.0	0.1	0.0	0.0	0.0	0.0	0.0	0.0
Stearic	18:0	1.8	2.0	2.6	3.5	2.5	4.1	1.0	4.0	3.1
Oleic	18:1	60.9	25.4	18.6	19.1	71.1	39.3	13.2	23.4	82.6
Linoleic	18:2	21.0	59.6	54.4	15.3	10.0	10.0	13.2	53.2	2.3
Linolenic	18:3	8.8	1.2	0.7	56.6	0.6	0.4	9.0	7.8	3.7
Arachidic	20:0	0.7	0.4	0.3	0.0	0.9	0.3	0.5	0.3	0.2
Gadoleic	20:1	1.0	0.0	0.0	0.0	0.0	0.0	9.0	0.0	0.4
Eicosadienoic	20:0	0.0	0.0	0.0	0.0	0.0	0.0	0.7	0.0	0.0
Behenic	22:0	0.3	0.1	0.2	0.0	0.0	0.1	0.5	0.1	0.3
Erucic	22:1	0.7	0.0	0.0	0.0	0.0	0.0	49.2	0.0	0.1
Lignoceric	24:0	0.2	0.0	0.0	0.0	0.0	0.0	1.2	0.0	0.0
Average DB/triglyceride		3.9	4.5	3.9	6.6	2.8	1.8	3.8	4.6	3.0

^(a)^ Carbon number (C): Double bonds number (DB); ^(b)^ Genetically modified soybean oil with high oleic acid content (DuPont).

**Table 4:** Comparative properties of PLA plasticized with different modified vegetable oils.

MVO	Blending Ratio		Properties				Ref.
	PLA Grade	Plasticizer (wt%)	Tensile Strength (MPa)	Elongation at Break (%)	Impact Energy (kJ/m^2^)	Tg (°C)	
ESBO	N.I.	-	42.0	4.0	-	-	[564]
		9	57.7	6.5	-	-	
ESBO	2003D	-	≈66	≈7.5	16	55.5	[572]
		10	≈49	≈20	≈32	51.6	
ESBO	2002D	-	13	5	-	64	[577]
		20	10	34	-	59	
ESOME	2003D	-	51	2	-	71.3	[565]
		10	29	368	-	51.2	
AESO	6201D	-	66	4.1	19.5	62.8	[569]
		10	60	10.6	35.1	59.4	
OSBO	4043D	-	≈55	≈5	≈2	59.8	[571]
		10	≈40	≈10	≈2.2	50.1	
ELO	2003D	-	≈66	≈7.5	≈16	55.5	[572]
		10	≈51	≈15	≈35	49.0	
MLO	6201D	-	≈64	≈8	30.9	65.4	[93]
		13	≈40	≈74	50.3	60.8	
ECSO	6201D	-	63.7	9	30.8	66.7	[576]
		10	49.5	110	38.4	60.7	
MCSO	6210D	-	≈47	≈4	-	63.0	[58]
		7.5	≈38	≈16	-	61.6	
ESO	2002D	-	13	5	-	64	[577]
		20	11	16	-	60	
ECO	2003D	-	45	8.0	37.1	62	[578]
		10	≈34	64.5	68.3	56.8	
MBNO	N.I.	-	35.8	7.4	35.5	61	[579]
		7	≈48	52	≈46	58.7	
MHO	N.I.	-	35.8	7.4	35.5	61	[579]
		9	≈48	61	≈49	56.9	
EKO	6201D	-	46.5	8.3	36.6	61.2	[580]
		5	43.3	14.7	51.9	58.3	
EJO	4042D	-	58	5.4	-	62.8	[94]
		3	≈42	388	-	59.9	

N.I.: Not Indicated. ≈: approximate value, obtained from figures.

## Data Availability

Not applicable.
